# Mobile and Computer-Based Applications for Rehabilitation Monitoring and Self-Management After Knee Arthroplasty: Scoping Review

**DOI:** 10.2196/47843

**Published:** 2024-01-26

**Authors:** Sabhya Pritwani, Purnima Shrivastava, Shruti Pandey, Ajit Kumar, Rajesh Malhotra, Ralph Maddison, Niveditha Devasenapathy

**Affiliations:** 1 Department of Research & Development The George Institute for Global Health India Delhi India; 2 Department of Orthopaedics All India Institute of Medical Sciences Delhi India; 3 Department of School of Exercise & Nutrition Institute for Physical Activity and Nutrition Deakin University Geelong Australia

**Keywords:** knee arthroplasty, telerehabilitation, mHealth, rehabilitation, monitoring, self-management, knee, arthroplasty, social support, mHealth intervention, development, scoping review, knee replacement

## Abstract

**Background:**

Successful post-knee replacement rehabilitation requires adequate access to health information, social support, and periodic monitoring by a health professional. Mobile health (mHealth) and computer-based technologies are used for rehabilitation and remote monitoring. The extent of technology use and its function in post-knee replacement rehabilitation care in low and middle-income settings are unknown.

**Objective:**

To inform future mHealth intervention development, we conducted a scoping review to map the features and functionality of existing technologies and determine users’ perspectives on telerehabilitation and technology for self-management.

**Methods:**

We followed the Joanna Briggs Institute methodology for scoping reviews. We searched the Embase, Medline, PsycINFO via OVID, and Cochrane Central Register of Controlled Trials databases for manuscripts published from 2001 onward. We included original research articles reporting the use of mobile or computer-based technologies by patients, health care providers, researchers, or family members. Studies were divided into the following 3 categories based on the purpose: validation studies, clinical evaluation, and end user feedback. We extracted general information on study design, technology features, proposed function, and perspectives of health care providers and patients. The protocol for this review is accessible in the Open Science Framework.

**Results:**

Of the 5960 articles, 158 that reported from high-income settings contributed to the qualitative summary (64 studies on mHealth or telerehabilitation programs, 28 validation studies, 38 studies describing users’ perceptions). The highest numbers of studies were from Europe or the United Kingdom and North America regarding the use of a mobile app with or without wearables and reported mainly in the last decade. No studies were from low and middle-income settings. The primary functions of technology for remote rehabilitation were education to aid recovery and enable regular, appropriate exercises; monitoring progress of pain (n=19), activity (n=20), and exercise adherence (n=30); 1 or 2-way communication with health care professionals to facilitate the continuum of care (n=51); and goal setting (n=23). Assessment of range of motion (n=16) and gait analysis (n=10) were the commonly validated technologies developed to incorporate into a future rehabilitation program. Few studies (n=14) reported end user involvement during the development stage. We summarized the reasons for satisfaction and dissatisfaction among users across various technologies.

**Conclusions:**

Several existing mobile and computer-based technologies facilitate post-knee replacement rehabilitation care for patients and health care providers. However, they are limited to high-income settings and may not be extrapolated to low-income settings. A systematic needs assessment of patients undergoing knee replacement and health care providers involved in rehabilitation, involving end users at all stages of development and evaluation, with clear reporting of the development and clinical evaluation can make post-knee replacement rehabilitation care in resource-poor settings accessible and cost-effective.

## Introduction

Knee arthroplasty is the gold standard treatment for end-stage osteoarthritis when conservative treatments fail to relieve symptoms [1]. Wound care and postarthroplasty physiotherapy are essential components of this treatment. Poor adherence to physiotherapy could delay the recovery and lead to suboptimal functional outcomes [2]. Beyond in-hospital clinical care and initiation of physical therapy before discharge, continued and reliable access to information, support from health care providers, awareness of the recovery pathway, easy access to rehabilitation centers, and periodic monitoring are influential factors for optimal recovery [3-6]. In addition to an uneventful surgery, postarthroplasty outcomes are associated with several patient-related factors such as their preoperative physical and mental state, comorbidities, social support, and socioeconomic status, emphasizing the need for personalized approaches [7]. Hence, monitoring of the rehabilitation phase is essential, whether at clinics, in rehabilitation units, or at home [8-10].

Technology-assisted remote monitoring methods are increasingly being advocated in high-income countries. There is low to moderate-quality evidence on the superiority of telerehabilitation compared with unsupported home-based rehabilitation and noninferiority compared with clinic-based monitoring with respect to range of motion (ROM), pain, function, quality of life, and cost-effectiveness at 3 months between clinic-based and home-based rehabilitation strategies using technology [11-17]. Hence, current evidence supports the adaptation of technology-based rehabilitation as feasible, as safe, and as good as clinic-based monitoring with an additional benefit of saving out-of-pocket expenditure. Technology-based approaches are diverse, varying from telehealth [17] to virtual reality techniques [13] aimed at improving adherence to physical therapy and facilitating remote monitoring [12] of patient progress during the post-acute rehabilitation phase [18].

Therefore, the aim of this scoping review was to summarize the extent, range, and nature of technology used for provision of rehabilitation or to monitor progress following knee arthroplasty. This scoping review aimed to address the following objectives:

1. To map the characteristic features and functionality of the technologies, guiding or theoretical framework for designing the technology, and evaluation methodologies of mobile technology–based apps for rehabilitation monitoring and self-management following knee arthroplasty
2. To understand the patient and physical therapist perspectives regarding the use of mobile technology–based apps for rehabilitation monitoring and self-management following knee arthroplasty

To our knowledge, there are no existing scoping reviews that address our aims [19]. The information from this review will help us and other researchers make an informed decision on future mobile health (mHealth) interventions for monitoring post-knee arthroplasty rehabilitation care by physiotherapists and orthopedic surgeons and to promote self-management by individuals. This review will also help highlight existing gaps in the context of low and middle-income countries (LMICs).

## Methods

We conducted this scoping review following the Joanna Briggs Institute (JBI) methodology for JBI Scoping Reviews [20] and consulted the PRISMA-ScR (Preferred Reporting Items for Systematic Review and Meta-Analyses Extension for Scoping Reviews) checklist for reporting [21]. The protocol was registered at the Open Science Framework [22].

### Data Sources and Searches

To identify relevant studies, an electronic database literature search was conducted in the Embase, Medline, PsycINFO via OVID, and Cochrane Central Register of Controlled Trials (CENTRAL) databases using the following key terms: “Knee arthroplasty OR Knee replacement,” “mobile,” “web,” “remote sensor,” “computer,” “telerehabilitation,” and “m-health” (Tables S1 and S2 in Multimedia Appendix 1). The search was executed in October 2021 and updated in August 2023. The search was restricted to 2001 onward. There were no language restrictions during the search. We searched the reference list of included articles to identify potentially eligible studies.

### Study Selection

Predefined inclusion criteria were articles reporting the use of mobile or computer apps or any other technologies such as sensor-based devices for delivering or monitoring rehabilitation either scheduled or following knee joint replacement. We also included proof- of-concept papers that described the development process of a mobile or technology-based solution for rehabilitation. The purpose of technology could be for a health care provider to monitor rehabilitation adherence, to aid patient-health care provider communication, to promote self-management, to act as reminders, or to act as a source of education or any other function that is aimed at rehabilitation care following knee replacement. The app or technology could be used by patients, health care providers, researchers, or a family member. Included studies could have been conducted in the community or home for any clinical setting in any geographic region. The studies were required to be original research articles, and we included experimental and observational studies using quantitative or qualitative research methods. Reviews (narrative or systematic reviews), non-English articles, and articles without abstracts or full texts were excluded.

### Data Extraction

Screening of manuscript titles and abstracts was conducted by 2 independent reviewers using the web app Rayyan [23]. Prior to screening, reviewers discussed inclusion and exclusion criteria to ensure consistency between individuals. Two reviewers assessed the eligibility of the full text, and disagreements were resolved by discussion. Systematic reviews were not included in the review but were used to obtain potentially relevant references. Multiple publications originating from a single technology were grouped and presented as 1 study.

For data charting purposes, the studies were divided into the following 3 categories: (1) studies that had no rehabilitation program but included an app or a technology to assess ROM or gait and were validation studies, (2) studies reporting the use of a mobile or computer app or a telehealth delivery platform for a rehabilitation program with or without sensor-based devices and wearable sensors, (3) studies that reported end users’ perceptions of the technology used for rehabilitation monitoring. Data on the general information for the studies, features of the technology, the proposed function, and perspectives of health care providers and patients were extracted and entered in Microsoft Excel. If only the protocol of a planned study was available, there was no information on clinical evaluation, or the study included <6 individuals, we did not extract data beyond the general information.

## Results

### Search Results

**Figure 1:**
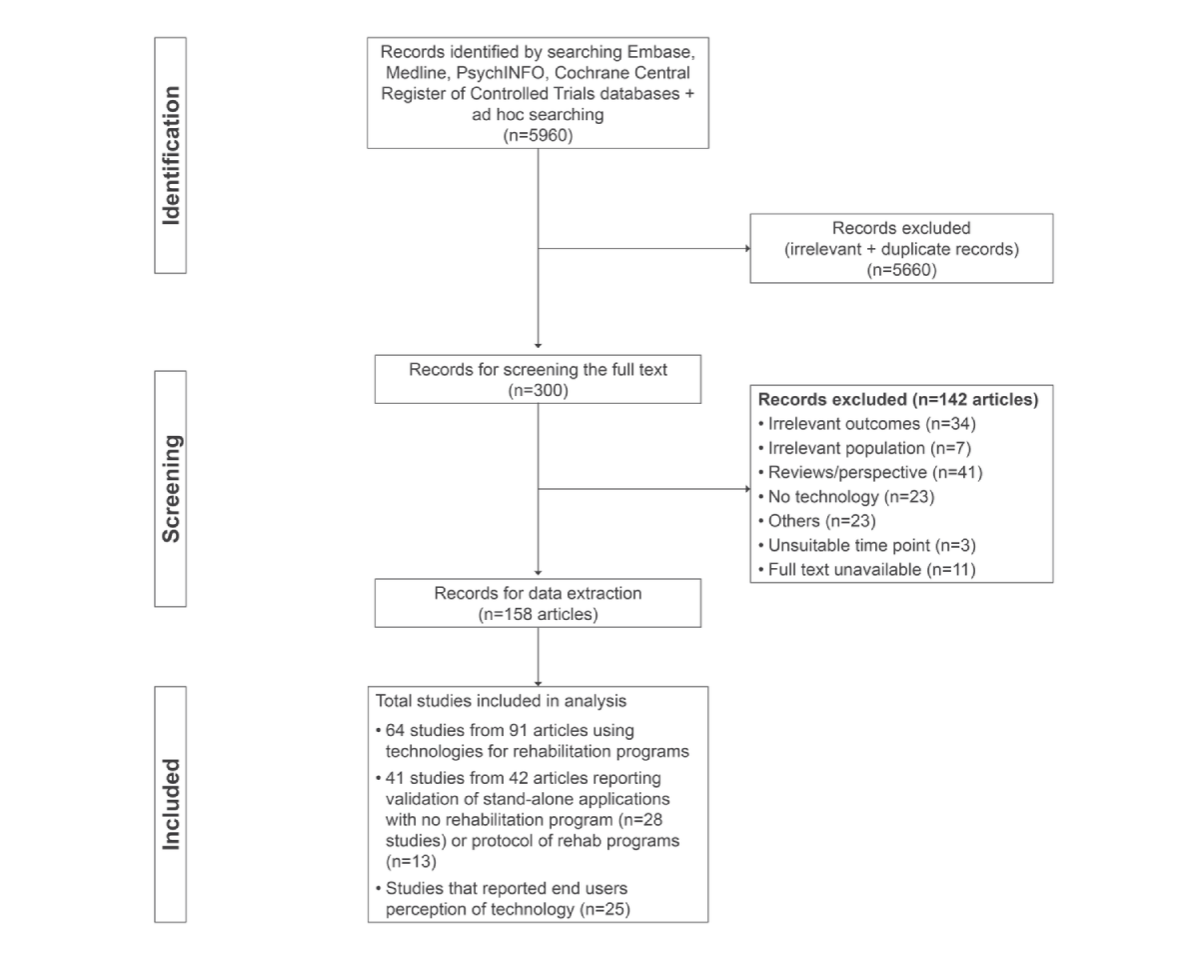
Process of identifying and including studies according to PRISMA-ScR (Preferred Reporting Items for Systematic Review and Meta-Analyses Extension for Scoping Reviews).

The database search, including the ad hoc search, yielded 5960 articles. Of these articles, 300 articles were considered potentially relevant. Of these, 158 articles were included for data extraction, 131 articles were excluded, and 11 articles were not available (Table S3 in Multimedia Appendix 1). Of the 158 articles, 91 articles (64 studies) reported the clinical evaluation of a technology-based rehabilitation program, 29 articles (28 studies) reported the validation or a proof of concept of technology intended to be used for rehabilitation, and 13 articles were protocols of evaluation studies. In addition, 25 articles reported end users’ perceptions on technology (Figure 1) as stand-alone articles or part of clinical evaluation studies (n=13), totaling 38 studies. The 13 studies that reported the perceptions of technology that were also included in rehabilitation program studies were removed from the final list of included full-text articles to avoid double counting.

### Technology for Rehabilitation

#### Characteristics of the 105 Studies

**Figure 2:**
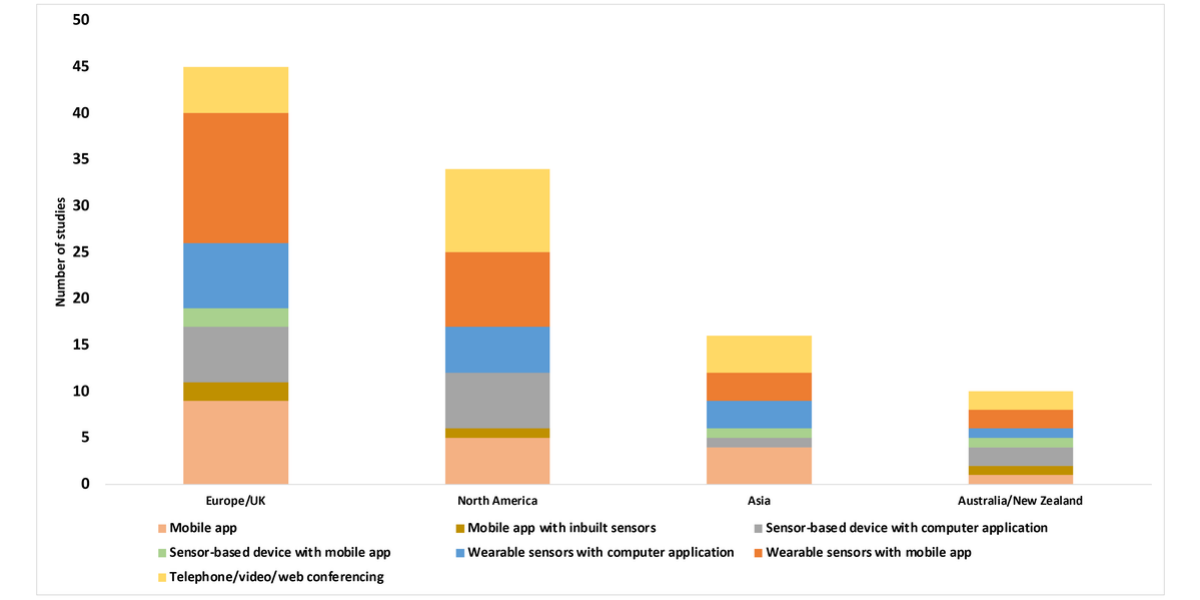
Number of studies published by region based on different technologies (n=105).

Studies were reported from Europe and the United Kingdom (n=45) [11, 24-60, 62-66, 169, 170], North America (n=34) [6, 67-99], Australia and New Zealand (n=10) [100-109], and Asia (n=16) [110-125]. None of the studies were from LMICs. Reports of mobile-based technologies represented the highest number (54/105, 51.4%) [6, 25-27, 31, 32, 36, 37, 41-50, 52, 55, 56, 58-60, 62, 64, 67-69, 74, 75, 78, 80, 82, 83, 90-92, 95, 101, 103, 106-108, 112, 113, 117, 121-126, 169], followed by computer applications (31/105, 29.5%) [24, 29, 30, 33-35, 39, 53, 54, 57, 65, 66, 70, 73, 76, 79, 84-86, 89, 93, 97, 98, 100, 102, 104, 111, 114, 116, 120, 127], and tele/video/web conferencing (20/105, 19%) for rehabilitation monitoring [28, 38, 51, 63, 71, 72, 77, 81, 87, 88, 94, 96, 99, 105, 109, 110, 115, 118, 119, 170]. The highest use of mobile apps associated with or without a wearable was in Europe and the United Kingdom, followed by North America. Tele/video/web conferencing was used across regions, with the highest number in North America (Figure 2).

#### Validation Studies

There were 28 validation studies. Studies that validated stand-alone technologies included those to assess ROM (n=16) [24, 29, 43, 45, 48, 49, 52, 68, 73, 84, 100, 101, 111, 112, 116, 125] or gait or posture (n=10) [29, 30, 33, 53, 57, 74, 89, 97, 102, 124], and 2 studies involved technologies to monitor exercises [98, 114]. The technologies involved were either wearables (n=20) [24, 29, 30, 33, 45, 48, 49, 57, 68, 73, 84, 89, 97, 98, 100, 111, 112, 114, 116, 125], sensor-based devices (nonwearables; n=4) [53, 66, 102, 124], or inbuilt sensors available within a smartphone (n=4) [43, 52, 74, 101] (Table S4 in Multimedia Appendix 1).

In terms of study design, 9 were cross-sectional studies [33, 48, 52, 57, 84, 89, 97, 101, 116], 7 were cohort or longitudinal studies [45, 53, 68, 74, 100, 111, 125], 5 were pre-post studies [29, 30, 43, 73, 102], 1 was an uncontrolled trial [112], 1 was a randomized controlled trial (RCT) [66], and 5 were articles that described the proof of concept or development plan for the technologies [24, 49, 98, 114, 124]. The participant sample size ranged from 1 to 60. Most studies reported reliability between a standard or universal goniometer and smartphone app goniometry and the clinical evaluation of sensors to measure gait parameters (Table S4 in Multimedia Appendix 1). In 7 studies, gait was measured using sensors provided by a health care provider in a hospital setting [29, 33, 57, 74, 89, 97, 102], and 3 studies did not describe the measurement setting [30, 53, 124].

#### Clinical Evaluation Studies

**Figure 3:**
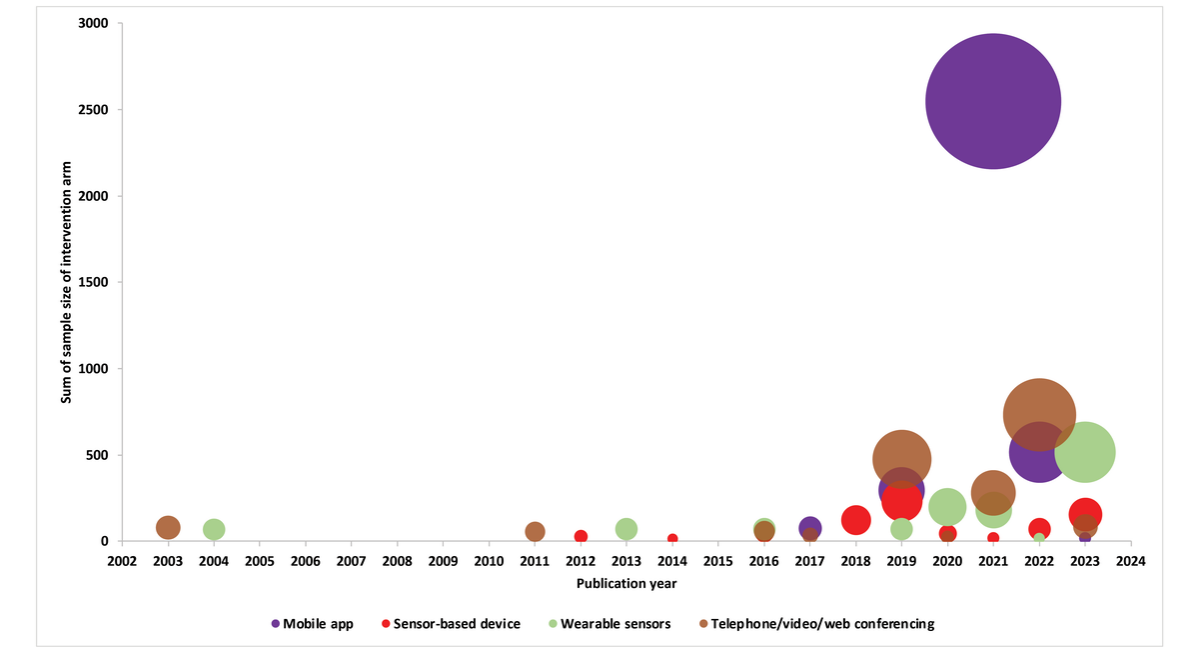
Technologies developed over the years by sample size (n=64), with the size of the bubble indicating the sample size of the intervention arm of all the studies published that particular year per technology category. Mobile app = mobile app + mobile app with inbuilt sensors; sensor-based device = sensor-based device with a mobile app + sensor-based device with a computer application; wearable sensors = wearable sensors with a mobile app + wearable sensors with a computer application + wearable sensors.

There were 64 clinical evaluation studies. The technology consisted of a mobile or computer app with a wearable device (n=18) [6, 26, 31, 32, 39, 44, 46, 50, 54, 64, 67, 69, 90, 92, 95, 106, 108, 169], a mobile or computer app with a sensor-based device (n=13) [25, 34, 35, 40, 42, 65, 70, 76, 79, 85, 86, 93, 120], only a mobile app (n=14) [36, 37, 55, 56, 62, 75, 78, 80, 83, 107, 113, 117, 123, 128], or only telephone or videoconferencing (n=19) for remote monitoring [28, 38, 51, 63, 71, 72, 77, 81, 88, 94, 96, 99, 105, 109, 110, 115, 118, 119, 170]. Of the studies that used a mobile app, 9 studies were developed only for iOS [55, 67, 69, 71, 77, 92, 106, 107, 109], 1 was an Android app [42], 7 were for both Android and iOS devices [28, 36, 56, 88, 108, 115, 117], and 21 studies did not specify the platform (Multimedia Appendix 2). A web-based clinician portal for synchronous or asynchronous remote monitoring of patients was reported by 36 studies (Table 1). The number of published studies and the intervention arm sample size (ranging from 7 to 2292), especially for those that included wearable sensors and mobile apps, steadily increased over the last 2 decades (Figure 3).

**Table 1:** Summary of application functionality (N=64).

First author, year	Web portal	Devices			Peer		App name
	Monitoring	Wearables	Sensor-based devices				
Alexander, 2023 [67]	✓	Apple Watch	—^a^	—		mymobility	
An, 2021 [110]	—	—	—	—		—	
Argent, 2019 [169]	—	IMU^b^	Avatar	—		—	
Bäcker, 2021 [25]	—	GenuSport	—	—		GenuSport	
Bade, 2020 [166]	—	In-shoe sensors	—	—		—	
Bell, 2020 [90]	✓	InterACTION IMU	—	—		—	
Bini, 2017 [71]	✓	—	—	—		Capture proof	
Blasco, 2022 [28]	—	—	—	—		WeChat app	
Campbell, 2019 [72]	✓	—	—	—		StreaMD	
Chughtai, 2018 [76]	✓	—	VERA^c^	—		VERA	
Chughtai, 2019 [75]	—	—	—	✓		PReHab	
Colomina, 2021 [31]	✓	Fitbit Flex 2	—	—		—	
Correia, 2019 [32]	✓	IMU	—	—		—	
De Berardinis, 2022 [26]	✓	Magnetic sensors with Velcro bands	—	—		kari	
Doiron-Cadrin, 2020 [77]	—	—	—	—		Reacts Lite	
Duong, 2023 [106]	✓	Fitbit, ActivPal, Goniometer Pro	—	—		—	
Eichler, 2019 [34]	✓	Kinect sensor	—	—		MainReha app	
Eisermann, 2004 [39]	✓	Accelerometers, wrist band, chest sensors	Web cams	—		—	
Farr-Wharton, 2020 [108]	✓	Garmin Vivosmart heart rate activity tracker	—	—		—	
Fung, 2012 [79]	—	—	Wii sensor balance	—		—	
Gianola, 2020 [35]	—	—	Avatar	—		—	
Gohir, 2021 [36]	✓	—	—	—		i-Beat app	
Gray, 2022 [37]	✓	—	—	—		Digital Joint School using GoWell health program	
Gunduz, 2021 [38]	—	—	—	—		—	
Hadamus, 2022 [40]	—	—	Kinetic camera	—		—	
Hardwick-Morris, 2022 [107]	✓	—	—	—		Physitrack	
Hong, 2022 [80]	—	—	—	—		Digital Musculoskeletal Surgical Care Program app	
Huang, 2017 [113]	—	—	—	—		Yishu	
Janhunen, 2023 [42]	—	—	Kinect sensor with TV and tablet	—		—	
Juhl, 2016 [44]	✓	IMU	—	—		ICURA app	
Klement, 2019 [81]	✓	—	—	—		—	
Knapp, 2021 [83]	✓	—	—	—		—	
Kramer, 2003 [99]	—	—	—	—		—	
Kuether, 2019 [85]	✓	—	VERA	—		—	
Lam, 2016 [86]	✓	IMU	—	—		ReHab system	
Lebleu, 2023 [46]	✓	Activity tracker Garmin vívofit 4	—	—		moveUP Therapy	
LeBrun, 2022 [78]	✓	—	—	—		MyChart app	
Li, 2023 [115]	—	—	—	✓		—	
Lu, 2021 [117]	—	—	—	✓		—	
McDonall, 2022 [147]	✓	—	—	—		—	
Mehta, 2020 [6]	✓	Activity tracker	—	✓		—	
Milliren, 2022 [88]	—	—	—	—		Ubicare Smart X	
Nuevo, 2023 [50]	✓	Accelerometer, gyroscope, magnetometer (DyCare)	—	—		ReHub	
Osterloh, 2023 [51]	✓	—	—	✓		YOLii	
Park, 2017 [118]	—	—	—	—		—	
Park, 2023 [119]	—	—	—	—		—	
Piqueras, 2013 [54]	✓	(WAGYRO)	Avatar	—		—	
Pournajaf, 2022 [65]	—	—	—	—		—	
Pronk, 2020 [55]	—	—	—	—		Pain coach app	
Prvu Bettger, 2019 [70]	✓	—	VERA	—		—	
Ramkumar, 2019 [92]	✓	Motion sensors	—	—		Focus ventures RPM	
Russell, 2011 [105]	—	—	—	—		—	
Scheper, 2019 [56]	✓	—	—	—		Woundcare app	
Su, 2015 [120]	—	Kinect sensor	—	—		—	
Summers, 2023 [93]	✓	—	Electro-mechanical device	—		—	
Szöts, 2016 [170]	—	—	—	—		—	
Timmers, 2019 [62]	—	—	—	—		The Patient Journey app	
Torpil, 2022 [63]	—	—	—	—		—	
Tousignant, 2011 [94]	✓	—	—	—		—	
Tripuraneni, 2021 [95]	✓	Smart watch	—	—		—	
van Dijk-Huisman, 2020 [64]	✓	MOX activity monitor	—	—		—	
Visperas, 2021 [96]	✓	—	—	—		—	
Wang, 2023 [121]	—	—	—	✓		WeChat app	
Zhang, 2021 [123]	—	—	—	✓		WeChat app	

^a^Not applicable.

^b^IMU: inertial motion unit.

^c^VERA: Virtual Exercise Rehabilitation Assistant.

Although most studies described the features and functionality of the technology to deliver the intervention, they lacked details about the technological aspects that could benefit future researchers. For example, 2 studies [31, 86] explicitly reported information on the software, programming language and tools used, or calibration procedures either along with the main study or cited the article that described the development phase. Information on conceptualization of the technology-based intervention was described in only 3 studies [37, 38, 120]. End users’ involvement was typically late during the development phase (ie, prototype stage) and involved refining the functionalities and features of technology [32, 51, 64, 65, 75-77, 80, 86, 88, 115, 118, 169] prior to deployment. Patient feedback on their needs at an early development phase was reported only by Blasco et al [28].

Clinical effectiveness was tested using an RCT design in 57.8% (37/64) of the studies [6, 25, 28, 34-36, 39, 42, 44, 50, 51, 54, 55, 62, 63, 65, 67, 70-72, 77, 79, 90, 94-96, 99, 105, 106, 108-110, 117-119, 128, 170], and the rest of the studies were either retrospective comparative cohort studies (n=3) [26, 37, 78], uncontrolled cohort studies (n=9) [46, 56, 69, 76, 81, 83, 85, 92, 169], cross-sectional studies (n=1) [86], or non-RCTs (n=14) [31, 32, 38, 40, 64, 75, 80, 88, 93, 107, 113, 115, 120, 123].

We found 13 study protocols, of which 12 were RCTs published between 2013 and 2023 [11, 41, 47, 58-60, 82, 87, 91, 103, 104, 122], for which we could not find a published report and hence were not included in this summary. User experience was measured in trials using quantitative (n=9) [32, 34, 38, 50, 55, 94, 96, 105, 123], qualitative (n=2) [61, 128], and mixed methods (n=3) [39, 90, 109] approaches.

#### Application Functionality for Rehabilitation Programs

The key functionalities of the telerehabilitation technologies extracted from 64 studies are summarized under 4 themes, namely education and enablement, monitoring progress, communication, and goal setting (Table 2).

**Table 2:** Themes of the key functionalities of the telerehabilitation technologies.

First author, year	Exercise							Monitoring progress							Functions			Communication	
	Repository	Diary	Tracker or reminder	Biofeedback	VR^a^	Feedback to patient	Pain		ROM^b^	Knee function	Physical activity	Sedentary time	Sleep	Triggers		Goal setting	Direction		Mode
																
Alexander, 2023 [67]	✓	—^c^	✓	—	—	SP^d^	—		—	✓	✓	—	—	—		—	2-way		Text, F2F^e^
An, 2021 [110]	✓	—	—	—	—	SP	—		—	—	—	—	—	—		—	2-way		Video
Argent, 2019 [169]	✓	—	✓	✓	✓	SA^f^	✓		✓	—	—	—	—	—		Exercise	2-way		F2F
Bäcker, 2021 [25]	✓	—	✓	✓	—	SA	—		—	—	—	—	—	—		Exercise	—		—
Bade, 2020 [166]	✓	—	✓	✓	—	SA	—		—	—	—	—	—	—		—	2-way		F2F
Bell, 2020 [90]	✓	—	✓	✓	—	SA, AP^g^	—		✓	—	—	—	—	—		—	2-way		Video
Bini, 2017 [71]	✓	—	—	—	—	AP	—		—	—	—	—	—	—		—	2-way		Text, video, F2F
Blasco, 2022 [28]	—	—	—	—	—	—	—		—	—	—	—	—	SC^h^		—	1-way, 2-way		Text, audio, F2F
Campbell, 2019 [72]	✓	—	✓	—	—	AP	—		—	—	—	—	—	—		—	1-way (SMS text messaging bot)		Video, text
Chughtai, 2018 [76]	✓	—	✓	✓	✓	SA, SP	✓		✓	✓	✓	—	—	—		—	2-way		Video
Chughtai, 2019 [75]	✓	✓	—	—	—	—	✓		—	—	—	—	—	—		—	—		—
Colomina, 2021 [31]	—	—	✓	—	—	SA, AP	✓		—	—	✓	✓	✓	SC		Exercise	2-way		Text
Correia, 2019 [32]	✓	—	✓	✓	—	SA, AP	—		✓	—	—	—	—	—		—	2-way		Audio, F2F
De Berardinis, 2022 [26]	✓	—	✓	✓	—	SA	—		—	—	—	—	—	SC		Exercise	2-way		F2F
Doiron-Cadrin, 2019 [77]	✓	—	✓	—	—	SP	—		—	—	—	—	—	—		—	2-way		Video
Duong, 2023 [106]	✓	—	✓	—	—	AA^i^, AP	✓		✓	✓	✓	✓	✓	SC		Activity	1-way, 2-way		Text, video
Eichler, 2019 [34]	✓	—	✓	✓	✓	SA, AP	—		✓	—	—	—	—	—		Exercise	1-way, 2-way		Audio, video, text, F2F
Eisermann, 2004 [39]	✓	—	—	✓	—	SA, AP	✓		—	—	✓	—	—	—		—	2-way		Text
Farr-Wharton, 2020 [108]	✓	—	✓	—	—	AA, AP	✓		✓	—	✓	—	✓	DS^j^		Function	1-way		Text, audio
Fung, 2012 [79]	✓	—	✓	✓	✓	SA	—		—	—	—	—	—	—		Lower extremity function	2-way		F2F
Gianola, 2020 [35]	✓	—	✓	✓	✓	SA	—		—	—	—	—	—	—		Exercise	—		—
Gohir, 2021 [36]	✓	—	✓	—	—	AA, AP	—		—	—	—	—	—	—		Exercise	1-way, 2-way		Text, audio (tele)
Gray, 2022 [37]	✓	—	—	—	—	SP	—		—	—	—	—	—	—		—	1-way, 2-way		Text
Gunduz, 2021 [38]	✓	—	—	—	—	—	—		—	—	—	—	—	—		—	—		—
Hadamus, 2022 [40]	✓	—	—	✓	✓	SA, SP	—		—	—	—	—	—	—		Exercise	2-way		F2F
Hardwick-Morris, 2022 [107]	✓	✓	—	—	—	SP	✓		—	—	—	—	—	SC		—	2-way		Video, text
Hong, 2022 [80]	✓	—	—	—	—	SP	—		—	—	—	—	—	—		Recovery goals	2-way		Video
Huang, 2017 [113]	✓	—	—	—	—	—	—		—	—	—	—	—	—		—	—		—
Janhunen, 2023 [42]	✓	✓	—	✓	✓	SA	—		—	—	—	—	—	—		Exercise	—		—
Juhl, 2016 [44]	✓	—	—	✓	—	SP	—		—	—	—	—	—	SC		—	2-way		Unclear
Klement, 2019 [81]	✓	—	✓	—	—	—	—		—	—	—	—	—	—		—	1-way, 2-way		Text, videos, F2F
Knapp, 2021 [83]	✓	—	✓	—	—	—	—		—	—	—	—	—	NU^k^		—	—		—
Kramer, 2003 [99]	—	—	✓	—	—	—	—		—	—	—	—	—	SC		—	2-way		Audio
Kuether, 2019 [85]	✓	—	✓	✓	✓	SA, SP	—		—	✓	✓	—	—	—		—	2-way		F2F, video
Lam, 2016 [86]	✓	—	✓	✓	✓	SA, SP	—		✓	—	—	—	—	—		ROM, strength	—		—
Lebleu, 2023 [46]	✓	✓	—	✓	—	SA, AP	✓		✓	✓	✓	—	—	DS		—	2-way		Text
LeBrun, 2022 [78]	✓	—	—	—	—	SP	—		—	—	—	—	—	SC		—	2-way		Audio, video
Li, 2023 [115]	✓	—	✓	—	—	SP	—		—	—	—	—	—	—		—	2-way		Video, text
Lu, 2021 [117]	✓	—	—	—	—	SP	—		—	—	—	—	—	SC		—	2-way		Video
McDonall, 2022 [147]	✓	—	—	—	—	—	—		—	—	—	—	—	—		Pain management, knee function, avoiding complications	—		—
Mehta, 2020 [6]	—	—	✓	—	—	AA	✓		—	—	✓	—	—	DS, NU		Activity	1-way, 2-way		Text, F2F
Milliren, 2022 [88]	—	—	—	—	—	—	—		—	—	—	—	—	—		Discharge goal	1-way		Text (automatic)
Nuevo, 2023 [50]	✓	—	✓	✓	—	SA	✓		✓	—	—	—	—	DS, NU		—	2-way		Video, text
Osterloh, 2023 [51]	✓	—	—	—	—	SP	—		—	—	—	—	—	SC		—	2-way		Video
Park, 2017 [118]	—	—	✓	—	—	—	—		—	—	—	—	—	SC		—	1-way, 2-way		Text, audio (tele),
Park, 2023 [119]	—	—	—	—	—	SP	—		—	—	—	—	—	SC		—	2-way		Audio calls
Piqueras, 2013 [54]	✓	—	✓	✓	✓	SA, AP	—		✓	—	—	—	—	—		—	2-way		Audio (tele)
Pournajaf, 2022 [65]	✓	—	—	✓	✓	SA	—		—	—	—	—	—	VR-based balance board		Exercise	✓		Exercise
Pronk, 2020 [55]	✓	—	—	—	—	Unclear	✓		—	—	—	—	—	—		—	—		—
Prvu Bettger, 2019 [70]	✓	—	—	✓	✓	SA, SP, AP	—		—	✓	✓	✓	—	—		Exercise	2-way		Video, F2F
Ramkumar, 2019 [92]	✓	—	✓	✓	✓	SA	✓		✓	—	✓	—	—	DS		Exercise	1-way		Text
Russell, 2011 [105]	✓	✓	—	—	—	SP	—		✓	—	✓	—	—	SC		Unclear	2-way		Video
Scheper, 2019 [56]	—	—	—	—	—	—	✓		—	—	—	—	—	DS		—	1-way		Text
Su, 2015 [120]	✓	—	—	✓	✓	SA	—		—	—	—	—	—	—		Exercise	—		—
Summers, 2023 [93]	✓	—	✓	✓	—	SA, SP	✓		✓	✓	✓	—	—	DS		—	2-way		Video
Szöts, 2016 [170]	—	—	—	—	—	—	—		—	—	—	—	—	—		—	2-way		Audio (tele)
Timmers, 2019 [62]	✓	—	—	—	—	—	✓		—	—	—	—	—	—		—	1-way		Audio, video, text
Torpil, 2022 [63]	—	—	—	—	—	—	—		—	—	—	—	—	SC		Occupation related	2-way		Video
Tousignant, 2011 [94]	—	—	—	—	—	SP	—		—	—	—	—	—	SC		—	2-way		Video
Tripuraneni, 2021 [95]	✓	—	✓	—	—	AA	—		—	—	✓	✓	—	—		—	1-way		Text
van Dijk-Huisman, 2020 [64]	✓	—	✓	✓	—	SA, AP	—		—	—	✓	—	—	SC		—	2-way		Video
Visperas, 2021 [96]	✓	—	—	—	—	AP	✓		—	✓	—	—	—	DS, SC		—	1-way, 2-way		Text, audio (telephone)
Wang, 2023 [121]	✓	✓	✓	—	—	AP	—		—	—	—	—	—	—		Task	2-way		Text
Zhang, 2021 [123]	✓	—	—	—	—	—	—		—	—	—	—	—	—		—	2-way		Audio, text, video

^a^VR: virtual reality.

^b^ROM: range of motion.

^c^Not applicable.

^d^SP: synchronous from physiotherapist.

^e^F2F: face to face.

^f^SA: synchronous from app.

^g^AP: asynchronous from physiotherapist.

^h^SC: scheduled call.

^i^AA: asynchronous from app.

^j^DS: danger signs.

^k^NU: non-use.

#### Education and Enablement

An exercise repository in the form of videos, text, or infographics was one of the main features in the studies (n=53), of which only 20 studies described the list of exercises (Table S5 in Multimedia Appendix 1). Education for patients was part of the rehabilitation program in 17 studies. Table S6 in Multimedia Appendix 1 lists the topic areas covered in the education materials. Regarding exercise, 6 studies reported using an e-diary for maintaining an exercise log, 11 studies reported using reminders to perform exercises, and 13 studies reported using a tracker for exercise adherence (Multimedia Appendix 2). Feedback on the appropriateness of exercise performance was synchronous (biofeedback or virtual reality) from the app (n=19), directly from the health care provider via a video call with the patients (patient performing exercise live, measurement of ROM during video call, transmission of virtual avatar data to health care provider; n=14), or provided via both (n=6; Table 2). Feedback to the patient, which was either in the form of push notifications or a progress summary, was asynchronous from the app using automated programs in 2 studies. Asynchronous feedback from a health care provider in the form of instructions, messages, or an exercise regimen was reported in 13 studies. Feedback via both the app and a health care provider was provided in 3 studies (Table 2). Only 7 studies [6, 51, 75, 115, 117, 123, 128] had an option for peer support for patients.

#### Measuring Progress

Measurement of patient-reported outcomes such as pain (n=19) was an inbuilt feature in the app. Changes in knee function and activity were monitored directly via wearables or captured using patient-reported outcome measures. These included ROM in 15 studies, knee function in 8 studies, physical activity in 20 studies, sedentary behavior in 5 studies, and sleep in 4 studies. Automatic alerts were provided to the health care provider for any danger signs such as knee pain, wound health, opioid consumption, function, ROM, number of steps, exercise adherence, and any negative response to questions after entering the postoperative follow-up in 9 studies; for non-use of the technology by patients in 4 studies; and for scheduled consultations in 18 studies (Table 2).

#### Communication

Mobile app–enabled 1-way communication included push messages, notifications, reminders, patients’ replies to inbuilt questions in the app, information sent to the patient by the health care team, and an SMS text messaging bot (n=10). Two-way communication, either via an app or in face-to-face visits, was reported in 41 studies. In addition, 11 studies reported a combination of both 1 and 2-way communication, and 1 study did not provide sufficient information about communication. Electronic communication was delivered in the form of text, audio or video messages, and direct communication (Table 2).

#### Goal Setting

Goal setting for exercises, activity, pain management, knee function, ROM, muscle strength, rehabilitation, and discharge as part of the rehabilitation program was reported in 23 studies. The goals were set by either the health care provider or the patient (Table 2).

### End Users’ Perceptions

Of the 38 studies that reported user perspectives, 2 focused on the perspectives of health care providers, 27 focused on the perspectives of patients and caregivers, and 9 focused on the perspectives of both groups (health care providers and patients and caregivers). The approach for data collection was quantitative (n=23), qualitative (n=9), or mixed methods (n=6). The sample size ranged from 2 to 200 health care providers and from 5 to 2292 patients (Tables S7 and S8 in Multimedia Appendix 1).

Commonly used quantitative questionnaires to assess satisfaction were the System Usability Scale [129] and the net promoter score [130]. To ratify the experience with telerehabilitation, the Telemedicine Perception Questionnaire was used [131]. Acceptability and usability were assessed using the acceptance of information technology questionnaire [132] and the Telemedicine Usability Questionnaire [133]. Some studies used bespoke questionnaires to report user experience and satisfaction [32, 39, 61, 90, 94, 105, 109, 134-146].

**Figure 4:**
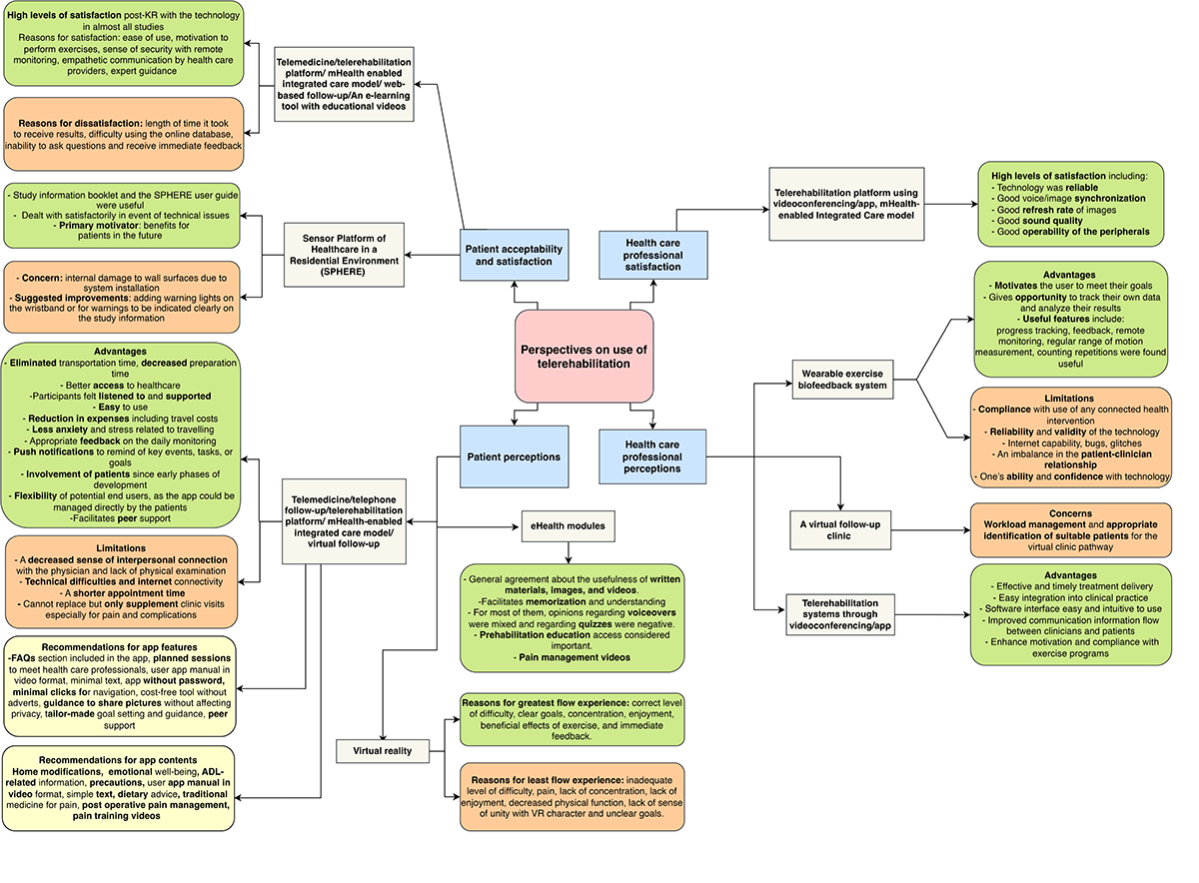
Perceptions of patients and health care providers about the technology used. ADL: activities of daily living; FAQs: frequently asked questions; KR: knee replacement; mHealth: mobile health; VR: virtual reality.

Overall, health care providers perceived telerehabilitation and the use of technology such as biosensors as a way of improving efficiency in providing care [146], patient adherence to exercises [39, 136, 146], patient-physician communication [136], and case management [137, 146]. The main factors associated with user satisfaction with e-consultations were reliable technology, good voice or image synchronization, the refresh rate of images, sound quality, and operability of the peripherals [94, 96, 138, 139]. The key factors they perceived would influence use and uptake of technology were decreased workload (rather than increased) [140], reliability of measurements aided by technology [146], ability to measure functional outcomes objectively [141], clearer criteria when choosing appropriate patients to be enrolled in the program [140], self-efficacy in the use of technology [94, 138, 146], and ease of reporting and tracking of patient data [90]. Patients and health care providers felt e-learning modules, push notifications, and appropriate feedback from sensors and virtual reality improved self-management [138, 142-144] (Figure 4).

Patient satisfaction levels were reported when teleconsultation was provided via a computer, smartphone, or tablet [34, 39, 55, 56, 80, 92, 105, 121, 123, 134, 135, 145, 147-149]; telephone [61], videoconferencing [38, 77, 94, 105, 139, 141, 150-152], a web-based system [32, 50, 90, 96, 140, 153], and an mHealth-enabled integrated care model [46, 88, 138]. Patients were satisfied with telemonitoring due to improved access to services, continued support after discharge from hospital, ability for self-management, reduced need for clinic visits, reduction in cost and travel time, ability of health care providers to provide personalized care [32, 61, 94, 121, 136, 138, 140, 141, 145, 153-155], ease of use [34, 50, 55, 56, 92, 105, 135, 138, 147, 148], motivation to perform exercises [134, 135], sense of security with remote monitoring [134, 155], and empathetic communication by a health care provider [121, 135, 136, 145, 152, 155]. The reasons for dissatisfaction were lack of an in-person examination, shorter appointment times, delay in receiving reports (eg, x-ray), and an inability to transfer pictures from one technology to another [140, 145, 149, 153]. Patients provided suggestions for the app functionalities to improve the ease of use such as minimal clicks, an instructional video for app navigation, and restriction of commercial advertisements [149]. Home modifications [149], emotional well-being, information related to activities of daily living in simple text, dietary advice, frequently asked questions, and use of traditional medicine for postoperative pain management were a few of the suggestions for app content [121].

Patients were generally satisfied with the telerehabilitation program and were ready to recommend it to others [39, 80, 85, 96, 121, 135, 151]. The use of technology for rehabilitation was influenced by computer literacy [141, 150]. However, interruption of virtual physiotherapy sessions due to poor internet issues [139] was not commonly reported (Figure 4).

## Discussion

### Principal Findings

This scoping review summarized the extent, user perceptions, range, and nature of technologies used to support rehabilitation following knee arthroplasty. All studies reported in this review were from upper and middle-to-upper–income countries, with a steep increase in studies in the last decade. The technologies focused on enabling patients to remember prescribed exercises as well as be able to perform them appropriately by providing synchronous and asynchronous feedback via biosensors or virtual reality. Motivation and support during recovery via technology-enabled 1-way or 2-way communication gave patients access to health care providers. Self-management and monitoring of progress were dependent on active input using e-diaries by patients or passive input through wearables. In the context where these technologies were evaluated, end users were satisfied and found remote monitoring to be acceptable for routine use.

The last decade has seen an exponential increase in the number of arthroplasties worldwide [156]; however, a corresponding increase in technological solutions to facilitate remote monitoring is nonexistent in resource-limited settings such as LMICs where the need for monitoring and a continuum of care may be higher due to lower literacy levels and lack of access to rehabilitation clinics. Research on this topic that can inform clinical practice is nonexistent in the LMIC context. Despite a high penetration of the smartphone market [157] in LMICs, a higher initial investment to develop the technology, especially in the health care sector [158], or a lack of publication of such efforts could be reasons. In LMICs, there is an increasing trend of lower limb joint replacement procedures [156]. High out-of-pocket expenditures incurred due to home visits by physiotherapists or clinic visits by patients [159] dictate the need for a cost-effective and feasible technology-based strategy to fit the context while using lessons learned from available research.

There is unequivocal evidence that there is a need for physical and psychological support from professionals during the recovery period for pain management, adherence to exercises, and modifications to therapy planning based on one’s progress [3, 160, 161]. The apps were either focused on a single function (such as communication or knowledge transfer) or were multifunctional. They were generally received well by end users; however, the usability and acceptability of these applications or remote monitoring modalities cannot be extrapolated to low health literacy and tech literacy settings. The challenges we expect with using remote monitoring in the LMIC context could be inequitable smartphone access or tech literacy, internet speed, affordability of wearables, the burden to the health system if these needs are provided free of cost, and the need for educational content in multiple languages in countries with a non-native English-speaking, multilingual population such as in India [162].

### Implications for Future Research

mHealth interventions have the potential to expand the reach and effectiveness of health support by facilitating behavior change. However, to ensure these “digital behavior health interventions” effectively engage users and are effective, both microengagement (the mHealth interface) and macroengagement (evidence-based behavior change techniques) are essential [163, 164]. However, we found only a handful of studies that reported user involvement during the development stage [28, 32, 51, 58, 64, 65, 75-77, 80, 86, 88, 115, 118, 169]. Studies rarely provided an adequate explanation of the theoretical behavioral framework behind the technology-based interventions [165].

Since the context and technologies are so varied, any new applications that are developed, especially in the LMIC context, should undertake formative research with end users to understand their needs, understand their preferences, and study the local digital regulatory requirements before investing time and effort. Feasibility and pilot testing by a multidisciplinary team should be crucial steps before a full-scale evaluation [69, 166], and embedding end users’ involvement and documenting their experiences at every stage are vital to refining future interventions [164]. Further, the rehabilitation protocols should map the application features with the desired function [167, 168], and this should be confirmed by means of a process evaluation embedded within the clinical evaluation to inform the mechanism of the impact in a real-life setting [147].

### Limitations

This review needs to be interpreted in light of the following limitations. This scoping review focused only on technology interventions for post-knee replacement rehabilitation and hence cannot be extrapolated to other orthopedic procedures. We did not include articles for which the full text was not available. Further, incomplete reporting on the features and functions of the technology is possible and may have affected our qualitative summary and conclusion.

We did not perform a consultation phase as per the guidelines [20], and the research question was formulated upon discussion between the researchers of the scoping review team, physiotherapists, and clinicians. We limited our search from 2001 onward; however, since knee arthroplasty and mHealth came into practice in the last 2 decades, this restriction in the search may not have an implication for our review findings.

### Conclusion

Several technologies have been identified to promote adherence, increase self-efficacy, enhance self-management, and support remote monitoring. However, all the available technologies have been developed and used in developed countries. The need for remote monitoring is compelling in resource-limited countries where knee arthroplasty is on the rise. However, irrespective of the context, it is important to involve a multidisciplinary team and include users’ perspectives during the development stage.

### What Was Already Known About the Topic

Computer and mobile technologies to support rehabilitation following knee arthroplasty are in wide use. Telerehabilitation and remote monitoring are as effective and safe as clinic-based rehabilitation programs. They reduce out-of-pocket expenditure or health cost expenditure by reducing the time to discharge following surgery and the number of clinic visits after discharge.

### What This Study Adds

This study provides a map of the types of technology and the functionality of mobile and computer-based multifunction applications. We summarized end users’ perceptions and reasons for satisfaction or dissatisfaction with available technology. The findings reflect the lack of research and readily available technologies for LMICs.

## Appendix

Multimedia Appendix 1 Supplementary information on search strategy, included and excluded studies.

Multimedia Appendix 2 Raw data extraction file for rehabilitation program studies.

Multimedia Appendix 3 PRISMA-ScR checklist.

## Abbreviations

CENTRAL: Cochrane Central Register of Controlled Trials
JBI: Joanna Briggs Institute
LMIC: low and middle-income countries
mHealth: mobile health
PRISMA-ScR: Preferred Reporting Items for Systematic Review and Meta-Analyses Extension for Scoping Reviews
RCT: randomized controlled trial
ROM: range of motion
