# Supplementary material for: Mobile and Computer-Based Applications for Rehabilitation Monitoring and Self-Management After Knee Arthroplasty: Scoping Review
Source: JMIR Mhealth Uhealth. 2024 Jan 26;12:e47843. doi: 10.2196/47843 (PMC10858429; doi:10.2196/47843)
Supplement: Multimedia Appendix 1 [file mhealth_v12i1e47843_app1.docx]

**Mobile and computer-based applications for rehabilitation monitoring and self-management after knee arthroplasty: A scoping review**

[Table S1: Framework for key search words 2](#_Toc153483496)

[Table S2 Search results 2](#_Toc153483497)

[Table S3 List of excluded articles (n=142) 6](#_Toc153483498)

[Table S4 Study characterstics of included studies (n=105) 10](#_Toc153483499)

[Table S5 List of studies with exercise as a rehabilitation intervention component (type of exercises delivered via technology) 23](#_Toc153483500)

[Table S6 List of studies that have education as a component of rehabilitation provided via any mode 29](#_Toc153483501)

[Table S7 Healthcare providers perspective on use of technology/telerehabilitation/mobile application for rehabilitation monitoring (N=11) 31](#_Toc153483502)

[Table S8 Patients and caregiver perspective on use of technology/telerehabilitation/mobile application for rehabilitation monitoring (N=36) 34](#_Toc153483503)

**Search Strategy**

Table S1: Framework for key search words

| **Concept** | **Knee arthroplasty** | **AND** | **Telemedicine** |
| --- | --- | --- | --- |
| **Controlled vocabulary** | "Arthroplasty, Replacement, Knee"[Mesh] | AND | "Telerehabilitation"[Mesh]  "Telemedicine"[Mesh]  "Remote Sensing Technology"[Mesh]  "Virtual Reality"[Mesh]  "Mobile Applications"[Mesh]  "Cell Phone"[Mesh]  "Internet-Based Intervention"[Mesh]  "Digital Technology"[Mesh]  "Wearable Electronic Devices"[Mesh]  "Videoconferencing"[Mesh] |
| **Free text terms/Synonyms** | Knee replacement  Knee arthroplasty  Knee implant  Knee endoprosthesis  Knee prosthesis | AND | Telerehabilitation  Tele rehabilitation  Tele-rehabilitation  Telemedicine  Telehealth  tele-monitoring  “Remote monitoring”  “Remote rehabilitation”  “remote tracking”  “virtual rehabilitation”  “virtual reality”  “Mobile application”  “Mobile app”  “mobile devices”  mHealth  eHealth  web-based  internet-based  computer-based  computer application  “computer software”  technology  gamification  game-based  sensor  videoconferencing |

Table S2 Search results

| Embase Classic+Embase <1947 to 2021 October 13>  1 exp Arthroplasty, Replacement, Knee/ 18362  2 (knee adj3 (replac* or arthroplast* or implant* or endoprosthe* or prosthe*)).mp. 62463  3 1 or 2 62463  4 exp telehealth/ 63802  5 exp telemedicine/ 54407  6 exp telerehabilitation/ 1453  7 exp telemonitoring/ 4060  8 exp remote sensing/ 13275  9 exp virtual reality/ 20519  10 exp mobile application/ 17435  11 exp mobile phone/ 36301  12 exp internet/ 118245  13 exp software/ 233887  14 exp technology/ 247745  15 exp game/ 8115  16 exp wearable sensor/ 728  17 exp motion sensor/ 159  18 exp videoconferencing/ 6070  19 ((tele* or mobile or phone or online or internet or digital) and (rehab* or care or medicine or health)).mp. 400865  20 (telehealth or telemedicine or telerehab* or mhealth).mp. 57372  21 (wearable* or ((motion or movement or activity or mobility) adj3 (sens* or monitor* or track*))).mp. 85343  22 ((virtual or video or electronic*) and (gam* or learn* or educat* or conferenc*)).mp. 172989  23 4 or 5 or 6 or 7 or 8 or 9 or 10 or 11 or 12 or 13 or 14 or 15 or 16 or 17 or 18 698502  24 19 or 20 or 21 or 22 637409  25 23 or 24 1159465  26 3 and 25 2589 |
| --- |
| Ovid MEDLINE(R) and In-Process, In-Data-Review & Other Non-Indexed Citations and Daily <1946 to October 13, 2021>  1 exp Arthroplasty, Replacement, Knee/ 27457  2 (knee adj3 (replac* or arthroplast* or implant* or endoprosthe* or prosthe*)).mp. 43878  3 1 or 2 43878  4 exp telehealth/ 37101  5 exp telemedicine/ 37101  6 exp telerehabilitation/ 646  7 exp telemonitoring/ 0  8 exp remote sensing/ 0  9 exp virtual reality/ 3426  10 exp mobile application/ 8731  11 exp mobile phone/ 18583  12 exp internet/ 87945  13 exp software/ 170546  14 exp technology/ 456424  15 exp game/ 0  16 exp wearable sensor/ 0  17 exp motion sensor/ 0  18 exp videoconferencing/ 2460  19 ((tele* or mobile or phone or online or internet or digital) and (rehab* or care or medicine or health)).mp. 248434  20 (telehealth or telemedicine or telerehab* or mhealth).mp. 44010  21 (wearable* or ((motion or movement or activity or mobility) adj3 (sens* or monitor* or track*))).mp. 66788  22 ((virtual or video or electronic*) and (gam* or learn* or educat* or conferenc*)).mp. 78322  23 4 or 5 or 6 or 7 or 8 or 9 or 10 or 11 or 12 or 13 or 14 or 15 or 16 or 17 or 18 720661  24 19 or 20 or 21 or 22 385334  25 23 or 24 992997  26 3 and 25 1697 |
| Combined (MEDLINE and Embase)  Embase Classic+Embase <1947 to 2021 October 13>  Ovid MEDLINE(R) and In-Process, In-Data-Review & Other Non-Indexed Citations and Daily <1946 to October 13, 2021>  1 exp Arthroplasty, Replacement, Knee/ 45819  2 (knee adj3 (replac* or arthroplast* or implant* or endoprosthe* or prosthe*)).mp. 106341  3 1 or 2 106341  4 exp telehealth/ 100903  5 exp telemedicine/ 91508  6 exp telerehabilitation/ 2099  7 exp telemonitoring/ 4060  8 exp remote sensing/ 13275  9 exp virtual reality/ 23945  10 exp mobile application/ 26166  11 exp mobile phone/ 54884  12 exp internet/ 206190  13 exp software/ 404433  14 exp technology/ 704169  15 exp game/ 8115  16 exp wearable sensor/ 728  17 exp motion sensor/ 159  18 exp videoconferencing/ 8530  19 ((tele* or mobile or phone or online or internet or digital) and (rehab* or care or medicine or health)).mp. 649299  20 (telehealth or telemedicine or telerehab* or mhealth).mp. 101382  21 (wearable* or ((motion or movement or activity or mobility) adj3 (sens* or monitor* or track*))).mp. 152131  22 ((virtual or video or electronic*) and (gam* or learn* or educat* or conferenc*)).mp. 251311  23 4 or 5 or 6 or 7 or 8 or 9 or 10 or 11 or 12 or 13 or 14 or 15 or 16 or 17 or 18 1419163  24 19 or 20 or 21 or 22 1022743  25 23 or 24 2152462  26 3 and 25 4286  27 remove duplicates from 26 3561  28 limit 27 to english language 3375  29 limit 28 to human 3136  30 limit 29 to last 20 years 3025 |
| APA PsycInfo <1806 to October Week 1 2021>  1 (knee adj3 (replac* or arthroplast* or implant* or endoprosthe* or prosthe*)).mp. 591  2 exp Rehabilitation/ 51348  3 exp Telemedicine/ 10172  4 exp Telerehabilitation/ 165  5 telemonitoring.mp. 196  6 ((tele* or mobile or phone or online or internet or digital) and (rehab* or care or medicine or health)).mp. 70379  7 (telehealth or telemedicine or telerehab* or mhealth).mp. 9414  8 (wearable* or ((motion or movement or activity or mobility) adj3 (sens* or monitor* or track*))).mp. 13361  9 exp Mobile Applications/ 1385  10 exp Digital Technology/ or exp Technology/ or exp Mobile Technology/ 239834  11 ((virtual or video or electronic*) and (gam* or learn* or educat* or conferenc*)).mp. 43930  12 exp Virtual Reality/ 9844  13 exp Mobile Phones/ 6141  14 exp Internet/ 30368  15 exp Computer Software/ 15076  16 exp Games/ 22728  17 sensor.mp. 4335  18 2 or 3 or 4 or 5 or 6 or 7 or 8 or 9 or 10 or 11 or 12 or 13 or 14 or 15 or 16 or 17 382970  19 1 and 18 140 |
| Cochrane Central  earch Name: TKR AND Rehab  Last Saved: 21/10/2021 14:39:03  Comment: 21Oct  ID Search  #1 "knee replacement"  #2 "Knee arthroplasty"  #3 ("knee replacement arthroplasty"):ti,ab,kw  #4 ("knee replacement"):ti,ab,kw  #5 ("rehabilitation"):ti,ab,kw  #6 MeSH descriptor: [Mobile Applications] explode all trees  #7 (tele rehabilitation):ti,ab,kw  #8 MeSH descriptor: [Telerehabilitation] explode all trees  #9 MeSH descriptor: [Telemedicine] explode all trees  #10 (telehealth):ti,ab,kw  #11 MeSH descriptor: [Internet] explode all trees  #12 tele OR mobile OR Phone OR online OR Internet OR digital  #13 rehab* OR care OR medicine OR health  #14 #12 AND #13  #15 wearable OR motion  #16 sensor  #17 #15 AND #16  #18 #5 OR #6 OR #7 OR #8 OR #9 OR #10 OR #11 OR #14 OR #17  #19 #1 OR #2 OR #3 OR #4  #20 #18 AND #19 with Publication Year from 2001 to 2021, in Trials |
| Embase Classic+Embase <1947 to 2023 August 02> Ovid MEDLINE(R) and In-Process, In-Data-Review & Other Non-Indexed Citations and Daily <1946 to August 02, 2023>  # Query Results from 3 Aug 2023  1 exp Arthroplasty, Replacement, Knee/ 56,342  2 (knee adj3 (replac* or arthroplast* or implant* or endoprosthe* or prosthe*)).mp. 121,527  3 1 or 2 121,527  4 exp telehealth/ 131,604  5 exp telemedicine/ 116,077  6 exp telerehabilitation/ 3,552  7 exp telemonitoring/ 5,629  8 exp remote sensing/ 17,620  9 exp virtual reality/ 32,000  10 exp mobile application/ 36,768  11 exp mobile phone/ 69,577  12 exp internet/ 226,835  13 exp software/ 543,342  14 exp technology/ 768,864  15 exp game/ 9,344  16 exp wearable sensor/ 2,583  17 exp videoconferencing/ 11,620  18 exp motion sensor/ 575  19 ((tele* or mobile or phone or online or internet or digital) and (rehab* or care or medicine or health)).mp. 828,790  20 (telehealth or telemedicine or telerehab* or mhealth).mp. 138,716  21 (wearable* or ((motion or movement or activity or mobility) adj3 (sens* or monitor* or track*))).mp. 186,011  22 ((virtual or video or electronic*) and (gam* or learn* or educat* or conferenc*)).mp. 341,137  23 4 or 5 or 6 or 7 or 8 or 9 or 10 or 11 or 12 or 13 or 14 or 15 or 16 or 17 or 18 1,686,050  24 19 or 20 or 21 or 22 1,309,738  25 23 or 24 2,635,703  26 3 and 25 5,592  27 limit 26 to yr="2021 - 2023" 1,722  28 remove duplicates from 27 1,414  exp Arthroplasty, Replacement, Knee/ (knee adj3 (replac* or arthroplast* or implant* or endoprosthe* or prosthe*)).mp. 1 or 2 exp telehealth/ exp telemedicine/ exp telerehabilitation/ exp telemonitoring/ exp remote sensing/ exp virtual reality/ exp mobile application/ exp mobile phone/ exp internet/ exp software/ exp technology/ exp game/ exp wearable sensor/ exp videoconferencing/ exp motion sensor/ ((tele* or mobile or phone or online or internet or digital) and (rehab* or care or medicine or health)).mp. (telehealth or telemedicine or telerehab* or mhealth).mp. (wearable* or ((motion or movement or activity or mobility) adj3 (sens* or monitor* or track*))).mp. ((virtual or video or electronic*) and (gam* or learn* or educat* or conferenc*)).mp. 4 or 5 or 6 or 7 or 8 or 9 or 10 or 11 or 12 or 13 or 14 or 15 or 16 or 17 or 18 19 or 20 or 21 or 22 23 or 24 3 and 25 limit 26 to yr="2021 - 2023" remove duplicates from 27 |

Table S3 List of excluded articles (n=142)

| **Author name** | **Reason for exclusion** |
| --- | --- |
| Abdeen A, 2022[1] | Irrelevant outcome |
| Agostini M, 2015[2] | Review |
| Ahmad NA, 2020 [3] | Review |
| Aksoy CC, 2017[4] | Review |
| Alexandre DJA, 2021[5] | Review |
| Allsop S, 2019[6] | Unsuitable time |
| Al-Rub ZA, 2014[7] | Perspective |
| Anthony CA, 2022[8] | Irrelevant outcome |
| Azhari A, 2020[9] | Perspective |
| Bahadori S, 2020[10] | Review |
| Baker K, 2020[11] | No mobile application |
| Baxter SN, 2023[12] | Irrelevant outcome |
| Bell KM, 2017 [13] | Others, feasibility of system for monitoring motion repetitition counters |
| Bentley J, 2020 [14] | Irrelevant outcome |
| Berton A, 2020 [15] | Review |
| Bini SA, 2019 [16] | Irrelevant outcome |
| Blasco J, 2021 [17] | Review |
| Boekesteijn R, 2021[18] | Irrelevant outcome |
| Bogue E, 2017 [19] | Irrelevant outcome |
| Browne JA, 2021 [20] | Perspective |
| Bruyere O, 2017 [21] | No mobile application |
| Buvanendran A, 2021 [22] | No mobile application |
| Byra J, 2020 [23] | Review |
| Cankaya D, 2021[24] | Irrelevant outcome |
| Chaudhry H, 2021 [25] | Review |
| Chen M, 2016 [26] | No mobile application |
| Chughtai M, 2017 [27] | Full text/Abstract not available |
| Coenen P, 2020 [28] | No mobile application |
| Cooper RA, 2019 [29] | Perspective |
| Dahlberg LE, 2023[30] | Full text/Abstract not available |
| De Faoite D, 2018 [31] | Review |
| Denecke K, 2018 [32] | Others, concept for managing cross-sectoral clinical pathway |
| Duong V, 2022[33] | Irrelevant outcome |
| El Ashmawy A-AH, 2020 [34] | Others, Virtual follow-up clinics |
| Fabres Martin C, 2023[35] | Others, Follow-up Clinic |
| Fennema MC, 2019 [36] | No mobile application |
| Ferguson-Pell M, 2021 [37] | No mobile application |
| Ferrara PE, 2020 [38] | Review |
| Ferriero G, 2014 [39] | Others, letter to editor |
| Freiman S, 2021 [40] | No mobile application |
| Fuchs L, 2022[41] | Irrelevant outcome |
| Fung V, 2011 [42] | No mobile application |
| Fusco F, 2015 [43] | Economic evaluation |
| Fusco F, 2014 [44] | Economic evaluation |
| Fusco F, 2015 [45] | Perspective |
| Gakhar H, 2013 [46] | No mobile application |
| Gilbert AW, 2018[47] | Review |
| Gollish JD, 2019 [48] | Full text/Abstract not available |
| Gonzalez Ruiz C, 2019 [49] | Full text/Abstract not available |
| Grant S, 2018 [50] | No mobile application |
| Grant S, 2018 [51] | No mobile application |
| Gumaa M, 2019 [52] | Review |
| Gwam CU, 2019 [53] | Irrelevant outcome |
| Hadley C, 2019 [54] | Irrelevant population |
| Hallfors E, 2018 [55] | No mobile application |
| Han S-L, 2016 [56] | Others, feasibility of inertial measurement units (IMU) and an ankle foot orthosis for clinical usage |
| Naeemabadi M SJ, 2020 [57] | Others, participatory design to explore patients needs for telerehabilitation program |
| Hardt S, 2018 [58] | Unsuitable time |
| Ho A, 2010 [59] | Others, duplicate with other included study |
| Holmes M, 2018 [60] | No mobile application |
| Huang S, 2020[61] | Others, Not rehabilitation protocol |
| Jansson M, 2020[62] | Review |
| Jansson J, 2022[63] | Review |
| Jayakumar P, 2021 [64] | Irrelevant outcome |
| Jetanalin Pim, 2012[65] | Irrelevant outcome |
| Jiang S, 2018 [66] | Review |
| Kang K, 2018 [67] | Others, incomplete information |
| Karasavvidis T, 2020 [68] | Review |
| Kim K, 2016 [69] | Full text/Abstract not available |
| Kocak UZ, 2021 [70] | Irrelevant outcome |
| Koutras C, 2015 [71] | Review |
| Krumsvik OA, 2017 [72] | Irrelevant outcome |
| Kuan WYH, 2020 [73] | Not enough information |
| Kurtz SM, 2022[74] | Full text/Abstract not available |
| Kwasnicki RM, 2015 [75] | Others, method of assessing peri-operative mobility, used to supplement surgical decision-making |
| Laursen M, 2017 [76] | Irrelevant population |
| Leal-Blanquet J, 2013 [77] | No mobile application |
| Li P, 2021[78] | Irrelevant outcome |
| Lin H-J 2019[79] | Review |
| Linedale EC, 2023[80] | Irrelevant outcome |
| Li L, 2022[81] | Irrelevant population |
| Lopez-Olivo MA, 2014 [82] | No mobile application |
| Luna IE, 2017 [83] | No mobile application |
| Lysack C, 2005 [84] | No mobile application |
| Makino K, 2021 [85] | Others, development of a wearable robot |
| Marsh J, 2014 [86] | Economic evaluation |
| Marsh JD, 2014 [87] | Others, virtual follow-up clinics |
| Mateo KF, 2020 [88] | Unsuitable time |
| McGinnis RS, 2016 [89] | Others, development of accelerometer system |
| Moffet H, 2011 [90] | Irrelevant outcome |
| Moore MR, 2202[91] | Full text/Abstract not available |
| Moore AJ, 2023[92] | Irrelevant outcome |
| Mouli VH, 2021 [93] | Irrelevant outcome |
| Naidu Helm A, 2023[94] | Full text/Abstract not available |
| Nelson M, 2021[95] | Irrelevant population |
| Nery ECHP, 2020 [96] | Irrelevant population |
| Nogueira JBS, 2018 [97] | Irrelevant outcome |
| Oatis C, 2020 [98] | No mobile application |
| Pastora-Bernal JM, 2017[99] | Review |
| Patterson JT, 2020 [100] | Irrelevant outcome |
| Penders A, 2018 [101] | Others, proof of concept for development of SEKO |
| Petersen W, 2021 [102] | Review |
| Pfeufer D, 2018 [103] | Review |
| Pietschmann J, 2019 [104] | No mobile application |
| Pila S, 2023[105] | Irrelevant outcome |
| Preston N, 2019 [106] | No mobile application |
| Preston NJ, 2023[107] | Others, Virtual follow-up clinic |
| Rantala A, 2020 [108] | Review |
| Rogante M, 2010 [109] | Review |
| Rognsvaag T, 2021[110] | Others, |
| Rognsvaag T, 2023[111] | Others, |
| Rosner BI, 2018[112] | Economic evaluation |
| Salehinia R, 2023[113] | Arabic Language |
| Seron P, 2021 [114] | Review |
| Shah RF, 2019 [115] | Irrelevant outcome |
| Shim GY, 2023[116] | Full text/Abstract not available |
| Shin G, 2019 [117] | Review |
| Shukla H, 2017 [118] | Review |
| Small SR, 2019 [119] | Review |
| Smith WA, 2015[120] | Full text/Abstract not available |
| Spangehl MJ, 2015 [121] | Others, commentary and perspective |
| Sprando D, 2017 [122] | Irrelevant population |
| Stuhlreyer J, 2022[123] | Irrelevant outcome |
| Terada H, 2012 [124] | Others, development of a wearable assist robot |
| Tousignant M, 2015 [125] | Economic evaluation |
| Turchetti G, 2016 [126] | Economic evaluation |
| Velayati F, 2020 [127] | Review |
| Veroff DR, 2013 [128] | Irrelevant outcome |
| Wang Q, 2021 [129] | Review |
| Wang X, 2018 [130] | Review |
| Wang X, 2019[131] | Review |
| Weinberg M, 2023[132] | Full text/Abstract not available |
| Windsor EN, 2021 [133] | Review |
| Wolfstadt JI, 2019 [134] | Perspective |
| Wong B, 2020 [135] | Review |
| Wylde V, 2022[136] | No mobile application |
| Yeowell G, 2020 [137] | Others, perspective on development of standard program |
| Yen C-H, 2017 [138] | Irrelevant population |
| Zachwieja E, 2020 [139] | Economic evaluation |
| Zhang J, 2019 [140] | Irrelevant outcome |
| Zheng H, 2013 [141] | No mobile application |
| Zhu Y, 2013 [142] | Others, development of an assistive device for patients for walking rehabilitation and for shortening the hospitalization period |

Table S4 Study characterstics of included studies (n=105)

| Author,Year, Country | Study design, Objective, population | Sample Size,  I*, C* | Technology (Acronym) | Purpose(s) | Period of intervention |
| --- | --- | --- | --- | --- | --- |
| Alexander JS, 2023, Crawford, 2021, USA[143, 144] | RCT, Effectiveness/efficacy, pre op | I: 256  C: 296 | Wearable sensor with mobile application (Mymobility) | Exercise, education, reminders | Post surgery to 6 weeks post op |
| An, 2021, S.Korea [145] | RCT, Effectiveness/Efficacy, pre-op patients | I: 40  C: 20 | Video call via smartphone or tablet | Physio session, communication, real time feedback of exercise | 4 weeks before TKA |
| Antunes, 2021, USA [146] | Longitudinal, Validation, post-op patients | I: 8 | Wearable motion sensors connected to mobile app | Remote monitoring of knee angle | Post-discharge |
| Argent, 2019, Ireland[147] | Mixed methods, Pilot testing, Post-op patients | I: 15 | Wearable motion sensors connected to mobile app | Self-monitoring, exercises, education remote monitoring of exercises, capturing PROMS | NR |
| Ayoade, 2011, UK [148] | ObservationalFeasibility, NR | NR | Wearable sensors (MARG) | Remote monitoring of ROM | NR |
| Backer, 2021  Germany [149] | RCT protocol  Effectiveness/Efficacy,post--op patients | I: 20  C: 15 | Sensor based device with mobile app | Visual feedback (Gamification) to aid quadricieps isokinetic exercises | Day 1 post-op to day 9 post-op |
| Bade 2020, 2023, USA [150] | Cross-sectional, Pilot testing, pre-op patients | I: 8  C:NA | Wearable sensor with mobile application (Loadsol) | Task training, education, progressive resisitive exercise, range of motion | Pre-op to 6 weeks |
| Bettger,2019, USA [151] | RCT, Effectiveness/Efficacy,post-op patients | I:143  C:144 | Sensor based device with computer application | Self-monitoring, information on exercises, tracking, remote monitoring of exercises, consultation | At hospital discharge to 3 months post-op |
| Bini, 2017, USA [152] | RCT, Effectiveness/Efficacy,post-op patients | I: 13  C:15 | Video-based asynchronous remote monitoring | Monitoring of patient progress, self-monitoring, video of exercises, decision support, communication. | Post-op 2 weeks onwards |
| Bitsaki, 2017, Germany [153] | Concept note of a clinical and cost effectiveness model | I: NA  C:NA | Mobile application, RESTful (Health API) | Measurement of symptoms, measurement of outcome such as WOMAC and QoL, education, monitoring of patient progress, decision making | Post-op to 12 months post-op |
| Blasco, 2022, 2023, Spain[154, 155] | RCT, Pilot/feasibility, post-op | I: 18  C: unclear | Web based (WeChat app) | Exercises, education, motivation, reminders, communication | One week after surgery to 12 weeks post op |
| Bolam, 2021, New Zealand [156] | Prospective cohort, Feasibility, Pre-op patients | I:14 | Wearable sensor with computer application | Remote monitoring of ROM | 0 to 12 weeks after surgery |
| Bonora, 2017, Europe [157] | Pre-post, Pilot testing, post-op patients | I: 13 | Wearable sensors with computer application | Biofeedback for ROM and gait | Day 4 post op to Day 9 post op |
| Calliess, 2014, Germany [158] | Pre-post, Validation/Reliability testing, pre-op patients | I: 6  C: NA | Wearable sensors with computer apllication | Remote monitoring of gait | 6 days pre-op to 12 months post op |
| Campbell, 2019, USA [159] | RCT, Effectiveness-Efficay, post-op patients | I: 76  C: 83 | Video and text message using bot, (StreaMD) | Motivation, recovery instructions, instructional therapy videos | Post-op day 1 to 6 weeks |
| Castle, 2018, Australia [160] | Cross-sectional, Reliability testing, post-op patients | I: 27 | Photo-based app for ROM measuring, DrG app | Remote measurment of knee movement | NA |
| Chapman, 2021, USA [161] | Pre-post, Feasibility study, Pre and Post op | I: 12 | Wearable sensor with computer application | Monitoring dynamic ROM at home | Post-op period not reported |
| Chiang 2017, Taiwan [162] | Longitudinal, Pilot tesitng , post-op | I: 18  C: NA | Wearable sensors with computer application | Remote monitoring for ROM | Post-op period not reported |
| Christensen,2022, USA[163] | Prospective cohort, pilot testing, post op | I: 3 | Mobile application with inbuilt sensors | Remote monitoring for gait | 2 weeks post op to 10 weeks post op |
| Chughtai, 2018, USA [164, 165] | Uncontrolled clinical trial, Feasibility, post-op patients | I:122 | Sensor-based device with computer application (VERA)-Virtual Exercise Rehabilitation Assistant | Self-monitoring, tele-consulation, remote monitoring of exercise, real time feedback to patients, measurement of outcomes such as pain and functions. | Post-op to unclear |
| Chughtai, 2019, US [166] | Comparative cohort study, Effectiveness/Efficacy, pre-op patients | I: 114  C:362 | Telerehabilitation program, PreHab (PeerWell) | Education, exercise, mindfullness and nutrition,medical risk reduction and pain management | Pre-op until surgery |
| Colomina, 2021, Spain [167] | NRCT, Effectiveness/efficacy; post-op patients | I:29  C:30 | Mobile health intergreted care model, (SACM-mhealth-IC) | Self-management, feedback, education, and communication | Post-op period not reported |
| Correia, 2018, 2019, Portugal [168, 169] | Non randomised controlled trial, Pilot and feasibility, post-op patients | I: 30  C:29 | Waerable sensor with mobile application and web portal | Real time biofeedback, remote monitoring progress of physio sessions. | Day of recruitment to post-op week 8 |
| De Berardinis, 2022, Italy[170] | Restrospective cohort, Pilot testing, post-op | I: 18  C: 81 | Wearable sensor with mobile application (KARI) | Video consultation, physio session, remote monitoring | 8-10 days post op |
| De Vroey, 2018, Belgium [171] | Cross-sectional, Reliability, post-op patients | I: 16 | Wearable sensors with computer application | Remote monitoring of gait | Post-op to 12 months |
| Doiron, 2016, 2020, Canada [172, 173] | RCT, Pilot and feasibility, pre-op patients | I:23  C:11 | Videoconfenrencing app | Communication, exercises and decision support | Pre-op to post-op 12 weeks |
| Duong, 2022, 2023, Wang X, 2021 Australia [174-176] | RCT, Effectiveness/efficacy, post-op patients | I: 102  C:102 | Wearable sensors woth mobile app, (Physi App, Fitbit with Fitbit app, iMessage app, Goniometer pro) | Exercises, reminders, communication, goal setting, self-monitoring progress,measurement of step count, sleep, and activity, remote monitoring of ROM | Post-op to 6 months post-op |
| Eichler, 2017, 2019, Europe [177, 178] | RCT, Effectivenss/efficacy, post-op patients | I: 48  C: 39 | Sensor based device with computer application (MyRehab) | Audio-video consultation, remote monitoring of exercises and ROM, self-monitoring, appointment, exercises | After 3 weeks of inpatient rehab - 3 months post-op |
| Farr- Wharton, 2017, Hussain 2017[179, 180] | RCT, pilot testing, pre op patients | Unclear | Wearable sensor with mobile app | Physio sessions, psychoeducation sessions, self monitoring, goal setting, rewards, motivation, remote monitoring of patient progress, activity level tracking | 4 week pre op-16 weeks post op |
| Ficklscherer, 2014, Germany[181] | RCT, pilot testing, pre op patients | I: 15  C: 11 | Sensor based device with computer application | Physio session | First day post op-until discharge |
| Fisher, 2019, LeBrun,  2022, USA [182, 183] | Retrospective, Effectiveness/Efficacy, post-op patients | I: 82  C: 244 | Tele health video visit, (MyChart) | Review of home exercise program, assessment of current pain level and functional mobility, remote monitoring of gait pattern and motion | 24 hours of discharge to 3 to 4 weeks post-op |
| Fung 2012, Canada [184] | Preliminary RCT, Pilot testing, post-op patients | I: 27,  C: 23 | Sesor based device with computer application (Wii Fit) | Physio-sessions using gamification | Post surgery unitl discharge from physiotherapy |
| Gianola 2020, Italy [185] | Phase 3-RCT, Effectivenss/Efficacy, post-op patients | I:44  C:41 | VR based rehabilitation | Functional exercises | Post-op to NR |
| Gohir 2021, UK [186] | RCT,Effectiveness/efficacy, pre-op patients | I: 67  C: 67 | Mobile application (ibeat-OA) | Communication, education materials, reminders, exercises, motivation, goal setting, appointment notification | Upto 6 weeks post surgery |
| Gray, 2022, England[187] | Retrospective cohort, Effectiveness/efficacy | I: 287  C: 873 | Mobile application (Digital Joint School using GoWell health program) | Education resources, remote monitoring progress, two way communication, track patient engagement and monitor patient use | Pre surgery listing to 6 months post op |
| Gunduz, 2021, Turkey[188] | NRCT, Effectiveness/efficacy, pre-op | I: 40  C: 40 | Web-based | Pain management | One day before surgery to zeroth day post op |
| Hasse 2004, Austria [189] | RCT, Effectiveness/Efficacy , post-op patients | I: 79  C:72 | Wearable sensors with computer application | Physio sessions, feedback, consultation, and remote monitoring of exercise | 23-42 days post-op to 6 months |
| Hadamus 2020, 2022, Poland [190] | Non randomised controlled trial, Effectiveness/efficacy, post-op patients | I: 38  C: 21 | Sensor based device with computer application (VBC project) | Remote monitoring of gait, rehabilitation exercises, feedback using gamification | Post-op within 7-One month |
| Hardwick-Morris, 2022, Australia[191] | Cohort, Effectiveness/efficacy, pre and post op | I: 124  C: 62 | Mobile application | Individualised and progressive exercise regimen via digital platform | Pre surgery to 6 weeks after discharge |
| Harmelink 2017, Netherlands [192] | RCT, Protocol, post-op patients | I: 0  C:0 | Wearable sensors with mobile application | Motivation, awareness, reminders, self management, exercises, remote feedback and activity level | 2 weeks post-op onwards. |
| Hong, 2022, USA[193] | Longitudinal, Pilot testing, pre- & post-op | I: 22  C: 31 | Mobile application (Digital musculoskeletal surgical care program app) | Video consultation, exercises, education materials, health coaching | 8 weeks pre-op to 12 weeks post op |
| Huang P 2017, China [194] | Non randomised controlled trial Effectiveness/Efficacy, post-op patients | I: 75  C:75 | Mobile application | Education, Physiotherapy sessions and followup, | First-day post-op to 3 months post-op |
| Huang Y.P 2020, Taiwan [195] | Uncontrolled,  Validation/Reliability, Post-op patients | I: 8  C: 16 | Wearable sensor with mobile app | Remote monitoring of ROM | NR |
| Hung 2018, Taiwan[196] | Proof of concept Development, NR, NR | NR | Wearable sensors with computer application | Remote monitoring of rehabilitationby physiotherapist | NR |
| Janhunen, 2023, Finland[197] | RCT, Effectiveness/efficacy, post op | I: 21  C: 25 | Sensor based device with mobile application | Gamification(exergaming) | Post surgery day 3 to 4 months |
| Jansson 2020, Finland [198] | RCT Protocol, pre- & post-op patients | I: 0  C:0 | Mobile app, DPJ (Digital Patient Journey) | Education, remiders, notifications, communication, goal setting, self-monitoring | During pre-op clinic visits |
| Jenny 2013, 2016, France [199, 200] | Pre-post, Validation, post-op patients | I:10 | Mobile app without sensors | Remote monitoring of knee flexion angle | Post -op period |
| Juhl 2016, Denmark [201] | RCT, Effectiveness/Efficacy, post-op patients | I:70  C:71 | Wearable sensors with mobile application (ICURA) | Exercises, remote monitoring of exercises, communication | Post-op for 6 weeks |
| Klement 2019, USA [202] | Retrospective cohort, Effectiveness/Efficacy, post-TKA patients | I: 296  C: NA | Web-based platform (SDPT) | Exercise-sessions | Post-discharge onwards |
| Kline 2019, USA [203] | RCT Protocol, pre-op patients | I: 0  C: 0 | Wearable sensor with mobile app | Measurement of outcome such as physical activity, daily step counts, life space assessment, physical function, QoL, self monitoring, feedback, goals, communication and motivation | Pre-op to 6 months post-op |
| Knapp 2021, USA [204] | Longitudinal, Pilot testing/feasibility, pre-op patients | I: 112 | Mobile application | Education material | Pre-op 6 weeks- post-op 12 weeks |
| Kontadakis 2020, Greece[205] | Longitudinal, Pilot testing/feasibility, post-op patients | I: 10 | Wearable sensors with mobile application | Remote monitoring of ROM, Real time feedback, improve patient engagement during physiotherapy | Within 12-14 days post op onwards |
| Kramer 2003, Canada[206] | RCT, Effectiveness/efficacy, pre op patients | I: 80  C: 80 | Telephone calls | Exercise, advice on wound care, scar treatment and pain control | Week 2 post op-week 12 post op |
| Krebs 2006, USA [207] | Cross-sectional Pilot testing/feasibility, post-op patients | I:10  C:10 | Wearable sensor with computer application (IDEEATM, MiniSun) and electrogoniometers (elgons) | Mesurement of knee flexion | NA |
| Kuether 2019, USA [208] | Retrospective cohort, Pilot testing, pre-op patients | I: 40  C:614 | Sensor device with computer application, VERA (Virtual Exercise Rehabilitation Assistant) | Exercises, tracking, feedback, communication | At the time of surgery to 3 months post-op |
| Lam 2016, Canada [209] | Cross-sectional, Pilot testing/feasibility, post-op patients | I: 26 | Sensor based device with computer application | Remote monitoring of exercises, patient movement, asess recovery progress, appointment scheduling | NA |
| Lebleu, 2023, Belgium[210] | Cohort, Pilot testing/feasibility, Post | I: 127 | Wearable sensor with mobile application | Education, exercise, communication, remote monitoring progress, counseling, decision support | Day of surgery to 6 months post op |
| Levinger 2015, Australia [211] | Case study, Feasibility, post-op patient | I: 1 | Sensor based device with computer application | Remote monitoring of gait, real time biofeedback, communication | Post-op 4-6 weeks to post-op 6 weeks |
| Li, 2023, China[212] | NRCT, Effectiveness/efficacy, post op | I: 50  C: 50 | Web-based | Education, self-management, remote monitoring of progress, peer support | Pre surgery |
| Liptak 2019, Australia [213] | Single blinded RCT, Protocol, pre-op patients | I:0  C:0 | Sensor based device with mobile application (MAXM skate app) | Exercises, self monitoring, remote monitoring of ROM | Pre-op onwards |
| Losina 2013, Australia [214] | RCT, Protocol, pre-op patients | I: 0  C:0 | Telephone calls (AVIKA) | Motivation/Goal setting | Post-op 1 months to post 22 weeks |
| Lou, 2022, China[215] | Cross-sectional, Validation/reliability, post op | I: 10  C: 10 | Wearable sensor with computer application | ROM | Post op |
| Lu 2021, China [216] | RCT,Effectiveness/Efficacy, post-op patients | I: 39  C: 39 | Micro-video app, (wechat) | Communication, physio sessions, goals, education, patient monitoring, peer and health care support education | Post-op day 1 onwards |
| Mark 2021, Europe[217] | RCT, Protocol , post-op patients | I:0  C:0 | Mobile app (ICURA) | Measurement of quantity and quality of exercises, rempote monitoring of patient exercise adherance and progress and physio sessions | Post-op 6 weeks to NR |
| Marques 2020, Germany [218] | Cross-sectional, Validation,  healthy volunteer | I:3  C:NA | Wearable sensors with mobile app  (BPM pathway-BIOPAC Goniometer) | Remote monitoring of ROM, exercises, self monitoring, real time biofeedback and communication | NA |
| Mcdonall, 2016, 2019, 2022, Australia [219-221] | Cluster crossover trial, Effectiveness/efficacy , post-op patients | I: 104  C: 137 | Nurse-led multimedia intervention | Education, daily goal setting | Post-op day 1 to 2 weeks post op |
| Mehta 2017, 2020,2021, USA [222-224] | RCT, Effectiveness/Efficacy, Post op | I: 147  C: 153 | Wearable sensors and mobile application | Physical activity monitor, bidirectional textmessaging to monitor progress, pain gamification, tracking, goal, communication, motivation, self-monitoring, feedback, remote monitoring of progress | Following discharge |
| Milliren, 2022, USA[225] | Quasi experimental, Effectiveness/efficacy | I: 694  C; 613 | Web based (Ubicare Smart X) | Messaging | Presurgery |
| Msayib 2017, UK[226] | Development, post-op patients | Not mentioned | Wearable sensors | Remote monitoring of knee flexion, feedback, communication | NA |
| Na, 2021, Texas[227] | Cross-sectional, Validation/reliability, pre op patients | I: 26  C: 13 | Wearable sensors | Measuring gait instability | NA |
| Negus 2014, Australia [228] | RCT, Protocol, post-op patients | I: 0  C:0 | Sensor based device with computer application (Wii-Fit) | Exercises, real time feedback, self-monitoring | Post-op 6 week to not mentioned |
| Neuvo, 2021, 2023, Spain[229, 230] | RCT, Effectiveness/efficacy, post-op | I: 23  C: 22 | Wearable sensors with mobile application (ReHub) | Remote rehabilitation training, and monitoring adherence and progress | Post surgery day 2- 4 weeks |
| Onyeukwu 2020, USA [231] | RCT, Pilot testing, post-op patients | I: 19  C:19 | Wearable sensor with mobile app (InterACTION) | Self-monitoring, exercises, remote monitoring of exercises and ROM, real time feedback, communication and measurement of adherance and clinical data | Post-op first visit onwards |
| Osterloh, 2023, Germany[232] | RCT, Pilot testing/feasibility, post-op | I: 7  C:4 | Web based.video (YOLii) | Remote physio session | Fourth week post op to 6 months |
| Park 2017, S. Korea [233] | RCT, Effectiveness/Efficacy , post-op patients | I:21  C:19 | Telephonic counselling and messaging | Counseling sessions, | 1 week post-op to 11 weeks |
| Park, 2023, South Korea[234] | RCT, Effectiveness/efficacy, post op | I: 29  C: 29 | Web based | Education, rehabilitation exercise, aroma therapy and phone counselling | Day of discharge to 5 weeks after discharge |
| Pellagrini 2021, USA [235] | RCT, Protocol, post-op patients | I: 0  C:0 | Mobile application | Appointments, information about pre-, post op, linkage with rehab facilities & with pts, education, Goal, reminder, notification | 4weeks post-op to NR |
| Pereira, 2017, Switzerland [236] | Cross-sectional, Reliability testing, Post op and healthy volunteers | I: 60 | Mobile application with inbuilt sensor | Remote monitoring of ROM | NA |
| Pfeufer,2020, Germany[237] | Longituidinal cohort, Validation study of post knee replacement patients | I:29 | Sensor based device with computer application | Measurement of gait parameters | NA |
| Piqueras 2013, Spain [238] | RCT,Effectiveness/Efficacy, post-op patients | I:70  C:70 | Wearable sensors with computer application (Interactive virtual rehabilitation) | Remote monitoring of patient movements and exercises, physio sessions, recording for later evaluation, quantification of exercise sets, number of repitions, & knee-flexion angles | Week 1- week 3 post op to NR |
| Pournajaf, 2017, 2022, Italy [239] | RCT, Effectiveness/efficacy, Post op patients | I: 31  C: 31 | Sensor based device with computer application | Balance training | 10 days post op-3 weeks |
| Pronk 2021, Netherlands[240] | RCT,Effectiveness/Efficacy, pre-op patients | I: 38  C:33 | Mobile app, PainCoach group | Education on pain medication use, physiotherapy exercises, measurement of pain level | Within 14 days post-op to 1month post-op |
| Ramkumar 2019, USA [241] | Prospective cohort, Pilot testing, pre-op patients | I: 25 | Wearable sensor with mobile application | Exercise, reminders, measurement of preoperative mobility (daily steps) and PROMs opioid consumption, exercise compliance, remote monitoring of ROM and daily compliance check | Pre-op day of recruitment to 3 months post-op, |
| Russel,2003, 2011, Australia [242, 243] | Prospective RCT, Effectiveness/Efficacy, post-op patients | I: 31  C:34 | Computer-based telerehabilitation | Consultation, education, physio session, remote monitoring of physical performance | One week of discharge- 6 weeks of post-op |
| Scheper 2019, Netherlands[244] | Prospective cohort, Pilot testing, post-op patients | I: 69 | Mobile app (Woundcare) | Self monitoring of pain, trigeer to contact physician, reminders | Immediate post-op period |
| Smulders 2021, Netherlands [245] | Cross-sectional, Validation/Reliability, pre-op patients | I: 25  C:27 | Wearable sensors with computer application | Measurement of gait | NA |
| Stauber 2021, Austria [246] | RCT, Protocol, pre- & post-op patients | I: 0  C:0 | Mobile app, RECOVER-E | Self management, Education material, Reminder, Communication, Self monitoring, Motivation | 4 to 6 weeks pre-op to 3 months post-op |
| Straat, 2023, Netherlands[247] | RCT Protocol, pre-op patients | I: 0  C:0 | Wearable sensors with mobile application, ikHerstel app | Education, self-management, remote monitoring of progress, self monitoring, goal setting, active referral | 2-4 weeks pre-op |
| Strahl, 2022, Germany[248] | RCT Protocol, pre-op patients | I: 0  C:0 | Mobile app, ALLEY app | Education material, self-monitoring, remote monitoring | During surgery to patient can use after trial |
| Su 2016, Taiwan[249] | NRCT,Effectiveness/Efficacy, post-op patients | I: 18  C: 16 | Sensor based device with computer application | Remote monitoring of motion, rehabilitation exercises, feedback using gamification | 2 days after surgery onwards |
| Summers, 2023, USA[250] | Cohort, Effectiveness/efficacy, pre op | I: 135  C: 135 | Sensor based device with computer application | Electro-mechanical therpay device for remote rehabiliation and monitoring | Post surgery to 3-6 weeks |
| Szöts 2016, Denmark [251] | RCT,Effectiveness/Efficay, post-op patients | I: 59  C: 58 | Telephone follow-ups | Communication and education | 4^th^ day post-op to 14^th^ day |
| Timmers 2019, Netherlands [252] | RCT, Effectiveness/Efficacy, post-op patients | I: 114  C: 99 | Mobile application (Patient Journey App) | Education and reminders | 1^st^ day post-op to 28^th^ days |
| Torpil, 2022, Turkey[253] | RCT, Effectiveness/Efficacy, post-op patients | I: 21  C: 21 | Web based | Home modification & transfer training with client centered intervention which is goal setting, therapy plan, and patient feedback | Post op to 12 days post op |
| Tousignant, 2010, 2011, 2015, Moffet, 2011, 2015, Cabana, 2010, Canada [254-259] | RCT, Effectiveness/Efficacy, post-op patients | I: 24  C: 24 | Remote technology (Video conferencing) | Physio sessions and consultations | Day of discharge to 2 months post-op |
| Tripuraneni 2021, USA[260] | RCT, Effectiveness/Efficacy, post-op patients | I: 153  C: 184 | Wearable sensors with mobile app | Self monitoring, physio sessions, reminders, measurement of heart rate, heart rate variability, steps, flights of stairs climbed, and stand hours | 2 weeks pre-op to 6 weeks post-op |
| van Dijk-Huisman 2021, Netherlands[261] | NRCT, Pilot study, quasi-experimental design, post-op | I: 33  C: 64 | Wearable sensors with mobile application (Hospital Fit app) | Remote monitoring of patient activity, exercise videos and self-management | Post-op to NR |
| Visperas, 2021, USA [262] | RCT, Pilot testing, pre-op | I: 200  C: 200 | Web based, (JointCOACH web based IPSP platform) | Consultation, education, exercises | Day when surgery is scheduled-post-op 90 days |
| Wang Q, 2-22, 2023, China[263, 264] | RCT, Effectiveness/efficacy, post-op | I:20  C: 16 | Mobile application (We Chat app) | Education, Video exercise guide, telphone contact and motivation and councelling | Post op to 6 weeks after discharge |
| Yang, 2023, China[265] | RCT Protocol, pre-op | I: 0  C: 0 | Wearable sensors with mobile application | Rehabilitation exercise, remote monitoring progress, self monitoring | Post surgery-24 weeks |
| Youn, 2018, USA[266] | Cross-sectional Validation/Reliability, post-op patients | I: 18  C: NA | Wearable sensors with computer application | Measurement of gait | NA |
| Zhang Haohua 2020, Asia [267] | Prospective cohort, Validity/reliability testing , post-op patients | I: 10  C:NA | Remote automatic tech, Wearable sensors | Remote monitoring of knee function | Immediately post-op |
| Zhang Xianzuo 2021, Asia [268] | NRCT, Effectiveness, post-op patients | I: 2292  C: 1236 | Social media app, WeChat | Education, Communication | During hospital stay post-op |
| Zhao 2021, USA[269] | NA, Development, NR | NA | Wearable sensors with computer application( Microsoft Kinect sensor) | Monitoring of exercise using sensors | NA |
| Zheng Qianpeng 2017, USA [270] | NA,Development, post-op patients | NA | Sensor-based device with mobile application | Remote monitoring of limb weight load and gait | Post-op |

**I: Intervention *C: Control *NR: Not reported * NA: Not applicable*

Table S5 List of studies with exercise as a rehabilitation intervention component (type of exercises delivered via technology)

| **Author, year** | **Mode of delivery** | **Group of exercises** | | | | | |
| --- | --- | --- | --- | --- | --- | --- | --- |
|  |  | **Range of motion exercises** | **Muscle strengthening exercises** | **Balance exercises** | **Exercises to improve gait** | **Functional exercises** | **Others (warm-up, cool down, endurance)** |
| Alexander, 2023[143] | App based exercise | NA | NA | NA | NA | NA | NA |
| An, 2021[145] | Two-way video call | Supine leg slides (supine and standing), ankle pumps, self-passive knee extension, quadriceps, and hamstrings stretching, trunk rotation, knee ROM exercises | SLR, bridges, knee press, quadriceps strength, abductor strength | Tandem gait, balance training | Gait training with walker | Mini squats, walking up and down stairs | Endurance exercises |
| Argent, 2019[147] | Exercise guidance via tablet | NA | NA | NA | NA | NA | NA |
| Backer, 2021[149] | Gamification | ^*^NA | Knee press, SLR | NA | NA | NA | NA |
| Bettger, 2019[151] | Virtual PT program | NA | NA | NA | NA | NA | NA |
| Bini, 2017[152] | Instructional exercise videos | NA | NA | NA | NA | NA | NA |
| Campbell, 2019[159] | Short instructional videos | NA | NA | NA | NA | NA | NA |
| Corriea, 2019[168] | Video demonstration | NA | NA | NA | NA | NA | NA |
| Chughtai, 2018[164] | virtual exercise rehabilitation assistant | NA | NA | NA | NA | NA | NA |
| Chughtai, 2019[166] | App (mode not clear) | NA | NA | NA | NA | NA | NA |
| De Berardinis, 2022[170] | Not clear | NA | NA | NA | NA | NA | NA |
| Doiron, 2019[172] | Two-way video call | Knee flexion and extension, ankle dorsiflexion | Clamshell exercises, hip abduction in standing and side lying, hip flexion in supine and standing, resisted hip flexion, SLR in supine, bridging with both legs, unilateral bridge in supine position, resisted hip extension in standing, adductor isometrics in sitting, hip adduction in side lying, sitting knee extension with or without weights on ankle, standing knee flexion resisted sitting knee flexion | Tandem walking, balancing with feet together, unipodal | NA | NA | Cycling, walking |
| Duong, 2023[175] | Health fitness app (mode not clear) | NA | NA | NA | NA | NA | NA |
| Eichler, 2019[177] | Exercise demonstration by avatar | NA | Strengtheing exercises | Exercises to improve postural control | NA | NA | NA |
| Farr- Wharton 2017[180] | Exercises videos in mobile app | NA | NA | NA | NA | NA | NA |
| Fisher, 2022[182] | Virtual platform | Ankle pumps, seated active knee flexion and extension, passive knee extension stair stretching | Quadriceps sets, gluteal sets, | NA | NA | NA | NA |
| Fung, 2012 [184] | Wii fit gaming activity | NA | NA | Multi directional weight shift | NA | NA | NA |
| Gianola, 2021[185] | Virtual reality | Heel slides | Short-arc quadriceps, hip adductor strengthening, SLR in supine and side-lying, bridging, long arc quadriceps, standing leg curls, standing hip extension, hip abduction in standing | Supine and standing target proprioception, balancing on balance board | NA | Active bilateral squat | NA |
| Gohir, 2021[186] | Exercise program via app (iOS or android) | NA | Leg strengthening (sit-to-stand) | NA | NA | Stair climbing | Core stability |
| Gray, 2022[187] | Videos | NA | NA | NA | NA | NA | NA |
| Gunduz, 2021[188] | Exercise videos | NA | NA | NA | NA | NA | Breathing exercises |
| Hadamus,  2022[190] | Virtual reality | NA | NA | Balance exercises | Gait training exercises | NA | NA |
| Hardwick, 2022[191] | App (iPad), mode not clear | NA | NA | NA | NA | NA | NA |
| Hasse, 2004[189] | Computer aided, multimedia, real-time training exercises | NA | NA | NA | NA | NA | NA |
| Huang, 2017[194] | Mobile app  (mode not clear) | NA | NA | NA | NA | Functional exercises | NA |
| Janhunen,2023[197] | Exergaming | Knee flexion extension, hams stretching | Squats | Balance and coordination | NA | Functional exercises | NA |
| Juhl, 2016 [201] | Predefined exercise program in app | NA | NA | NA | NA | NA | NA |
| Klement,  2019[202] | Exercises via email in form of videos, written instructions, and pictures | NA | NA | NA | NA | NA | NA |
| Knapp, 2021[204] | Modules in mobile app | NA | NA | NA | NA | NA | Chair exercises, standing exercises, bed exercises, floor exercises |
| Kuether, 2019[208] | Virtual exercise rehabilitation by animated avatar | NA | NA | NA | NA | NA | NA |
| Lam, 2016 [209] | Exercise demonstration via automated rehabilitation system | NA | NA | NA | NA | NA | NA |
| Lebleu, 2023[210] | App based exercises | NA | NA | NA | NA | NA | NA |
| Li, 2023[212] | Not clear | NA | NA | NA | NA | Functional exercises | NA |
| Lu, 2021[216] | Micro videos via WeChat | NA | NA | NA | NA | NA | NA |
| McDonall, 2022[221] | Animated exercises | NA | NA | NA | NA | NA | NA |
| Nuevo, 2023[230] | Not clear | NA | Strengthening of quadriceps, hamstrings, hip flexors, extensors, and abductors in standing | NA | NA | NA | NA |
| Onyeukwu, 2020[231] | interACTION platform (mobile app and wearable sensors) | NA | NA | NA | NA | NA | NA |
| Osterloh, 2023[232] | Video-based tool- YOLii | Hip abduction- adduction and flexion-extension in standing, knee flexion extension in standing, cycling in supine | Crunches, side crunches, bridging, modified bridging with one leg stretched, | Repetitive one-legged stand, stand on tiptoes, tap on stepper, weight shift on balance pad, steps on balance pad, maintain one-legged stand, squat on balance board |  | modified dead bug-legs 90°flexed in air, modified dead bug arms isolated with heels on the floor jump side to side, step side to side, step before and behind, step ups, | Warm-up and cool-down exercises |
| Park, 2023[234] | Not clear | NA | NA | NA | NA | NA | NA |
| Pournajaf,2022[239] | Virtual reality-based games | NA | NA | Balance training | NA | NA | NA |
| Piqueras, 2021[238] | Interactive virtual system | NA | NA | NA | NA | NA | NA |
| Pronk, 2020[240] | Exercise videos | NA | NA | NA | NA | NA | NA |
| Ramkumar, 2019[241] | Avatar depicting exercises via mobile app | Heel slides | SLR, standing leg curls, long arc quadriceps | NA | NA | NA | NA |
| Russell, 2011[242] | Video conference | NA | NA | NA | NA | NA | NA |
| Su, 2015[249] | Gamification | NA | NA | NA | NA | NA | NA |
| Timmers, 2019[252] | Exercise videos | NA | NA | NA | NA | NA | NA |
| Tousignant, 2011[254] | Video conference | NA | NA | NA | NA | Functional exercises | NA |
| Tripuraneni, 2021[260] | Exercise program via mobile app | NA | NA | NA | NA | NA | NA |
| Van Dijk-Huisman, 2020[261] | Exercise videos via mobile app | NA | Muscle strength | NA | NA | Functional exercises | Exercises to improve physical fitness |
| Visperas, 2021[262] | interactive patient-provider software platform (IPSP), mode not clear | NA | NA | NA | NA | NA | NA |
| Wang, 2023[263] | WeChat app | Sitting knee flexion and extension, hip abduction and adduction, heel slides | SLR, bridging, knee flexion an extension under resistance, calf raises, | Balance and proprioceptive training | Walking using a walker, stick or cane, walking without aids, | Standing march, stepping and curbs, mini squats, walking with longer steps (strides) | NA |
| Zhang, 2021[268] | Not clear | NA | NA | NA | NA | NA | NA |

NA- Not Available, the information about the exercises were not available in these studies

n= 52 studies have given exercises via technology

n= 11 were for telemonitoring and no exercises has been given

n=32 have not described the type of exercises in above table

Table S6 List of studies that have education as a component of rehabilitation provided via any mode

| **Author, year** | **Mode of delivery** | **Education content** |
| --- | --- | --- |
| An, 2021[145] | Not clear | Home exercises safety |
| Chughtai, 2019[166] | E-instructions | Exercise instructions, nutritional advice, mindfulness programs to reduce anxiety, education regarding home safety and medical risk reduction and pain management skills |
| Dorion, 2019[172] | Not clear | Medication usage and ice application |
| Gohir, 2021[186] | Not clear | Basics of osteoarthritis and its treatment, self-managing symptoms, the benefits of behavioral change and maintaining a healthy lifestyle |
| Gray, 2022[187] | Digital formats (PDF docs, videos, interactive forms, email etc.) | Information about  surgery, lifestyle, and wellbeing support,  pre-habilitation advice and support, and care after surgery |
| Hardwick, 2022[191] | Sent to iPad (form NA) | Hospital stays, postoperative pain and swelling management, use of walking aids, and how to negotiate an array of difficult daily activities postoperatively |
| Huang, 2017[194] | Mobile app | NA |
| Hussain, 2017[179] | Texts and voice recordings | NA |
| Knapp, 2021[204] | Modules in mobile app | Safe sex after surgery, how to get in and out of the car, taking a shower, how to do everyday movements, get your recovery essentials ready, mindful breathing, dealing with nausea, mind and body awareness, dental work timing, prepare your home, travelling with new joint, making sure your caregiver is prepared, medication inventory, know the red flags, find hazards at home,  nutrition planning and education, anxiety stress and pain management, social and environment preparation and health literacy |
| Lebleu, 2023[210] | Articles via app | NA |
| Lu, 2021[216] | Micro videos via WeChat | Cognitive belief education and exercise behaviour |
| Li, 2023[212] | Face-to-face | Correct use of medications, nutritional advice |
| Milliren, 2022[271] | Multimedia messages in form of texts, videos, slideshows, images, and interactive tools | Patient expectations about procedure, post-operative recovery, and the return to routine activities |
| Park, 2023[234] | Face-to-face and booklet | General understanding of knee OA and TKA, precautions for daily life after discharge, dietary therapy, prevention of blood clotting, prevention of infection, and activities to avoid |
| Visperas, 2021[262] | Not clear | information, postoperative housing preparation, instructions  about how to prepare for surgery, information about the surgery, information about medications and pain control, and  information about postoperative recovery and rehabilitation. |
| Wang, 2023[263] | Short text messages and pictures | Precautions after TKA, how to conduct muscle relaxation, lived experiences, what is depression and why it may occur after surgery, and what supports patients can seek |
| Zhang, 2021[268] | WeChat app | NA |

NA= studies that provided education content as a rehabilitation component but not given

n= 17 studies have delivered education

Table S7 Healthcare providers perspective on use of technology/telerehabilitation/mobile application for rehabilitation monitoring (N=11)

| **Author, Year, Region, Study design, Sample size** | **End users and technology used** | **Key themes on healthcare professional perception/experiences and satisfaction** |
| --- | --- | --- |
| Argent, 2018, Europe[147]  Qualitative study using semi-structured interviews,  N=10 | 4 physiotherapists, 2 clinical nurse specialists, 2 orthopaedic assistants, 1 occupational therapist and 1 staff nurse  Single inertial measurement units (IMUs) (Shimmer, Dublin, Ireland) prototype and an Android application developed for a tablet, used by patient at home. | To gain an understanding of:   1. **Current methods of monitoring and feedback:** objective markers such as range of motion, swelling, pain and functional scores used to monitor changes in the patient’s condition. Lack of objectivity while assessing muscle strength, gait, and exercise technique and patient self-report formed basis for ongoing monitoring. 2. **Perceptions and knowledge of wearables:** participants were aware of wearable technology, felt that it motivates the user to meet their goals, gives opportunity to track their own data and analyse their results. One participant felt that wearables are not beneficial, and others reported number of choices are overwhelming, and questioned the validity. 3. **Opportunities and challenges for connected health in orthopaedic rehabilitation:** participants agreed that there is wide scope for connected health interventions to play a role in orthopaedic rehabilitation. Features such as progress tracking, feedback, remote monitoring, regular range of motion measurement and counting repetitions useful.   **Challenges:** compliance, reliability and validity of technology, internet capability, bugs, glitches, imbalance in patient-clinician relationship, one’s ability and confidence with technology.  **Perceived impact of exemplar system:** perceived as having a positive impact in orthopaedic rehabilitation. Major benefit: ability to capture and track data in a manner that has not previously been possible. System can improve patient adherence and contribute to a more efficient healthcare system. |
| Boissy T., 2011,  North America[272]  Descriptive study embedded in Randomized Controlled Trial  N=48 | Physiotherapists  Telerehabilitation platform of two H264 videoconferencing CODECs (Tandberg 550 MXP) with integrated wide-angle view cameras, remote-controlled pan, tilt zoom (PTZ), 20-inch liquid crystal display (LCD) screens and a dedicated modular software interface for user-friendly control of videoconferencing connections, PTZ camera functions, and external devices | **Physiotherapist satisfaction:**   - *Reliability of the technological environment:* the physiotherapists found it satisfactory (45.5%) and good for (54.5%). - *Voice/image synchronization:* satisfactory (13.6%) and good (86.4%) of the time. - *Refresh rate of the images:* satisfactory (22.7%) and good (77.3%) of the time. - *Sound quality:* satisfactory in (13.6%) and good (86.4%). - *Operability of the peripherals:* insufficient (5%) of the time, satisfactory (13%), and good (82%). - *Quality of the overall technical environment* was good (80%) and insufficient (3%) - *Quality of the image/voice synchronization* was good (84%) and insufficient (2%) - Q*uality of the image frame refresh rate* was good (84%) and insufficient (3%) - S*ound quality* was good (85%) and insufficient (2%) - *Quality of the operability of peripherals* during the session was good (89%) and insufficient (1%) |
| Haase, 2004, Europe[189]  Randomized controlled trial, mixed methods,  N=68 | Physicians and therapists  Computer aided multimedia training for remote physiotherapy and communication | **Acceptability**   - Course and arrangement of training was perceived positively by the physicians & therapists - They felt arrangement are attractive and movement descriptions are vivid - Emphasised on video animations and audio sequences   Majority felt computer training is as effective as conventional training. Three physicians even felt computer training is more effective because patients were more diligent and careful in carrying out multimedia led exercises |
| Jansson, 2019, Europe[273]  Qualitative study using semi structured interviews,  N=20 | 4 surgeons, 2 anaesthesiologists, 10 nurses, and 4 physiotherapists  Information gathering on proposed eHealth needs. | **Nine main categories for the proposed eHealth needs:**   - Needs related to eligibility criteria - Needs related to referrals - Needs to meet Health Care - Needs in patient flow - Needs in post discharge care - Needs in patient counselling - Needs in communication - Needs in transparency   Needs in receiving feedback |
| Massip, 2020, Europe[274]  Descriptive study embedded in Randomized Controlled Trial,  N=21 | Healthcare professionals  mHealth-enabled Integrated Care (IC) model, including a self-management app for patients, a set of integrated sensors, and a web-based platform connecting professionals from different settings or usual care | **Satisfaction with the IC platform in IC arm staff:**  Overall Net Promoter Score (NPS)= 35%.  The mean System Usability Scale (SUS) score for staff was 62 (SD 19). |
| McDonall, 2022, Australia[221]  Hybrid Type II Implementation study, n= 4 | Clinical nurse leaders  Multimedia intervention via iPad in the context of acute postoperative care | **Concerns**   - **Potential burden of increase in workload to nursing staff**- concern in using the iPad and assisting patients to navigate the program first time may be time consuming and increase workload. - **Limitation in use of technology by aged patients**- 50% of the nurses indicated that older patients may be unfamiliar with portable devices or unable to use them.   **Safety and storage of equipment** **in patients’ room** - nurses worried about physical location of the iPad in patients’ rooms, additional clutter, risk of being stolen or broken, and risk of infection due to cross-contamination. |
| Onyeukwu, 2020, North America[231]  Randomized controlled trial, mixed-method,  n=19 | Physical therapists  Remote (wearable) rehabilitation monitoring platform (interACTION) for home exercise program | **Satisfaction**   - Six physical therapists would consider using interACTION to manage rehabilitation again in the future   **Technology**  *Affirmations*   - Provided motivation/accountability - Reports and tracking useful   *Criticism*   - Calibration or counting errors led to inconsistent measurements of range of motion   *Suggestions*   - Simplify the setup for patients - Provide audio/voice feedback to the patient   Increase accuracy |
| Parkes, 2019, UK[275]  Mixed methods study, N=2 | Consultant clinicians  The virtual clinic using the My Clinical Outcomes (MCO) web-based system, at the intervals set down by the BOA (British Orthopaedic Association) | **Consultant views:**   - Increase follow-up workload   Concerns regarding capacity and long-term management |
| Russell, 2011, Australia  Randomized controlled trial, Quantitative study (pre- and post-telerehabilitation treatment) | Physiotherapists  Low-bandwidth (18 kbit/s) PC-based telerehabilitation system to enable  rehabilitation services delivered directly to the home via an ordinary telephone (PSTN) line. | **Therapist experience:**   1. Delivery of effective and timely treatment 2. Key factor- easy integration into clinical practice for a successful telemedicine application. Software interface was easy and intuitive to use   Confident in the objective measurements collected with the system |
| Van Kasteren, 2018, Australia[276]  Qualitative study using focus group discussions & semi-structured interviews, N=23 | Surgeons, physiotherapists, general practitioners, research fellows  Digital technology | **Communication from clinician to patient:**   - Interactions between clinicians and patients are time-poor but information-rich, - Patients do not recall the information or instructions given to them because of the technical and complex information - Digital technology added value through improved communication and information flow between clinicians and patients. |
| Visperas, 2021, North America[262]  Randomized clinical trial, Quantitative study,  n=200 | Care team (Surgeon, Physician Assistants, Nurse Practitioners, Nurses)  Stryker Performance Solutions JointCOACH is a web-based Interactive Patient-Provider Software Platform (IPSP) that enables joint replacement patients to communicate with their care team via computer or smartphone throughout their episode of care | **Satisfaction**   - 70% care team were satisfied with the use of the IPSP and 60% would recommend it |

*Abbreviations: KR=Knee Replacement, HR=Hip Replacement, OA=Osteoarthritis, IC=Integrated Care, PT=Physiotherapist*

Table S8 Patients and caregiver perspective on use of technology/telerehabilitation/mobile application for rehabilitation monitoring (N=36)

| **Author, Year, Study design and sample size** | **End users and technology used** | **Key themes on patient satisfaction, patient perception/experiences, caregiver perspectives** |
| --- | --- | --- |
| Boissy T, 2011, Tousignant,2011, Moffet,2017, North America[272, 277, 278]  Descriptive study embedded in Randomized Controlled Trial,  N=48 | Patients post-KR  Telerehabilitation platform of two H264 videoconferencing  CODECs (Tandberg 550 MXP) with integrated wide-angle view cameras, remote-controlled pan, tilt zoom (PTZ), 20-inch liquid crystal display (LCD) screens and a dedicated modular software interface for user-friendly control of videoconferencing connections, PTZ camera functions, and external devices was used. | **Patient satisfaction and perception with in-home telerehabilitation:**   1. *Patients’ satisfaction with healthcare services:* High Satisfaction with the healthcare services provided was high for both the tele (86.0±15.2) and comparison groups (84.0±18.1) and no significant difference was observed between the two groups (p=0.721). 2. *Telehealth perception change:* Patient’s perception of in-home tele treatment was positive when the equipment was installed (81.2%±7.2%) as well as when it was removed (83.4%±8.3%). This difference was not statistically significant (p = 0.118). 3. Satisfaction level of both groups (in-home telerehabilitation) (TELE) and face-to-face home visit (STD) rehabilitation) did not differ and was very high (over 85%). The mean satisfaction score for each question ranged between 3.2 and 3.8 for the STD group and between 3.1 and 3.8 for the TELE group.   **Technical support need and service delivery reliability:**  Installation of a new Internet connection was required in 75% of the participants and average technician’s time to install test and uninstall technology (including travel time) was 308.4 min.  Remote technical support was solicited in 43% of the sessions (interventions were less than 3-min duration).  **Performance and use of network connection:**  Connection interruption per session occurred at least once in 21% of the telerehabilitation sessions delivered. Technical support was required in 43% of the sessions (before, during, or after), but the time of these interventions was about 3 min (2.8 – 6.1min). |
| Booth, 2023, North America,[279]  Single-centre retrospective cohort study, n=124 | TKA and UKA patients  Wearable remote monitoring technologies and smartphone app | **Satisfaction**   - 92.6% of the patients found the remote monitoring system easy to use - 94.5% patients felt the technologies motivated them during their postoperative recovery - 81.9% patients strongly felt that technologies allowed the surgeon to monitor more closely - 94.5% of them would recommend these technologies to other patients   **Perception**  Technology assisted consultation can supplement clinical visits but could not replace it |
| Cooper, 2022, UK[280]  Prospective, observational, single-arm feasibility study, n=17 | Patients with UKR and TKR  Remote monitoring device includes-wearable sensor, smartphone app, and dashboard. | **Technology**   - 94% found easy to download the app - 81% liked using the ROM sensor - 88% felt ROM sensor motivated to perform exercises   64% felt secure with the remote monitoring and telecommunication |
| Correia, 2019, Europe[168]  Non-randomized controlled trial, Quantitative  N=30 | Patient post TKA  Inertial Motion Sensors for real time biofeedback & remote monitoring of progress | **Satisfaction**   - 27 patients rated satisfaction with app as 10/10   **Usability**  60% required some sort of assistance for placement of sensors or interacting with app |
| Culliton, 2018, North America[281]  Randomized controlled trial,  N=335 | Patients undergoing TKR  e-learning tool with a thirty-one-page hard copy of ‘My Guide to Total Knee Joint Replacement’ was also to the intervention group. The control group was provided only with the guide. | **Patient expectations and satisfaction pre-operatively:**   - Expected TKA would substantially reduce their pain (83% intervention group, 84% control group), allow them to return to activities of daily living (78% intervention group, 76% control group), and improve their participation in sports, leisure or recreational activities (74% intervention group, 65% control group). - Preoperative patient satisfaction was low, with few patients satisfied with their present state prior to TKR (14% intervention group, 11% control group).   **Patient expectations and satisfaction post-operatively:**   - At one year postoperative, the proportion of patients whose expectations were not met was 21.8% in the control group and 21.4% in the intervention group   The proportion of patients who were satisfied with the TKA at one year postoperative was similar in the intervention 78.6% and control group 78.2% |
| Eichler, 2019, Europe[177]  Randomized controlled trial, Quantitative,  N=48 | Patients post TKA  Computer based telerehabilitation with sensor following virtual reality | **Acceptability and satisfaction**   - Easy to use and learn - Highly satisfied - Ready to use in future |
| Giunta, 2022, North America[282]  Prospective cross-sectional survey study, n=163 total joint arthroplasty patients (joint is not mentioned) | Joint arthroplasty patients  Telemedicine using smartphone, laptop, tablet, desktop computer and landline telephone | **Satisfaction**   - 74.6% patients found the technology easy to use - 80.2% were satisfied with the telemedicine visits   **Preference for in-person visit vs telemedicine**  Most of the patients preferred in-person visits as the first visit |
| Glinkowski, 2010, Europe[283]  Observational prospective cohort design, N=54 | Patients pre and post-KR  Patients’ attitudes toward telerehabilitation over the Internet at the beginning of practical implementation of telerehabilitation platform | **Patient’s attitudes towards internet use, computer skills, email use and telerehabilitation:**  5 patients declared good skills, one excellent and three sufficient. Nine patients use the Internet but only three of them do it daily. Emailing was confirmed only by 5 patients (9,43%). Fortunately, more patients presented positive attitude towards telerehabilitation. Willingness to attend telerehabilitation by 23 patients (41,5%). Computer skills, emailing and Internet use were found as independently influencing on willingness to attend telerehabilitation in the study group. |
| Grant, 2019, UK[284]  Qualitative study using in-depth interviews  N=13 | Patients undergoing TJR  Interview topic guide: (Ruhl, 2004)  Sensor Platform of Healthcare in a Residential  Environment (SPHERE) system comprises a group of low-power sensors that can continuously measure anonymised, time-stamped | **Three main themes relating to acceptability**  **of home monitoring technology:**   1. *Installation of ‘home-sensing technology’ on the journey to surgery*: many felt the study information booklet and the SPHERE user guide were useful to refer to. Some people found information about how to use the system hard to recall or to act on. Patients benefits appeared to be a primary motivator to allow this technology to be fitted and to monitor their activity within the home. 2. *The home space and defining unobtrusiveness:* Participants expressed concerns about internal damage to wall surfaces within the rooms. Suggested further improvements to the system, such as adding warning lights on the wristband or for warnings to be indicated clearly on the study information. Some participants found the wristband inconvenient and sometimes took it off because of this. Some participants described their concern that the system was capturing information about incorrect performance of exercises or other aspects of postoperative recovery.   *The pivotal role of social support networks:* household members felt encouraged at that a study would ‘monitor’ their health and outcomes outside of a hospital environment during this unfamiliar time. Informal social support networks encouraged patients to take part in the research. Friends, carers and family members ensured the system was working. Receiving an additional support and contact from a study research team before and after the operation served as reassurance. |
| Gunduz, 2021, Europe[188]  Non-randomized control group intervention study, Quantititative,  n=80 | Patients undergoing TKA  Preoperative video-based pain training | **Satisfaction**   - 80% of the patients were very satisfied with the pain training video   65% were very satisfied with the pain training booklet |
| Haase, 2004, Europe[189]  Randomized controlled trial,  Mixed-methods,  N=68 | Patients post TKA  Computer aided multimedia training for remote physiotherapy and communication | **Acceptability**  92% patients would recommend this training to other patients with similar complaints |
| Hong, 2022, North America[193]  A retrospective observational feasibility study, n=53 | Patients with TKA  Feedback on smartphone-based digital musculoskeletal surgical care program | **Acceptability**   - 67% will recommend the program to a friend or colleague (net promoter score of 9 to10 out of 10)   **Satisfaction**  The intervention group reported higher satisfaction with their surgery and post-surgery experience |
| Joshi, 2022, North America[285]  Qualitative feasibility study, n=16 | Patients with TKA  Mobile application-based home assessment tool | **Technology**  *Satisfaction*   - Positive intention to use mobile technology for home assessment.   *Suggested tool features, content, and format for ease of use*   - An application with minimal clicks - Tutorial or video instruction to navigate through app - Guidelines about how to click pictures in a way that protects their privacy - Cost free tool without advertisements - Application without password - Information on most common barriers in the home, easy home modifications - Minimal use of text and more visuals or pictures - App embedded within healthcare portal will increase reliability   *Barriers*  Inability to transfer picture from one technology to other |
| Kairy, 2013, North America[286]  Embedded single case study design,  N=5 | Patients post-KR  VC system located in the participant’s home was connected remotely through highspeed internet to the health centre’s system where the physiotherapist was located. | **Six main themes emerged for patients’ perceptions on in-home telerehabilitation:**   1. Improving access to services 2. Developing a bond with their therapist while maintaining a sense of personal space 3. Complementing telerehabilitation with in-person visits 4. Providing standardized yet tailored and challenging exercise programs using telerehabilitation 5. Perceived ease-of-use of telerehabilitation equipment 6. Achieving an ongoing sense of Support   **Satisfaction**   - 91% participants were comfortable in using telecommunication - All the participants were satisfied with the telerehabilitation experience and physiotherapy treatment - 91% felt that services were as good as in person care   **Technology**  36% felt that sound and image were stable during telerehabilitation sessions, and the app was easy to use. |
| Kuether, 2019, North America[208]  Retrospective cohort, N=40 | Patients post TKA  VERA system for communication and physiotherapy exercises | **Satisfaction**  Patients were willing to recommend the program to others and scored 90.3 |
| Lebleu, 2023, Europe[210]  Prospective multi-centre longitudinal cohort study, n=87 | Patients with TKA  App-based human-supported digital monitoring and rehabilitation program | **Satisfaction**  High level of patient acceptance (patient promoter score 89%) |
| LeBrun, 2021, North America[287]  Cross sectional study,  N=74 | Patients with UKR, TKR and revision arthroplasty  Telemedicine using computer/phone/tablet during the COVID-19 pandemic. | **Patient satisfaction and experiences with telemedicine:**   - *Logistics of accessing telemedicine:* 74% had never used telemedicine before and 28% needed assistance from someone else with accessing the telemedicine visit. - *Patient satisfaction:* 96% participants reported being slightly to very satisfied with telemedicine, 4% were slightly to very dissatisfied. - *Other experiences:* 51% noticed a reduction in expenses using telemedicine versus normal outpatient care, 99% noted a decrease in travel costs, 17% noted a decrease in work costs, and 6% noted a decrease in medical costs. Compared with standard outpatient treatment, 78% had an experience that was good, excellent, or the best imaginable, while 19% noted that their experience was OK or poor. In all, 68% would consider continuing telemedicine in addition to outpatient treatment. - *Positive experiences with early telemedicine:* less anxiety and stress related to traveling (50%), feeling more at ease in a familiar environment (33%), a longer appointment time (10%), and the ability to assess postoperative home environment (8%).   *Negative experiences with early telemedicine:* addressing symptoms in the absence of an in-personal examination (17%), a decreased sense of interpersonal connection with the physician (12%), technical difficulties (9%), a shorter appointment time (4%), and an inability to obtain a full orthopaedic evaluation without a radiograph (2%). |
| Lee, 2016, Asia[288]  Mixed Methods Study  N=25 | Patients post-knee surgery  ***Hardware:*** Nintendo Wii, a force sensor called the Balance Board, and a 50 in. plasma display panel monitor.  ***Software:*** In NWFP (RVL-006, Nintendo of Korea), VR game | **Experience of flow:**   1. *Sessions in which participants experienced flow the most:* Table Tilt (n = 11), followed by Tightrope Walk (n = 4), Balance Bridge (n = 3), Warrior (n = 3), Ski Slalom (n = 2), Single-Leg Extension (n = 1), and Penguin Slide (n = 1).   *Reasons for greatest flow:* correct level of difficulty (n = 7), clear goals (n = 6), concentration (n = 5), enjoyment (n = 5), beneficial effects of exercise (n = 3), and immediate feedback (n = 1).   1. *Sessions in which participants experienced flow the least:*  Palm Tree (n = 7), followed by Single-Leg Extension (n = 4), Ski Slalom (n = 4), Tightrope Walk (n = 4), Balance Bridge (n = 3), Penguin Slide (n = 1), Warrior (n = 1), and Table Tilt (n = 1).   *Reasons for least flow:* inadequate level of difficulty (n =16), pain (n = 3), lack of concentration (n = 2), lack of enjoyment (n = 2), lack of a sense of unity with virtual characters (n =2), decreased physical function (n = 1), and unclear goals (n =1).  **Correlation between Flow State Scale-2 Scores and**  **Physical Dysfunction:** There was no significant correlation between total FSS-2 scores and knee pain severity, physical dysfunction, or age.  **Experience of physical treatment and intention of exercise adherence:** The majority (96%) of participants said that they would like to use the NWFP in future rehabilitation treatment. |
| Marsh, 2014, North America[289]  Descriptive study embedded in an RCT,  N=229 | Patients 12 months post TKA  A web-based method of conducting follow ups, including review of radiographs and patient symptoms, | **Satisfaction**   - Ninety-one patients in the usual-care group indicated that they were either extremely or very satisfied with the follow up process compared with 90 patients (75.6%) who were in the web-based group - Majority were satisfied with the web-based follow up (29% extremely satisfied, 37% very satisfied, 20% somewhat satisfied). - Reasons for dissatisfaction included length of time took to receive results, difficulty using the online database, inability to ask questions and receive immediate feedback, and ability to see their radiographs in person at their appointments.   **Preference for Web-based versus In-person Follow up**   - No difference in patient preference for follow up method - Forty-one patients (44.1%) preferred the web-based method, 36 patients (38.7%) preferred the usual clinic follow up, and 16 (17.2%) had no preference. - Main reasons for preferring web-based follow up were decreased travel 40%), no wait times (44%), ability to have radiographs in home-town (33%), and ability to complete follow up from home (29%).   Main reasons for preferring the usual clinic follow up were that they preferred to see the surgeon in person (43%) and to have their radiographs taken at their surgeon’s hospital (28%). |
| Massip, 2020, Europe[274]  Descriptive study embedded in Randomized Controlled Trial,  N=29 | Patients scheduled for TKA  mHealth-enabled Integrated Care model, including a self-management app for patients, a set of integrated sensors, and a web-based platform connecting professionals from different settings or usual care | **Patient’s perception of person-centeredness:** the perception of patient-centeredness was very high  **Patient’s perception of continuity of care**: patients in IC scored better than patients in usual care (usual care: mean NCQ G1-G5 score 3.7, SD 0.9; integrated care: mean NCQ G1-G5 score 4.0, SD 1.0; P=.04).  **Satisfaction with the IC platform in IC arm patients:**  overall NPS score of +45%. The mean SUS score for patients was 68 (SD 24).  **Key factors for better perceptions:**   1. Comprehensive set of features including integration of other monitoring devices 2. The involvement of patients since early phases of development 3. The flexibility of potential end users, as the app could be managed directly by the patients 4. Avoided unnecessary visits to primary care centers or hospital 5. Appropriate feedback on the daily monitoring and patients’ achieved goals, 6. Push-up notifications to remind key events, tasks, or goals 7. Ease of use and quality-of-life features |
| McDonall, 2022 Australia[221]  Hybrid Type II Implementation study, n= 103 | Patients with KA  Multimedia intervention via iPad in the context of acute postoperative care | **Barriers were**   - **Structural barriers** – physical location of iPad in the ward for easy access - **Clinical-related barriers**- the negative comments by nurses about intervention can negatively affect confidence of patients to ask questions related to the program. - **Patient related factors**- being unable to watch the entire program because of sleepiness or tiredness, difficulty remembering to watch the program, and being too unwell to watch because of pain or other complications - **Technology related factors**- technical issues with the iPad having flat batteries.   **Concerns**   - Voiceover on the program was disturbing in the shared rooms   **Satisfaction and experience**   - 91.3% of patients found it easy to use   62.1% of patients could view the program as often as they wanted |
| Milliren, 2022 North America[225]  Quasi experimental design, n=1307 | Patients scheduled for and undergone KA  Impact of patient engagement tool consisting of sequenced digital prompts delivered via email or text message | **Engagement**  Older patients (65-79 years) were most likely to be engaged. |
| Nuevo, 2023, Europe[230]  Randomized controlled trial, Quantitative,  n=52 | Patients with TKA  Feedback on telerehabilitation (ReHub platform) that consists of wearable sensor, a web platform | **Usability and satisfaction**   - Satisfied with the telerehabilitation ( mean score 82.83 + 15.68 out of 100 in system usability scale) - Most of the patients were confident in using the system (9.46/10) - System was easy to use (9.13/10)   They ‘strongly disagree’ that system was cumbersome to use (1.09/10) |
| Onyeukwu, 2020, North America[231]  Randomized controlled trial, Mixed method  N=19 | Patients post TKA  Remote (wearable) rehabilitation monitoring platform (interACTION) for home exercise program | **Satisfaction**   - Majority of patients found the visual feedback easy to understand and forms of media (videos, text, motion feedback, etc.) useful - All patients said that they would consider using interACTION in the future.   **Technology**  *Affirmations-*   - Motivation/accountability - Visual feedback useful - Helpful when working   *Criticism*   - Calibration and sensor issues - System failure - Limited feedback   *Suggestion*   - Improve the user interface, harness/straps - Create better instructions   Make faster set-up |
| Parkes, 2019, UK[275]  Mixed Methods Study,  N=115 | Patients post-KR  The virtual clinic involved using the My Clinical Outcomes (MCO) web-based system, at the intervals set down by the BOA (British Orthopaedic Association) | **Patient views:**   - Virtual clinic pathway replaced a face-to-face outpatient appointment - Patients had confidence on receipt of the letter informing them the consultant had reviewed their scores and X-ray that the process was working. - PROMs were easy to understand but some frustrations in completing them. Some felt that they did not have a voice, the process was didactic, impersonal, and lacked flexibility. - Virtual clinic relatively easy to engage with and some found it helpful in self-management. - Time saving by reducing travelling distance, time taken off work and not having to wait to see a consultant in the outpatient department in addition to the availability of local X-ray seen in the questionnaire.   Comfortable and competent users of technology. |
| Pronk, 2020, Europe[240]  Randomized controlled trial, Quantitative,  N=38 | Patients post TKA  Pain coach app for education and recording of pain level | **Patients’ perception**   - 89% found app is easy to use - 79% found that app added value and would recommend to others |
| Ramkumar, 2019, North America[241]  Prospective cohort, N=25 | Post TKA patients  Remote monitoring system with real time biofeedback | **Satisfaction**   - Easy to use - Motivating and engaging due to facile user experience of the app, real time feedback, daily notifications - Ready to recommend   **Complaints-**  Low battery life of sleeve (36%) |
| Reid, 2021, North America[290]  Qualitative study using focus groups & phone interviews,  N=76 | Patients with Hip & Knee OA, Family caregivers  eHealth module | **Three themes emerged for patient and caregiver perceptions on eHealth modules:**   1. *“Easier to understand”:* there was a general agreement about the usefulness of written materials, images, and videos. For most of them, opinions regarding voiceovers were mixed and regarding quizzes were negative. 2. *“What does that mean”:* Participants spoke about the importance of emphasizing key information, providing clear rationale using simple language, and how the information needs to be relevant to their needs.   *“Preparation, right?”:* Participants identified the importance of having access to prehabilitation education within a time frame that allowed adequate time for review and consolidation of the information. |
| Russell, 2011, Australia[291]  Randomized controlled trial, Quantitative study (pre and post-telerehabilitation treatment), N=31 | Patients post-KR  Low-bandwidth (18 kbit/s) PC-based telerehabilitation system to enable  rehabilitation services to be delivered directly to the home via an ordinary telephone (PSTN) line. | **Patient experience**   - High ratings (>9/10) were recorded by participants on the *satisfaction questionnaire*. - High ratings (>7/10) were also observed for the *visual and auditory components* of the videoconference.   *Computer literacy questionnaires* completed by participants revealed that only 29% of them had ever used a computer. Of those 29%, the average self-rated confidence in being able to operate a computer was 0.8 (SD 2.1) out of 10.  **Patient satisfaction**   - High level of satisfaction due to easy-to-use graphical interface, better audio quality, and lack of technical difficulties; however visual quality was low - High satisfaction due to clear audio and method; low satisfaction due to lack of visual clarity   Wound recommend it to others |
| Scheper, 2019, Europe[244]  Prospective cohort, N=69 | Patient post TKA  Wound care app | **Patient’s perception**   - Easy to use and useful app   **Satisfaction**  Highly satisfied (8.2) on a scale from 0 to 10 |
| Szots, 2015, Europe[251]  Qualitative study as a part of a RCT using case reports and semi-structured interviews,  N=10 | Patients post-KR  Telephone follow up (TFUs) | **Three categories of patient’s views on TFUs:**   1. *A means for reflection and provision of adequate information and counselling:* seen as an opportunity to follow-up on issues arising during recovery, counselling, and communication 2. *Supportive:* appreciated as a signal of interest from the hospital, participants felt supported and listened. Also seen as a means of socialising or an opportunity to share their concerns. 3. *Not the only resource for support and counselling: Mode to* exchange of experience with newly operated and previously operated TKA patients, contact with the hospital, GP and PT |
| Van Kasteren, 2018, Australia[276]  Qualitative study using focus group discussions & semi-structured interviews, N=11 | Patients pre- and post-KR  Digital technology | **Communication From Patient to Clinician:**   - Add value to patient clinician communication by supporting patients in communicating how they are feeling and what they are experiencing. - Digital technology can assist in the following: Enhance the quality and frequency of patient communication with their care team using self-monitoring tools and wearables and assist in the early detection of postoperative complications and reduce patient anxiety. |
| Visperas, 2021, North America[262]  Randomized clinical trial, Quantitative,  N=200 | Patient pre TKA  Stryker Performance Solutions JointCOACH is a web based IPSP that enables joint replacement patients to communicate with their care team via computer or smartphone throughout their episode of care | **Satisfaction**   - 72% recommended IPSP platform |
| Wang Q, 2023, Asia[292]  Randomized controlled trial with Qualitative descriptive study, n=10 | Patients with TKA  Mobile app-based rehabilitation programme | **Five themes emerged for patient perceptions on telerehabilitation**   1. *Improved access to health care-* all participants described that it improved their access to health care by    - 1. Access to more comprehensive and reliable information      2. Better access to healthcare professionals      3. Convenient and flexible access to rehabilitation. 2. *Encouraged post*-*operative* recovery- 65%of participants    - 1. Increased engagement in recovery*-* the discussion forum, reminder messages that were sent to them weekly, timely communication with healthcare professionals engaged them more in the management of postoperative recovery.      2. Increased motivation in rehabilitation exercise- exercise demonstration videos, support from healthcare professionals and other patients motivated to become more active in rehabilitation exercises.      3. Enhanced confidence for rehabilitation- mobile application enabled them to undertake rehabilitation more independently. 3. *Established supportive relationships*- group chat helped to establish (84%) supportive relationship with healthcare professionals and other patients in the program. 4. *Facilitated learning*- facilitated learning of rehabilitation instructions (24%) by improving understanding and memorisation. 5. *Recommendations*    - 1. 24% of participants had recommended the app to the friends and family who had undergone similar surgeries.      2. Suggested programme improvement- to develop a frequently-asked-question section and regularly updating it in the app, scheduled discussion sessions with health care professionals instead of being on standby 24/7, to include health information and examples related to postoperative pain management, and user app manual in video format within the app.   **Four themes emerged for patient expectations from telerehabilitation**   1. *Assistance for self-management of rehabilitation -* all participants described the need for assistance at home for better management of postoperative rehabilitation.    - 1. Explicit goals and individualised guidance for rehabilitation- explicit goals to be set after discussion with healthcare professionals for better adherence to rehabilitation, individualised guidance via app to meet the health needs in different age groups and with different expectations for recovery.      2. Accessible information support- need for providing information via mobile app description of rehabilitation exercises, advice on how to do activities in daily life, precautions after joint replacement, visual presentation for rehabilitation exercises and instructions such as videos with commentary and subtitles, and to avoid medical jargons and abstractive symbols, methods to promote postoperative recovery, dietary advices, process of recovery and, traditional Chinese medicine to alleviate muscle tension and pain post operatively.      3. User-friendly platform for learning- easy operation and should be tested on various operating systems for reliability. 2. *Facilitating peer support*- participants expressed the desire to have peer support for learning and support. 3. *Facilitating contacts with healthcare professionals*- desire to have contact and reassurance from healthcare professionals via mobile app. 4. *Supporting emotional well*-*being*- provide adequate knowledge and skills to support postoperative emotional well-being. |
| Williams, 2022, UK[293]  Mixed method study, n=64 | Patients with TKA  Feedback on hybrid model of consultation (virtual consultations + pre-recorded rehabilitation content) | **Satisfaction**   - They valued empathetic communication, condition/person-specific support, motivation, and expert guidance - The pre-recorded programme was considered advantageous in relation to ease of access   **Barriers/areas for potential refinement**   - Connectivity for virtual consultations   Concerns regarding the virtual assessment of joint swelling, wounds, and gait generated perceptions of clinical uncertainty |
| Zhang X, 2021, Asia[267]  Non-randomized controlled trial, Quantitative, N=2292 | Patients post TKA  WeChat App for communication and education | **Satisfaction**   - Higher satisfaction among the patients who were more active in WeChat app compared to inactive patients |

***Abbreviations:*** *KR=Knee Replacement, TKR=Total Knee Replacement, HR=Hip Replacement, OA=Osteoarthritis, IC=Integrated Care, PT=Physiotherapist, KOOS= Knee Injury and Osteoarthritis Outcome Score. WOMAC=Western Ontario and McMaster Universities Osteoarthritis Index, ROM=Range of Motion, VR=Virtual Reality, NWFP= Nintendo Wii Fit Plus, TFU=Telephone Follow Up, FSS-2= Flow State Scale-2, VERA=Virtual Exercise Rehabilitation Assistant*

# References

1. Abdeen A, Monarrez R, Drew JM, Kennedy KF. Use of a Smart-Phone Mobile Application is Associated With Improved Compliance and Reduced Length of Stay in Patients Undergoing Primary Total Joint Arthroplasty of the Hip and Knee. The Journal of arthroplasty. 2022 Aug;37(8):1534-40. PMID: 35341922. doi: 10.1016/j.arth.2022.03.068.

2. Agostini M, Tonin P, Moja L, Banzi R, Pistotti V, Venneri A, et al. Telerehabilitation and recovery of motor function: a systematic review and meta-analysis. Journal of telemedicine and telecare. 2015 2015;21(4):202-13. PMID: rayyan-230149942.

3. Ahmad NA, Mat Ludin AF, Shahar S, Mohd Noah SA, Mohd Tohit N. Willingness, perceived barriers and motivators in adopting mobile applications for health-related interventions among older adults: a scoping review protocol. BMJ open. 2020 Mar 16;10(3):e033870. PMID: 32184309. doi: 10.1136/bmjopen-2019-033870.

4. Aksoy CC, Saracoglu I, Afsar E, Taspinar B, Taspinar F. The effectiveness of Nintendo Wii exercises in rehabilitation following knee surgery. Fizyoterapi Rehabilitasyon. 2017 2017;28(2):S88-S9. PMID: rayyan-230149470.

5. Alexandre DJA, Ramalho GS, Civile VT, Carvas Junior N, Cury Fernandes MB, Cacione DG, et al. Telerehabilitation versus conventional face‐to‐face land‐based exercises following hip or knee arthroplasty. Cochrane Database of Systematic Reviews. 2021 (11). PMID: CD014931. doi: 10.1002/14651858.CD014931.

6. Allsop S, Fairhall R, Morphet J. The impact of pre-operative telephone support and education on symptoms of anxiety, depression, pain and quality of life post total knee replacement: An exploratory case study. International journal of orthopaedic and trauma nursing. 2019 2019--;34(101528681):21-7. PMID: rayyan-230150803.

7. Al-Rub ZA, Hussaini M, Gerrand CH. What do patients know about their joint replacement implants? Scottish Medical Journal. 2014 2014;59(3):158-61. PMID: rayyan-230150010.

8. Anthony CA, Rojas E, Glass N, Keffala V, Noiseux N, Elkins J, et al. A Psycholgical Intervention Delivered by Automated Mobile Phone Messaging Stabilized Hip and Knee Function During the COVID-19 Pandemic: A Randomized Controlled Trial. The Journal of arthroplasty. 2022 Mar;37(3):431-7 e3. PMID: 34906660. doi: 10.1016/j.arth.2021.12.006.

9. Azhari A, Parsa A. Covid-19 outbreak highlights: Importance of home- based rehabilitation in orthopedic surgery. Archives of Bone and Joint Surgery. 2020 2020;8:317-8. PMID: rayyan-230148861.

10. Bahadori S, Wainwright TW, Ahmed OH. Smartphone apps for total hip replacement and total knee replacement surgery patients: a systematic review. Disability and rehabilitation. 2020;42(7):983-8. doi: <http://dx.doi.org/10.1080/09638288.2018.1514661>.

11. Baker K, LaValley MP, Brown C, Felson DT, Ledingham A, Keysor JJ. Efficacy of Computer-Based Telephone Counseling on Long-Term Adherence to Strength Training in Elderly Patients With Knee Osteoarthritis: A Randomized Trial. Arthritis care & research. 2020;72(7):982-90. doi: <https://dx.doi.org/10.1002/acr.23921>.

12. Baxter SN, Johnson AH, Brennan JC, Dolle SS, Turcotte JJ, King PJ. The Efficacy of Telemedicine Versus In-Person Education for High-Risk Patients Undergoing Primary Total Joint Arthroplasty. The Journal of arthroplasty. 2023 Jul;38(7):1230-7 e1. PMID: 36690187. doi: 10.1016/j.arth.2023.01.015.

13. Bell KM, McClincy MP, Onyeukwu C, Hartman R, Merill Z, Mukherjee A, et al. Feasibility and usability of a portable system for monitoring knee motion during physical rehabilitation. Journal of Orthopaedic Research. 2017;35(Supplement 1).

14. Bentley J, Searle D, Walmsley K, Patel N, Toms A, Phillips J. AN EVALUATION OF THE VIRTUAL KNEE CLINIC POST TOTAL KNEE ARTHROPLASTY. Knee. 2020 2020;27:S21-S2. PMID: rayyan-230148759.

15. Berton A, Longo UG, Candela V, Denaro V, Fioravanti S, Giannone L, et al. Virtual reality, augmented reality, gamification, and telerehabilitation: Psychological impact on orthopedic patients' rehabilitation. Journal of Clinical Medicine. 2020 2020;9(8):1-13. PMID: rayyan-230148444.

16. Bini SA, Shah RF, Bendich I, Patterson JT, Hwang KM, Zaid MB. Machine Learning Algorithms Can Use Wearable Sensor Data to Accurately Predict Six-Week Patient-Reported Outcome Scores Following Joint Replacement in a Prospective Trial. Journal of Arthroplasty. 2019 2019;34(10):2242-7. PMID: rayyan-230151659.

17. Blasco J, Blasco M, Igual-Camacho C, Anton-Anton V, Ortiz-Llueca L, Roig-Casasus S. The efficacy of virtual reality tools for total knee replacement rehabilitation: A systematic review. Physiotherapy theory and practice. 2021 2021;37(6):682-92. PMID: rayyan-230148577.

18. Boekesteijn R, Smolders J, Busch V, Keijsers N, Geurts A, Smulders K. Objective monitoring of functional recovery after total knee and hip arthroplasty using sensor-derived gait measures. PeerJ. 2022;10:e14054. PMID: 36193431. doi: 10.7717/peerj.14054.

19. Bogue E, Twiggs J, Liu D. Prehabilitation using a novel, mobile application reduces length of stay in patients undergoing primary total knee arthroplasty. Journal of Orthopaedic Research. 2017 2017;35. PMID: rayyan-230149575.

20. Browne JA. Leveraging Early Discharge and Telehealth Technology to Safely Conserve Resources and Minimize Personal Contact During COVID-19 in an Arthroplasty Practice. Journal of Arthroplasty. 2021 2021;36(7):S52-S5. PMID: rayyan-230148472.

21. Bruyere O. Connected devices in musculoskeletal health. Osteoporosis International. 2017 2017;28(1):S89. PMID: rayyan-230149539.

22. Buvanendran A, Sremac AC, Merriman PA, McCarthy RJ, Della Valle CJ, Burns JWA-M, Robert J., et al. Preoperative cognitive-behavioral therapy for reducing pain catastrophizing and improving pain outcomes after total knee replacement: A randomized clinical trial. Regional Anesthesia and Pain Medicine. 2021 2021;46(4):313-21. PMID: rayyan-230148672.

23. Byra J, Czernicki K. The effectiveness of virtual reality rehabilitation in patients with knee and hip osteoarthritis. Journal of Clinical Medicine. 2020 2020;9(8):1-18. PMID: rayyan-230148441.

24. Cankaya D, Akti S, Unal SB, Sezgin EA. Unicompartmental knee arthroplasty results in a better gait pattern than total knee arthroplasty: Gait analysis with a smartphone application. Jt Dis Relat Surg. 2021;32(1):22-7. PMID: 33463414. doi: 10.5606/ehc.2021.79635.

25. Chaudhry H, Nadeem S, Mundi R. How Satisfied Are Patients and Surgeons with Telemedicine in Orthopaedic Care During the COVID-19 Pandemic? A Systematic Review and Meta-analysis. Clinical orthopaedics and related research. 2021 2021;479(1):47-56. PMID: rayyan-230148758.

26. Chen M, Li P, Lin F. Influence of structured telephone follow-up on patient compliance with rehabilitation after total knee arthroplasty. Patient Preference and Adherence. 2016 2016;10:257-64. PMID: rayyan-230149679.

27. Chughtai M, Piuzzi N, Yakubek G, Khlopas A, Sodhi N, Sultan AA, et al. Use of an App-Controlled Neuromuscular Electrical Stimulation System for Improved Self-Management of Knee Conditions and Reduced Costs. Surg Technol Int. 2017 Oct 12;31:221-6. PMID: 29044455.

28. Coenen P, Smit DJM, Anema JR, Straat AC, Kuijer PPFM, Hulsegge G, et al. Development of a personalized m/ehealth algorithm for the resumption of activities of daily life including work and sport after total and unicompartmental knee arthroplasty: A multidisciplinary Delphi study. International Journal of Environmental Research and Public Health. 2020 2020;17(14):1-15. PMID: rayyan-230148971.

29. Cooper RA, Cooper R. Rehabilitation Engineering: A perspective on the past 40-years and thoughts for the future. Medical Engineering and Physics. 2019 2019;72:3-12. PMID: rayyan-230148420.

30. Dahlberg LE, Ignjatovic MM, Nero H, Gorely T, Beastall J, Gibson A, et al. Preliminary Evaluation Of A Digital Therapeutic For Osteoarthritis Patients Awaiting Knee Or Hip Surgery, In Remote And Rural Communities. Osteoarthritis and Cartilage. 2023;31(Supplement 1):S387. PMID: 2023243178. doi: <https://dx.doi.org/10.1016/j.joca.2023.01.443>.

31. De Faoite D. The advantages of electronic patient-reported measures and an example digital platform to collect ePROs after total knee arthroplasty. Medicine Access at Point of Care. 2018 2018;2. PMID: rayyan-230149384.

32. Denecke K, Kyburz P, Gfeller S, Deng Y, Burkle T. A Concept for Improving Cross-Sector Care by a Mobile Patient Navigator App. Studies in health technology and informatics. 2018 2018;255:160-4. PMID: rayyan-230149215.

33. Duong V, Dennis S, Ferreira ML, Heller G, Nicolson P, Robbins S, et al. Predictors of adherence to a digital physcial intervention following total knee replacement. Osteoarthritis and Cartilage. 2022;30(Supplement 1):S73-S4. PMID: 2017443172. doi: <https://dx.doi.org/10.1016/j.joca.2022.02.088>.

34. El Ashmawy A-AH, Dowson K, El-Bakoury A, Hosny HAH, Yarlagadda R, Keenan JA-EA, Abd-Allah H., et al. Effectiveness, Patient Satisfaction, and Cost Reduction of Virtual Joint Replacement Clinic Follow-Up of Hip and Knee Arthroplasty. Journal of Arthroplasty. 2021 2021;36(3):816. PMID: rayyan-230148698.

35. Fabres Martin C, Ventura Parellada C, Herrero Anton de Vez H, Ordonez Urgiles CE, Alonso-Rodriguez Piedra J, Mora Guix JM. Telemedicine approach for patient follow-up after total knee and reverse total shoulder arthroplasty: a pilot study. International Journal of Computer Assisted Radiology and Surgery. 2023;18(3):595-602. PMID: 2020227674. doi: <https://dx.doi.org/10.1007/s11548-022-02784-z>.

36. Fennema MC, Bloomfield RA, Lanting BA, Birmingham TB, Teeter MG. Repeatability of measuring knee flexion angles with wearable inertial sensors. Knee. 2019 2019;26(1):97-105. PMID: rayyan-230151689.

37. Ferguson-Pell M, Armstrong E. Patient and Clinician Reported Outcomes and Experiences Following Advanced Tele-Rehabilitation Assessments Conducted in Rural Alberta. Archives of Physical Medicine and Rehabilitation. 2021 2021;102(10):e10. PMID: rayyan-230148398.

38. Ferrara PE, Codazza S, Ricciardi D, Ronconi G, Ferriero G, Foti C, et al. The effectiveness of telerehabilitation after hip or knee arthroplasty: A narrative review. Journal of Biological Regulators and Homeostatic Agents. 2020 2020;34(5):75-9. PMID: rayyan-230148447.

39. Ferriero G, Vercelli S, Sartorio F, Foti C. Accelerometer- and photographic-based smartphone applications for measuring joint angle: Are they reliable? Journal of Arthroplasty. 2014 2014;29(2):448-9. PMID: rayyan-230150004.

40. Freiman S, Schwabe MT, Barrack RL, Nunley RM, Clohisy JC, Lawrie CM. Telemedicine for patients undergoing arthroplasty : access, ability, and preference. Bone Joint J. 2021 Jul;103-b(7 Supple B):98-102. PMID: 34192903. doi: 10.1302/0301-620x.103b7.Bjj-2020-2420.R1.

41. Fuchs L, Kluska A, Novak D, Kosashvili Y. The influence of early virtual reality intervention on pain, anxiety, and function following primary total knee arthroplasty. Complementary therapies in clinical practice. 2022;49:101687. PMID: 639482171. doi: <https://dx.doi.org/10.1016/j.ctcp.2022.101687>.

42. Fung V, Gomez M, Shaffer J, Chung E, Ho A. The utilization of nintendo wii fittm in the rehabilitation of outpatients following total knee replacements-a randomized controlled trial. Physiotherapy (United Kingdom). 2011 2011;97:eS419. PMID: rayyan-230150338.

43. Fusco F, Turchetti G. Telerehabilitation after total knee replacement: Preliminary cost-utility analysis of an innovative device. Value in Health. 2015 2015;18(7):A648-A9. PMID: rayyan-230149906.

44. Fusco F, Turchetti G. A cost-effectiveness analysis for total knee arthroplasty telerehabilitation: Proof of concept of a decision model. Value in Health. 2014 2014;17(7):A380. PMID: rayyan-230150038.

45. Fusco F, Turchetti G. Interactive business models for telerehabilitation after total knee replacement: Preliminary results from Tuscany2015 2015. 502-11 p.

46. Gakhar H, McConnell B, Apostolopoulos AP, Lewis P. A pilot study investigating the use of at-home, web-based questionnaires compiling patient-reported outcome measures following total hip and knee replacement surgeries. Journal of long-term effects of medical implants. 2013 2013;23(1):39-43. PMID: rayyan-230151084.

47. Gilbert AW, Jaggi A, May CR. What is the patient acceptability of real time 1:1 videoconferencing in an orthopaedics setting? A systematic review. Physiotherapy (United Kingdom). 2018;104(2):178-86. doi: <http://dx.doi.org/10.1016/j.physio.2017.11.217>.

48. Gollish JD, Pereira L, MacLeod AM, Wainwright A, Kennedy D, Robarts S, et al. myHip&Knee: Improving Patient Engagement and Self-Management Through Mobile Technology. Healthc Q. 2019 Jul;22(2):63-7. PMID: 31556382. doi: 10.12927/hcq.2019.25902.

49. Gonzalez Ruiz C, Gonzalez Garcia D, Rocha Palacios V, Romero Candel P, Diez Sanchez B, Corral Brito S, et al. One year after e-health: attention through videoconference in the postoperative of patients intervened for total knee prosthesis (TKP) or HIP (THP). International Journal of Clinical Practice. 2019.

50. Grant S, Whitehouse MR, Blom AW, Judge A, Craddock I, Gooberman-Hill R. Using home sensing technology to assess outcome and recovery after joint replacement e findings from the hip and knee study of a sensor platform of healthcare in a residential environment. Osteoarthritis and Cartilage. 2018 2018;26:S342-S3. PMID: rayyan-230149373.

51. Grant S, Judge A, Tonkin EL, Blom AW, Whitehouse MR, Gooberman-Hill R, et al. Using home sensing technology to assess outcome and recovery after hip and knee replacement in the UK: The HEmiSPHERE study protocol. BMJ open. 2018 2018;8(7):e021862. PMID: rayyan-230149222.

52. Gumaa M, Rehan Youssef A. Is Virtual Reality Effective in Orthopedic Rehabilitation? A Systematic Review and Meta-Analysis. Physical therapy. 2019 2019;99(10):1304-25. PMID: rayyan-230148913.

53. Gwam CU, Urquico KB, Etcheson JI, George NE, Delanois RE, Higuera Rueda CAA-D, Ronald E., et al. Use of new interactive patient-provider software improves patient satisfaction and outcomes-a retrospective single-center study. Arthroplasty Today. 2019 2019;5(1):73-7. PMID: rayyan-230149094.

54. Hadley C, McGrath M, Brahmabhatt S, Mazur DW, Prodoehl JP, Cohen SB, et al. Comparison of traditional physical therapy to internet-based physical therapy after knee arthroscopy: A prospective randomized controlled trial comparing patient outcomes and satisfaction. Orthopaedic Journal of Sports Medicine. 2019 2019;7(7). PMID: rayyan-230149146.

55. Hallfors E, Saku SA, Makinen TJ, Madanat R. A Consultation Phone Service for Patients With Total Joint Arthroplasty May Reduce Unnecessary Emergency Department Visits. Journal of Arthroplasty. 2018;33(3):650-4. doi: <http://dx.doi.org/10.1016/j.arth.2017.10.040>.

56. Han S-L, Xie M-J, Chien C-C, Cheng Y-C, Tsao C-W. Using MEMS-based inertial sensor with ankle foot orthosis for telerehabilitation and its clinical evaluation in brain injuries and total knee replacement patients. Microsystem Technologies. 2016 2016;22(3):625-34. PMID: rayyan-230151745.

57. Naeemabadi M SJ, Klastrup A, , Schlünsen AP, Lauritsen REK, Hansen J, Madsen NK, Simonsen O, et al. Development of an individualized asynchronous sensor-based telerehabilitation program for patients undergoing total knee replacement: Participatory design. Health informatics journal. 2020 2020;26(4):2492-511. PMID: rayyan-230148481.

58. Hardt S, Wassilew G, Schulz MRG, Liodakis E, Pfitzner T, Horstmann H, et al. Improved early outcome after TKA through an app-based active muscle training programme-a randomized-controlled trial. Knee surgery, sports traumatology, arthroscopy : official journal of the ESSKA. 2018 2018;26(11):3429-37. PMID: rayyan-230149219.

59. Ho A, Shaffer J, Gomez M, Fung V. The utilization of nintendo wii fit in the rehabilitation of outpatients following total knee replacements: Preliminary results of a randomized controlled trial. Archives of Physical Medicine and Rehabilitation. 2010 2010;91(10):e37. PMID: rayyan-230150435.

60. Holmes M, Song H, Tonkin E, Nieto MP, Grant S, Flach P. Analysis of patient domestic activity in recovery from hip or knee replacement surgery: Modelling wrist-worn wearable RSSI and accelerometer data in the wild2018 2018. 13-20 p.

61. Huang S, Kuo M-L, Yu H-M, Huang C-H, Shieh W-Y, Hsu W-H, et al. Clinical information and guidance shared via a patient infotainment system can reduce hospital stay and maintain 2 medical quality for total knee arthroplasty: A single-blinded quasi-randomised controlled trial. International journal of nursing studies. 2020 2020;104:103440. PMID: rayyan-230148686.

62. Jansson M, Koivisto J, Pikkarainen M. Identified opportunities for gamification in the elective primary fast-track total hip and knee arthroplasty journey: Secondary analysis of healthcare professionals' interviews. Journal of Clinical Nursing. 2020;29(13-14):2338-51. doi: <http://dx.doi.org/10.1111/jocn.15246>.

63. Jansson J, Laukka E, Kanste O, Koivisto J, Jansson M. Identified gamification opportunities for digital patient journey solution during an arthroplasty journey: secondary analysis of patients' interviews. Nursing open. 2022;9(4):2044-53. PMID: 637849070. doi: <https://dx.doi.org/10.1002/nop2.1215>.

64. Jayakumar P, Uhler LM, Koenig KM, Aksan N, Rathouz PJ, Bozic KJ, et al. Comparison of an Artificial Intelligence-Enabled Patient Decision Aid vs Educational Material on Decision Quality, Shared Decision-Making, Patient Experience, and Functional Outcomes in Adults with Knee Osteoarthritis: A Randomized Clinical Trial. JAMA Network Open. 2021 2021;4(2):e2037107. PMID: rayyan-230148570.

65. Jetanalin Pim, Kim Hyeon Eui, Agha Zia, Heintzman Nathaniel, Ohno-Macado Lucila, J. LS. Feasibility of Remote Activity and Functional Status Monitoring of Patients with Hip or Knee Pain. American College of Rheumatology Empowering Rheumatology Professionals2012.

66. Jiang S, Xiang J, Gao X, Guo K, Liu B. The comparison of telerehabilitation and face-to-face rehabilitation after total knee arthroplasty: A systematic review and meta-analysis. Journal of telemedicine and telecare. 2018 2018;24(4):257-62. PMID: rayyan-230149393.

67. Kang K, Geng Q, Xu H, Zheng X, Dong J, Li T, et al. Clinical study of a new wearable device for rehabilitation after total knee arthroplasty. National Medical Journal of China. 2018 2018;98(15):1162-5. PMID: rayyan-230151705.

68. Karasavvidis T, Hirschmann MT, Kort NP, Terzidis I, Totlis T. Home-based management of knee osteoarthritis during COVID-19 pandemic: literature review and evidence-based recommendations. Journal of Experimental Orthopaedics. 2020;7(1):52. doi: <http://dx.doi.org/10.1186/s40634-020-00271-5>.

69. Kim K, Pham D, Schwarzkopf R. Mobile Application Use in Monitoring Patient Adherence to Perioperative Total Knee Arthroplasty Protocols. Surg Technol Int. 2016 Apr;28:253-60. PMID: 27042789.

70. Kocak UZ, Guran O, Kalkan S, Kaya E, Kurt M, Karatosun V, et al. Assessing the knee flexion range of motion after total knee arthroplasty: Technology versus senses. Journal of Bodywork and Movement Therapies. 2021 2021;28:547-51. PMID: rayyan-230148385.

71. Koutras C, Heep H, Bitsaki M, Koutras G, Nikolaou C. Socioeconomic impact of e-Health services in major joint replacement: A scoping review. Technology and Health Care. 2015 2015;23(6):809-17. PMID: rayyan-230149832.

72. Krumsvik OA, Babic A. Designing an E-Learning Platform for Postoperative Arthroplasty Adverse Events. Studies in health technology and informatics. 2017 2017;235:348-52. PMID: rayyan-230149600.

73. Kuan WYH, Takahashi T, Bourcet A, Crawford B. PMS17 Usability Testing of a Patient Engagement Digital Platform for TOTAL Knee Arthroplasty (TKA) Patients in JAPAN. Value in Health Regional Issues. 2020;22:S67-S8. doi: 10.1016/j.vhri.2020.07.356.

74. Kurtz SM, Higgs GB, Chen Z, Koshut WJ, Tarazi JM, Sherman AE, et al. Patient Perceptions of Wearable and Smartphone Technologies for Remote Outcome Monitoring in Total Knee Arthroplasties. Journal of Knee Surgery. 2022. PMID: 2020062178. doi: <https://dx.doi.org/10.1055/s-0042-1755378>.

75. Kwasnicki RM, Ali R, Jordan SJ, Atallah L, Leong JJ, Jones GG, et al. A wearable mobility assessment device for total knee replacement: A longitudinal feasibility study. Int J Surg. 2015 Jun;18:14-20. PMID: 25868424. doi: 10.1016/j.ijsu.2015.04.032.

76. Laursen M, Mikkelsen S, Pedersen PU, Vesterby MS, Larsen J, Soballe K, et al. Telemedicine support shortens length of stay after fast-track hip replacement: A randomized controlled trial. Acta Orthopaedica. 2017 2017;88(1):41-7. PMID: rayyan-230149624.

77. Leal-Blanquet J, Alentorn-Geli E, Gines-Cespedosa A, Martinez-Diaz S, Caceres E, Puig L. Effects of an educational audiovisual videodisc on patients' pre-operative expectations with total knee arthroplasty: a prospective randomized comparative study. Knee surgery, sports traumatology, arthroscopy : official journal of the ESSKA. 2013 2013--;21(11):2595-602. PMID: rayyan-230151111.

78. Li P, Li X, Meng H, Huang L, Zhang L, Wang S, et al. Video-Assisted Health Education Promotes Rehabilitation Training of Total Knee Arthroplasty Patients and Reduces Stress and Burnout in Nurses Compared to Oral Education. Biomed Res Int. 2021;2021:5058899. PMID: 2015441714. doi: <https://dx.doi.org/10.1155/2021/5058899>.

79. Lin H-T, Li Y-I, Hu W-P, Huang C-C, Du Y-C. A scoping review of the efficacy of virtual reality and exergaming on patients of musculoskeletal system disorder. Journal of Clinical Medicine. 2019;8(6):791. doi: <http://dx.doi.org/10.3390/jcm8060791>.

80. Linedale EC, Bills E, Dimopoulos A, Yeoh J, Nolan M, Hume V, et al. Development of a feasible and acceptable digital prehabilitation pathway to improve elective surgical outcomes. Frontiers in Digital Health. 2023;5:1054894. PMID: 2021708260. doi: <https://dx.doi.org/10.3389/fdgth.2023.1054894>.

81. Li L. Effect of Remote Control Augmented Reality Multimedia Technology for Postoperative Rehabilitation of Knee Joint Injury. Computational and mathematical methods in medicine. 2022;2022:9320063. doi: <https://dx.doi.org/10.1155/2022/9320063>.

82. Lopez-Olivo MA, Ingleshwar A, Volk R, Barbo A, Suarez-Almazor ME, Jibaja-Weiss M. Development of multimedia patient education tools (MM-PTET) for osteoarthritis (OA), osteoporosis (OP) and rheumatoid arthritis patients (RA). Arthritis and Rheumatology. 2014 2014;66:S882. PMID: rayyan-230150029.

83. Luna IE, Kehlet H, Aasvang EK, Peterson B. Individualized assessment of post-arthroplasty recovery by actigraphy: a methodology study. Journal of Clinical Monitoring and Computing. 2017 2017;31(6):1283-7. PMID: rayyan-230149439.

84. Lysack C, Dama M, Neufeld S, Andreassi E. A compliance and satisfaction with home exercise: a comparison of computer-assisted video instruction and routine rehabilitation practice. Journal of allied health. 2005 2005;34(2):76-82. PMID: rayyan-230151353.

85. Makino K, Ogura T, Nakamura M, Terada H. Development of a Switchable Wearable Robot for Rehabilitation After Surgery of Knee2021 2021. 60-7 p.

86. Marsh J, Bryant D, Hoch JS, MacDonald SJ, Naudie D, McCalden R, et al. Economic evaluation of web-based compared with in-person follow-up after total joint arthroplasty. Journal of Bone and Joint Surgery - American Volume. 2014 2014;96(22):1910-6. PMID: rayyan-230150008.

87. Marsh JD, Bryant DM, MacDonald SJ, Naudie DDR, McCalden RW, Howard JL, et al. Feasibility, effectiveness and costs associated with a web-based follow-up assessment following total joint arthroplasty. The Journal of arthroplasty. 2014 2014--;29(9):1723-8. PMID: rayyan-230151047.

88. Mateo KF. Effect of a smartphone app plus an accelerometer on physical activity and functional recovery during hospitalization after orthopedic surgery. Journal of Clinical Outcomes Management. 2020 2020;27(5):202-9. PMID: rayyan-230148817.

89. McGinnis RS, Patel S, Silva I, Mahadevan N, DiCristofaro S, Jortberg E, et al. Skin mounted accelerometer system for measuring knee range of motion. Conference proceedings : Annual International Conference of the IEEE Engineering in Medicine and Biology Society IEEE Engineering in Medicine and Biology Society Annual Conference. 2016 2016;2016:5298-302. PMID: rayyan-230149810.

90. Moffet H, Tousignant M, Boissy P, Corriveau H, Nadeau S, Merette C, et al. Evaluating the quality of an on-going clinical trial on the effectiveness of telerehabilitation service after knee arthroplasty: A one-year summary. Physiotherapy (United Kingdom). 2011 2011;97:eS821-eS2. PMID: rayyan-230150336.

91. Moore MR, Galetta MS, Schwarzkopf R, Slover JD. Patient Satisfaction and Interest in Telemedicine Visits Following Total Knee and Hip Replacement Surgery. Telemedicine journal and e-health : the official journal of the American Telemedicine Association. 2022;28(9):1309-16. PMID: 637169345. doi: <https://dx.doi.org/10.1089/tmj.2021.0439>.

92. Moore AJ, Wylde V, Bertram W, Beswick AD, Howells N, Gooberman-Hill R. Healthcare professionals' views on implementing the STAR care pathway for people with chronic pain after total knee replacement: A qualitative study. PLoS ONE. 2023;18(4 April):e0284406. PMID: 2024278932. doi: <https://dx.doi.org/10.1371/journal.pone.0284406>.

93. Mouli VH, Carrera CX, Schudrowitz N, Jay JF, Shah V, Fitz W. Post‐operative remote monitoring for same‐day discharge elective orthopedic surgery: A pilot study. Sensors. 2021 2021;21(17). PMID: rayyan-230151552.

94. Naidu Helm A. EE135 Cost Comparison of a Smartphone-Based Care Platform Versus Traditional Care in Total Knee Arthroplasty in the US: An Integrated Healthcare Delivery System Perspective. Value in Health. 2023;26(6 Supplement):S84. PMID: 2025049407. doi: <https://dx.doi.org/10.1016/j.jval.2023.03.438>.

95. Nelson M, Russell T, Bourke M, Crossley K, McPhail S. Cost-effectiveness of telerehabilitation versus traditional care after total hip replacement: A trial-based economic evaluation. Journal of telemedicine and telecare. 2021;27(6):359-66. doi: <http://dx.doi.org/10.1177/1357633X19869796>.

96. Nery ECHP, Neves R, Correia F, Molinos M, Tulha J, Seabra R, et al. Digital versus conventional rehabilitation after total knee arthroplasty: A long-term follow-up. European Geriatric Medicine. 2020 2020;11:S239. PMID: rayyan-230148702.

97. Nogueira JBS, Carvalho ACGDS, Barros Filho EMD, Araújo LHDC, Bezerra MJC, Leite JAD. Planning a total knee arthroplasty through an application for mobile devices: case report. Revista Brasileira de Ortopedia. 2018 2018;53(6):792-6. PMID: rayyan-230151694.

98. Oatis C, Elk JL-T, Rizk J, Benbow E, Zheng H, Li W, et al. A Web-Based Data Capture System Can Successfully Collect Detailed and Quantifiable Physical Therapy Intervention Data Post Total Knee Replacement. Arthritis and Rheumatology. 2020 2020;72:1185-6. PMID: rayyan-230148721.

99. Pastora-Bernal JM, Baron-Lopez FJ, Martin-Valero R, Estebanez-Perez MJ. Evidence of Benefit of Telerehabitation After Orthopedic Surgery: A Systematic Review. Journal of medical Internet research. 2017;19(4):e142. doi: <http://dx.doi.org/10.2196/jmir.6836>.

100. Patterson JT, Wu H-H, Chung CC, Bendich I, Barry JJ, Bini SAA-P, Joseph T., et al. Wearable activity sensors and early pain after total joint arthroplasty. Arthroplasty Today. 2020 2020;6(1):68-70. PMID: rayyan-230148883.

101. Penders A, Octavia JR, Lefevre S, Browaeys H, Lauwers G, Van Onsem M, et al. SEKO: Smart system for assisting home-based rehabilitation of knee arthroplasty patients2018 2018. 214-20 p.

102. Petersen W, Karpinski K, Backhaus L, Bierke S, Haner M. A systematic review about telemedicine in orthopedics. Archives of Orthopaedic and Trauma Surgery. 2021 2021;141(10):1731-9. PMID: rayyan-230148429.

103. Pfeufer D, Gililland J, Bocker W, Kammerlander C, Anderson M, Krahenbuhl N, et al. Training with biofeedback devices improves clinical outcome compared to usual care in patients with unilateral TKA: a systematic review. Knee Surg Sports Traumatol Arthrosc. 2019 May;27(5):1611-20. PMID: 30334150. doi: 10.1007/s00167-018-5217-7.

104. Pietschmann J, Geu Flores F, Jöllenbeck T. Gait Training in Orthopedic Rehabilitation after Joint Replacement-Back to Normal Gait with Sonification? International Journal of Computer Science in Sport. 2019 2019;18(2):34-48. PMID: rayyan-230151665.

105. Pila S, Stern BZ, Rothrock NE, Franklin PD. Evaluating a web-based personalized decision report for total knee or hip replacement: Lessons learned from patients. Journal of evaluation in clinical practice. 2023 2023;29(5):844-53. PMID: rayyan-536818949. doi: doi:<https://dx.doi.org/10.1111/jep.13887> PT - Randomized Controlled Trial, Journal Article, Research Support, Non-U.S. Gov't.

106. Preston N, McHugh GA, Hensor EMA, Grainger AJ, O’Connor PJ, Conaghan PG, et al. Developing a standardized approach to virtual clinic follow-up of hip and knee arthroplasty. Bone and Joint Journal. 2019 2019;101(8):951-9. PMID: rayyan-230151687.

107. Preston NJ, McHugh GA, Hensor E, Grainger AJ, O'Connor PJ, Conaghan PG, et al. Feasibility testing of a standardised virtual clinic for follow-up of patients after hip and knee arthroplasty. Annals of the Royal College of Surgeons of England. 2023;105(3):252-62. PMID: 638772032. doi: <https://dx.doi.org/10.1308/rcsann.2021.0356>.

108. Rantala A, Jansson MM, Miettunen J, Puhto A-P, Pikkarainen M. The effects and safety of telerehabilitation in patients with lower-limb joint replacement: A systematic review and narrative synthesis. Journal of telemedicine and telecare. 2020:1357633X20917868. doi: <http://dx.doi.org/10.1177/1357633X20917868>.

109. Rogante M, Grigioni M, Giacomozzi C, Cordella D. Ten years of telerehabilitation: A literature overview of technologies and clinical applications. NeuroRehabilitation. 2010 2010;27(4):287-304. PMID: rayyan-230150456.

110. Rognsvag T, Lindberg MF, Lerdal A, Stubberud J, Furnes O, Holm I, et al. Development of an internet-delivered cognitive behavioral therapy program for use in combination with exercise therapy and education by patients at increased risk of chronic pain following total knee arthroplasty. BMC health services research. 2021;21(1):1151. PMID: 636617730. doi: <https://dx.doi.org/10.1186/s12913-021-07177-7>.

111. Rognsvaag T, Bergvad I, Furnes O, Indrekvam K, Lerdal A, Lindberg M, et al. Feasibility Of A Multicenter Randomized Controlled Trial Of A Complex Intervention Of Combined Exercise Therapy, Education, Cognitive Behavioral Therapy And Total Knee Arthroplasty In Patients With Knee Osteoarthritis. Osteoarthritis and Cartilage. 2023;31(Supplement 1):S397-S8. PMID: 2023243122. doi: <https://dx.doi.org/10.1016/j.joca.2023.01.459>.

112. Rosner BI, Gottlieb M, Anderson WN. Effectiveness of an Automated Digital Remote Guidance and Telemonitoring Platform on Costs, Readmissions, and Complications After Hip and Knee Arthroplasties. Journal of Arthroplasty. 2018;33(4):988. doi: <http://dx.doi.org/10.1016/j.arth.2017.11.036>.

113. Salehinia R, Akbari H, Formi EN, Haji S, Gharib M, Mehdizadeh H. Effect of a Self-Care Application on Pain and Motor Rehabilitation Following Total Knee Arthroplasty. Journal of Mazandaran University of Medical Sciences. 2023;33(220):91-100. PMID: 2023887254.

114. Seron P, Oliveros M-J, Fuentes-Aspe R, Gutierrez-Arias R, Torres-Castro RC, Merino-Osorio C, et al. Effectiveness of Telerehabilitation in Physical Therapy: A Rapid Overview. Physical therapy. 2021 2021;101(6). PMID: rayyan-230148522.

115. Shah RF, Zaid MB, Bendich I, Hwang KM, Patterson JT, Bini SAA-H, Kevin M., et al. Optimal Sampling Frequency for Wearable Sensor Data in Arthroplasty Outcomes Research. A Prospective Observational Cohort Trial. Journal of Arthroplasty. 2019 2019;34(10):2248-52. PMID: rayyan-230149064.

116. Shim GY, Kim EH, Lee SJ, Chang CB, Lee YS, Lee JI, et al. Postoperative rehabilitation using a digital healthcare system in patients with total knee arthroplasty: a randomized controlled trial. Archives of Orthopaedic and Trauma Surgery. 2023. PMID: 2023039559. doi: <https://dx.doi.org/10.1007/s00402-023-04894-y>.

117. Shin G, Jarrahi MH, Fei Y, Gafinowitz N, Lu X, Karami A, et al. Wearable activity trackers, accuracy, adoption, acceptance and health impact: A systematic literature review. Journal of Biomedical Informatics. 2019 2019;93:103153. PMID: rayyan-230149030.

118. Shukla H, Nair SR, Thakker D. Role of telerehabilitation in patients following total knee arthroplasty: Evidence from a systematic literature review and meta-analysis. Journal of telemedicine and telecare. 2017 2017;23(2):339-46. PMID: rayyan-230149628.

119. Small SR, Bullock GS, Khalid S, Price AJ, Barker K, Trivella MA-S, Scott R, et al. Current clinical utilisation of wearable motion sensors for the assessment of outcome following knee arthroplasty: a scoping review. BMJ open. 2019 2019;9(12):e033832. PMID: rayyan-230148999.

120. Smith WA. Effect of incorporating the fitbit fitness tracking technology into a prescribed exercise intervention program to improve long-term function in obese individuals one year following total knee arthroplasty. Dissertation Abstracts International: Section B: The Sciences and Engineering. 2015;76(2-B(E)):No-Specified.

121. Spangehl MJ. Is It Time for Telerehabilitation to Go Mainstream? The Journal of bone and joint surgery American volume. 2015 2015;97(14):e55. PMID: rayyan-230149954.

122. Sprando D, Dietz MJ, Hanselman AE, Frye BM, Regier MD. Smartphone assessment of knee flexion compared to radiographic standards. Knee. 2017 2017;24(2):224-30. PMID: rayyan-230149437.

123. Stuhlreyer J, Roder C, Krug F, Zollner C, Flor H, Klinger R. A digital application and augmented physician rounds reduce postoperative pain and opioid consumption after primary total knee replacement (TKR): a randomized clinical trial. BMC Medicine. 2022;20(1):469. PMID: 2020443223. doi: <https://dx.doi.org/10.1186/s12916-022-02638-0>.

124. Terada H, Zhu Y, Horiguchi K, Nakamura M, Takahashi R. Development of a wearable assist robot for walk rehabilitation after knee arthroplasty surgery2012 2012. 65-71 p.

125. Tousignant M, Moffet H, Nadeau S, Mérette C, Boissy P, Corriveau H, et al. Cost analysis of in-home telerehabilitation for post-knee arthroplasty. Journal of medical Internet research. 2015 Mar 31;17(3):e83. PMID: 25840501. doi: 10.2196/jmir.3844.

126. Turchetti G, Fusco F. Telerehabilitation after total knee replacement in Italy: Cost-effectiveness and cost-utility analysis of a mixed telerehabilitation-standard rehabilitation programme compared with usual care. BMJ Open. 2016 2016;6(5):e009964. PMID: rayyan-230149647.

127. Velayati F, Ayatollahi H, Hemmat M. A Systematic Review of the Effectiveness of Telerehabilitation Interventions for Therapeutic Purposes in the Elderly. Methods of Information in Medicine. 2020 2020;59(2):104-9. PMID: rayyan-230151628.

128. Veroff DR, Ochoa-Arvelo T, Venator B. A randomized study of telephonic care support in populations at risk for musculoskeletal preference-sensitive surgeries. BMC medical informatics and decision making. 2013 2013;13:21. PMID: rayyan-230150197.

129. Wang Q, Lee RLT, Hunter S, Chan SW-C. The effectiveness of internet-based telerehabilitation among patients after total joint arthroplasty: An integrative review. International journal of nursing studies. 2021 2021;115:103845. PMID: rayyan-230148480.

130. Wang X, Ferreira M, Pozzobon D, Hunter DJ, Vesentini G. Digital disruptive technology for rehabilitation following elective surgery for low back pain, knee and hip osteoarthritis: A systematic review and meta-analysis. Arthritis and Rheumatology. 2018 2018;70:431-2. PMID: rayyan-230149324.

131. Wang X, Pozzobon D, Ferreira ML, Hunter DJ, Vesentini G. Technology-assisted rehabilitation following total knee or hip replacement for people with osteoarthritis: A systematic review and meta-analysis. BMC musculoskeletal disorders. 2019;20(1):506. doi: <http://dx.doi.org/10.1186/s12891-019-2900-x>.

132. Weinberg M, Danoff JR, Scuderi GR. Remote Patient Monitoring Following Total Joint Arthroplasty. Orthopedic Clinics of North America. 2023;54(2):161-8. PMID: 2022474258. doi: <https://dx.doi.org/10.1016/j.ocl.2022.11.002>.

133. Windsor EN, Sharma AK, Gkiatas I, Elbuluk AM, Sculco PK, Vigdorchik JM. An Overview of Telehealth in Total Joint Arthroplasty. HSS Journal. 2021 2021;17(1):51-8. PMID: rayyan-230151577.

134. Wolfstadt JI, Soong C, Ward SE. Improving patient outcomes following total joint arthroplasty: Is there an app for that? BMJ Quality and Safety. 2019 2019;28(10):775-7. PMID: rayyan-230151663.

135. Wong B, Ward D, Gemmell K, Bright R, Blackman R, Sole G, et al. How is telehealth being utilized in the context of rehabilitation for lower limb musculoskeletal disorders: a scoping review. Physical Therapy Reviews. 2020 2020;25(5):350-60. PMID: rayyan-230151647.

136. Wylde V, Bertram W, Sanderson E, Noble S, Howells N, Peters TJ, et al. The STAR care pathway for patients with pain at 3 months after total knee replacement: a multicentre, pragmatic, randomised, controlled trial. The Lancet Rheumatology. 2022;4(3):e188-e97. PMID: 2017000185. doi: <https://dx.doi.org/10.1016/S2665-9913%2821%2900371-4>.

137. Yeowell G, Staniford L, Powell S, Fatoye F, Kelly B. A qualitative study exploring the provider and patient perspective of two rehabilitation programmes following knee replacement surgery. Physiotherapy (United Kingdom). 2020 2020;107:e85-e6. PMID: rayyan-230148937.

138. Yen C-H, Lyu S-R, Hung S-S, Chen W-Y, Chen J-J. Telemedicine Management With Kinetic Sensor for Post-surgical Knee Osteoarthritis Patients. Archives of Physical Medicine and Rehabilitation. 2017 10/01;98:e114. doi: 10.1016/j.apmr.2017.08.367.

139. Zachwieja E, Theosmy EG, Yacovelli SJ, Beatty EW, McGrath ME, Lonner JH. Web-Based Self-Directed Exercise Program Is Cost-Effective Compared to Formal Physical Therapy After Primary Total Knee Arthroplasty. The Journal of arthroplasty. 2020 2020--;35(9):2335-41. PMID: rayyan-230150728.

140. Zhang J, Dushaj K, Scuderi GR, Hepinstall MS, Rasquinha VJ. Monitoring Surgical Incision Sites in Orthopedic Patients Using an Online Physician-Patient Messaging Platform. Journal of Arthroplasty. 2019 2019;34(9):1897-900. PMID: rayyan-230149070.

141. Zheng H, Rosal MC, Oatis CA, Li W, Franklin PD. Tailored system to deliver behavioral intervention and manage data in randomized trials. Journal of medical Internet research. 2013 2013;15(4):e58. PMID: rayyan-230150208.

142. Zhu Y, Nakamura M, Horiuchi T, Kohno H, Takahashi R, Terada H, et al. New wearable walking-type continuous passive motion device for postsurgery walking rehabilitation. Proceedings of the Institution of Mechanical Engineers Part H, Journal of engineering in medicine. 2013 2013;227(7):733-45. PMID: rayyan-230150206.

143. Alexander JS, Redfern RE, Duwelius PJ, Berend KR, Lombardi AV, Crawford DA. Use of a Smartphone-Based Care Platform After Primary Partial and Total Knee Arthroplasty: 1-Year Follow-Up of a Prospective Randomized Controlled Trial. Journal of Arthroplasty. 2023;38(7 Supplement 2):S208-S14. PMID: 2023513257. doi: <https://dx.doi.org/10.1016/j.arth.2023.02.082>.

144. Crawford DA, Duwelius PJ, Sneller MA, Morris MJ, Hurst JM, Berend KR, et al. 2021 Mark Coventry Award: Use of a smartphone-based care platform after primary partial and total knee arthroplasty: a prospective randomized controlled trial. Bone Joint J. 2021 Jun;103-b(6 Supple A):3-12. PMID: 34053272. doi: 10.1302/0301-620x.103b6.Bjj-2020-2352.R1.

145. An J-A, Ryu H-K, Lyu S-J, Yi H-J, Lee B-H. Effects of preoperative telerehabilitation on muscle strength, range of motion, and functional outcomes in candidates for total knee arthroplasty: A single-blind randomized controlled trial. International Journal of Environmental Research and Public Health. 2021 2021;18(11):6071. PMID: rayyan-230148528.

146. Antunes R, Jacob P, Meyer A, Conditt MA, Roche MW, Verstraete MA. Accuracy of measuring knee flexion after tka through wearable imu sensors. Journal of Functional Morphology and Kinesiology. 2021 2021;6(3). PMID: rayyan-230151554.

147. Argent R, Slevin P, Bevilacqua A, Neligan M, Daly A, Caulfield B. Wearable sensor-based exercise biofeedback for orthopaedic rehabilitation: A mixed methods user evaluation of a prototype system. Sensors (Switzerland). 2019;19(2). doi: 10.3390/s19020432.

148. Ayoade M, Morton L, Baillie L. Investigating the feasibility of a wireless motion capture system to aid in the rehabilitation of total knee replacement patients2011 2011. 404-7 p.

149. Backer HC, Schulz MRG, Perka C, Hardt S, Wu CH, Weber-Spickschen TSA-B, Henrik C., et al. App-based rehabilitation program after total knee arthroplasty: a randomized controlled trial. Archives of Orthopaedic and Trauma Surgery. 2021 2021;141(9):1575-82. PMID: rayyan-230148478.

150. Bade MJ, Christensen JC, Zeni JAJ, Christiansen CL, Dayton MR, Forster JE, et al. Movement pattern biofeedback training after total knee arthroplasty: Randomized clinical trial protocol. Contemporary clinical trials. 2020 2020--;91(101242342):105973. PMID: rayyan-230150680.

151. Bettger JP, Hoch BT, de Leon AJ, Seyler TM, Green CL, Holmes DN, et al. Effects of Virtual Exercise Rehabilitation In-Home Therapy Compared with Traditional Care After Total Knee Arthroplasty: VERITAS, a Randomized Controlled Trial. The Journal of bone and joint surgery American volume. 2020 2020;102(2):101-9. PMID: rayyan-230149005.

152. Bini SA, Mahajan J. Clinical outcomes of remote asynchronous telerehabilitation are equivalent to traditional therapy following total knee arthroplasty: A randomized control study. Journal of telemedicine and telecare. 2017 2017;23(2):239-47. PMID: rayyan-230149627.

153. Bitsaki M, Koutras G, Heep H, Koutras C. Cost-effective mobile-based healthcare system for managing total joint arthroplasty follow-up. Healthcare Informatics Research. 2017 2017;23(1):67-73. PMID: rayyan-230151737.

154. Blasco J, Roig-Casasus S, Igual-Camacho C, Diaz-Diaz B, Perez-Maletzki J. Conversational Chatbot to Promote Adherence to Rehabilitation After Total Knee Replacement: Implementation and Feasibility. Archives of Physical Medicine and Rehabilitation. 2022;103(12):e125. PMID: 2021304291. doi: <https://dx.doi.org/10.1016/j.apmr.2022.08.764>.

155. Blasco JM, Diaz-Diaz B, Igual-Camacho C, Perez-Maletzki J, Hernandez-Guilen D, Roig-Casasus S. Effectiveness of using a chatbot to promote adherence to home physiotherapy after total knee replacement, rationale and design of a randomized clinical trial. BMC musculoskeletal disorders. 2023;24(1):491. PMID: 2023866377. doi: <https://dx.doi.org/10.1186/s12891-023-06607-3>.

156. Bolam SM, Batinica B, Yeung TC, Weaver S, Cantamessa A, Vanderboor TC, et al. Remote Patient Monitoring with Wearable Sensors Following Knee Arthroplasty. Sensors (Basel). 2021 2021;21(15). PMID: rayyan-230148451.

157. Bonora S, Amadori E, Fantini C, Merlo A, Prati P, Mazzoli D, et al. Use of a haptic biofeedback in the rehabilitation of patients with total knee arthroplasty (TKA): A pilot study. Gait and Posture. 2017 2017;57:16-7. PMID: rayyan-230149510.

158. Calliess T, Bocklage R, Karkosch R, Marschollek M, Windhagen H, Schulze M. Clinical evaluation of a mobile sensor-based gait analysis method for outcome measurement after knee arthroplasty. Sensors (Basel, Switzerland). 2014 2014--;14(9):15953-64. PMID: rayyan-230151039.

159. Campbell KJ, Louie PK, Bohl DD, Edmiston T, Mikhail C, Li J, et al. A novel, automated text-messaging system is effective in patients undergoing total joint arthroplasty. Journal of Bone and Joint Surgery - American Volume. 2019 2019;101(2):145-51. PMID: rayyan-230149068.

160. Castle H, Kozak K, Goonatillake H, Sidhu A, Haebich S, Bowden V, et al. Smartphone technology: a reliable and valid measure of knee movement in knee replacement. International journal of rehabilitation research Internationale Zeitschrift fur Rehabilitationsforschung Revue internationale de recherches de readaptation. 2018 2018;41(2):152-8. PMID: rayyan-230149394.

161. Chapman RM, Moschetti WE, Van Citters DW. Is clinically measured knee range of motion after total knee arthroplasty 'good enough?': A feasibility study using wearable inertial measurement units to compare knee range of motion captured during physical therapy versus at home. Medicine in Novel Technology and Devices. 2021;11:100085. PMID: 2013564337. doi: <https://dx.doi.org/10.1016/j.medntd.2021.100085>.

162. Chiang C-Y, Chen K-H, Liu K-C, Hsu SJ-P, Chan C-T. Data Collection and Analysis Using Wearable Sensors for Monitoring Knee Range of Motion after Total Knee Arthroplasty. Sensors (Basel, Switzerland). 2017 2017;17(2). PMID: rayyan-230149599.

163. Christensen JC, Stanley EC, Oro EG, Carlson HB, Naveh YY, Shalita R, et al. The validity and reliability of the OneStep smartphone application under various gait conditions in healthy adults with feasibility in clinical practice. Journal of orthopaedic surgery and research. 2022;17(1):417. PMID: 639035322. doi: <https://dx.doi.org/10.1186/s13018-022-03300-4>.

164. Chughtai M, Sultan AA, Khlopas A, Mont MA, Newman JM, Navarro SM, et al. The Role of Virtual Rehabilitation in Total Knee and Hip Arthroplasty. Surgical technology international. 2018 2018;32:299-305. PMID: rayyan-230149391.

165. Chughtai M, Sultan AA, Khlopas A, Sodhi N, Kolczun MC, Mont MA, et al. The Role of Virtual Rehabilitation in Total and Unicompartmental Knee Arthroplasty. Journal of Knee Surgery. 2019 2019;32(1):105-10. PMID: rayyan-230149103.

166. Chughtai M, Sultan AA, Samuel LT, Shah NV, Newman JM, Solow M, et al. The role of prehabilitation with a telerehabilitation system prior to total knee arthroplasty. Annals of Translational Medicine. 2019 2019;7(4):68. PMID: rayyan-230149207.

167. Colomina J, Drudis R, Torra M, Pallisó F, Massip M, Vargiu E, et al. Implementing mhealth-enabled integrated care for complex chronic patients with osteoarthritis undergoing primary hip or knee arthroplasty: Prospective, two-arm, parallel trial. Journal of medical Internet research. 2021 2021;23(9). PMID: rayyan-230151551.

168. Correia FD, Nogueira A, Magalhães I, Guimarães J, Moreira M, Barradas I, et al. Medium-term outcomes of digital versus conventional home-based rehabilitation after total knee arthroplasty: Prospective, parallel-group feasibility study. JMIR Rehabilitation and Assistive Technologies. 2019 2019;6(1). PMID: rayyan-230151684.

169. Correia FD, Nogueira A, Magalhães I, Guimarães J, Moreira M, Barradas I, et al. Home-based Rehabilitation With A Novel Digital Biofeedback System versus Conventional In-person Rehabilitation after Total Knee Replacement: a feasibility study. Scientific Reports. 2018;8(1). doi: 10.1038/s41598-018-29668-0.

170. De Berardinis L, Senarighi M, Ciccullo C, Forte F, Spezia M, Gigante AP. Fast-track surgery and telerehabilitation protocol in unicompartmental knee arthroplasty leads to superior outcomes when compared with the standard protocol: a propensity-matched pilot study. Knee surgery & related research. 2022;34(1):44. doi: <https://dx.doi.org/10.1186/s43019-022-00173-z>.

171. De Vroey H, Staes F, Weygers I, Vereecke E, Vanrenterghem J, Deklerck J, et al. The implementation of inertial sensors for the assessment of temporal parameters of gait in the knee arthroplasty population. Clinical Biomechanics. 2018 2018;54:22-7. PMID: rayyan-230151704.

172. Doiron-Cadrin P, Lowry V, Desmeules F, Kairy D, Vendittoli P-A, Poitras SA-D-C, Patrick, et al. Feasibility and preliminary effects of a tele-prehabilitation program and an in-person prehablitation program compared to usual care for total hip or knee arthroplasty candidates: a pilot randomized controlled trial. Disability and rehabilitation. 2020 2020;42(7):989-98. PMID: rayyan-230148544.

173. Doiron-Cadrin P, Kairy D, Vendittoli PA, Lowry V, Poitras S, Desmeules F. Effects of a tele-prehabilitation program or an in-person prehabilitation program in surgical candidates awaiting total hip or knee arthroplasty: Protocol of a pilot single blind randomized controlled trial. Contemporary Clinical Trials Communications. 2016;4:192-8. doi: 10.1016/j.conctc.2016.10.001.

174. Duong V, Dennis S, Ferreira ML, Heller G, Nicolson PJA, Robbins SR, et al. Predictors of Adherence to a Step Count Intervention Following Total Knee Replacement: An Exploratory Cohort Study. The Journal of orthopaedic and sports physical therapy. 2022;52(9):620-9. PMID: 638437192. doi: <https://dx.doi.org/10.2519/jospt.2022.11133>.

175. Duong V, Dennis S, Harris A, Robbins SR, Venkatesha V, Ferreira M, et al. The Effects Of A Disruptive Digital Technology Intervention Following Total Knee Replacement: Results From The Pathway Randomised Controlled Trial. Osteoarthritis and Cartilage. 2023;31(Supplement 1):S30-S1. PMID: 2023242812. doi: <https://dx.doi.org/10.1016/j.joca.2023.01.521>.

176. Wang X, Robbins S, Capistrano S, Duong V, Hunter DJ, Melo L, et al. Participatory health through behavioural engagement and disruptive digital technology for postoperative rehabilitation: Protocol of the PATHway trial. BMJ open. 2021 2021;11(1):e041328. PMID: rayyan-230148605.

177. Eichler S, Salzwedel A, Rabe S, Mueller S, Mayer F, Wochatz M, et al. The effectiveness of telerehabilitation as a supplement to rehabilitation in patients after total knee or hip replacement: Randomized controlled trial. JMIR Rehabilitation and Assistive Technologies. 2019 2019;6(2). PMID: rayyan-230151671.

178. Eichler S, Rabe S, Salzwedel A, Voller H, Muller S, Stoll J, et al. Effectiveness of an interactive telerehabilitation system with home-based exercise training in patients after total hip or knee replacement: Study protocol for a multicenter, superiority, no-blinded randomized controlled trial. Trials. 2017 2017;18(1):438. PMID: rayyan-230149396.

179. Hussain MS, Li J, Brindal E, van Kasteren Y, Varnfield M, Reeson A, et al. Supporting the delivery of total knee replacements care for both patients and their clinicians with a mobile app and web-based tool: Randomized controlled trial protocol. JMIR Research Protocols. 2017 2017;6(3). PMID: rayyan-230151731.

180. Farr-Wharton G, Li J, Hussain MS, Freyne J, editors. Mobile supported health services: Experiences in orthopaedic care. Proceedings - IEEE Symposium on Computer-Based Medical Systems; 2020.

181. Ficklscherer A, Stapf J, Meissner KM, Niethammer T, Lahner M, Wagenhauser M, et al. Testing the feasibility and safety of the Nintendo Wii gaming console in orthopedic rehabilitation: a pilot randomized controlled study. Arch Med Sci. 2016 Dec 1;12(6):1273-8. PMID: 27904518. doi: 10.5114/aoms.2016.59722.

182. Fisher C, Biehl E, Titmuss MP, Schwartz R, Gantha CS. HSS@Home, Physical Therapist-Led Telehealth Care Navigation for Arthroplasty Patients: A Retrospective Case Series. HSS Journal. 2019 2019;15(3):226-33. PMID: rayyan-230151656.

183. LeBrun DG, Malfer C, Wilson M, Carroll KM, Wang Ms V, Mayman DJ, et al. Telemedicine in an Outpatient Arthroplasty Setting During the COVID-19 Pandemic: Early Lessons from New York City. HSS journal : the musculoskeletal journal of Hospital for Special Surgery. 2021 2021;17(1):25-30. PMID: rayyan-536818987. doi: doi:<https://dx.doi.org/10.1177/1556331620972659> PT - Journal Article.

184. Fung V, Ho A, Shaffer J, Chung E, Gomez M. Use of Nintendo Wii FitTM In the rehabilitation of outpatients following total knee replacement: A preliminary randomised controlled trial. Physiotherapy (United Kingdom). 2012 2012;98(3):183-8. PMID: rayyan-230150226.

185. Gianola S, Castellini G, Stucovitz E, Mascali M, Vanni F, Tornese D, et al. Effects of early virtual reality-based rehabilitation in patients with total knee arthroplasty: A randomized controlled trial. Medicine (United States). 2020 2020;99(7):e19136. PMID: rayyan-230148885.

186. Gohir SA, Eek F, Kelly A, Abhishek A, Valdes AM. Effectiveness of Internet-Based Exercises Aimed at Treating Knee Osteoarthritis: The iBEAT-OA Randomized Clinical Trial. JAMA Netw Open. 2021 Feb 1;4(2):e210012. PMID: 33620447. doi: 10.1001/jamanetworkopen.2021.0012.

187. Gray J, McCarthy S, Carr E, Danjoux G, Hackett R, McCarthy A, et al. The impact of a digital joint school educational programme on post-operative outcomes following lower limb arthroplasty: a retrospective comparative cohort study. BMC health services research. 2022;22(1):580. PMID: 637906204. doi: <https://dx.doi.org/10.1186/s12913-022-07989-1>.

188. Gunduz CS, Caliskan N. The Effect of Preoperative Video Based Pain Training on Postoperative Pain and Analgesic Use in Patients Undergoing Total Knee Arthroplasty: A Non-randomized Control Group Intervention Study. Clinical nursing research. 2021;30(6):741-52. PMID: 633906421. doi: <https://dx.doi.org/10.1177/1054773820983361>.

189. Haase I, Kladny B, Eisermann U. Computer-aided multimedia training in orthopedic rehabilitation. American Journal of Physical Medicine and Rehabilitation. 2004 2004;83(9):670-80. PMID: rayyan-230150610.

190. Hadamus A, Bialoszewski D, Urbaniak E, Kowalska AJ, Wydra K, Boratynski R, et al. The impact of training in virtual reality on balance in patients after total knee replacement is relatively slight. Gait and Posture. 2020 2020;81:134-5. PMID: rayyan-230148930.

191. Hardwick-Morris M, Carlton S, Twiggs J, Miles B, Liu D. Pre- and postoperative physiotherapy using a digital application decreases length of stay without reducing patient outcomes following total knee arthroplasty. Arthroplasty. 2022;4(1):30. PMID: 2018392712. doi: <https://dx.doi.org/10.1186/s42836-022-00133-8>.

192. Harmelink KEM, Nijhuis-Van Der Sanden MWG, Zeegers AVCM, Tonis TM, Hullegie W, Staal JB. The effectiveness of the use of a digital activity coaching system in addition to a two-week home-based exercise program in patients after total knee arthroplasty: Study protocol for a randomized controlled trial. BMC musculoskeletal disorders. 2017 2017;18(1):290. PMID: rayyan-230149418.

193. Hong M, Loeb J, Yang M, Bailey JF. Postoperative Outcomes of a Digital Rehabilitation Program After Total Knee Arthroplasty: Retrospective, Observational Feasibility Study. JMIR formative research. 2022;6(9):e40703. doi: <https://dx.doi.org/10.2196/40703>.

194. Huang P, He J, Zhang YM. The mobile application of patient management in education and follow-up for patients following total knee arthroplasty. Zhonghua yi xue za zhi. 2017 2017;97(20):1592-5. PMID: rayyan-230151730.

195. Huang Y-P, Liu Y-Y, Hsu W-H, Lai L-J, Lee MS. Monitoring and assessment of rehabilitation progress on range of motion after total knee replacement by sensor-based system. Sensors (Switzerland). 2020 2020;20(6). PMID: rayyan-230151635.

196. Hung L-P, Chao Y-H, Tseng Y-L, Chung Y-L. Constructing a home-based knee replacement exercise monitoring system with G sensor2018 2018. 627-36 p.

197. Janhunen M, Katajapuu N, Paloneva J, Pamilo K, Oksanen A, Keemu H, et al. Effects of a home-based, exergaming intervention on physical function and pain after total knee replacement in older adults: a randomised controlled trial. BMJ Open Sport and Exercise Medicine. 2023;9(1):e001416. PMID: 2023211031. doi: <https://dx.doi.org/10.1136/bmjsem-2022-001416>.

198. Jansson M, Vuorinen A-L, Harjumaa M, Simila H, Koivisto J, Puhto A-P, et al. The digital patient journey solution for patients undergoing elective hip and knee arthroplasty: Protocol for a pragmatic randomized controlled trial. Journal of advanced nursing. 2020 2020;76(6):1436-48. PMID: rayyan-230148658.

199. Jenny J-Y. Measurement of the knee flexion angle with a smartphone-application is precise and accurate. Journal of Arthroplasty. 2013 2013;28(5):784-7. PMID: rayyan-230150142.

200. Jenny J-Y, Bureggah A, Diesinger Y. Measurement of the knee flexion angle with smartphone applications: Which technology is better? Knee surgery, sports traumatology, arthroscopy : official journal of the ESSKA. 2016 2016;24(9):2874-7. PMID: rayyan-230149815.

201. Juhl CB, Roth S, Schierbeck R, Nielsen LN, Nordlien A-D, Hansen NF, et al. Effectiveness of technology assisted exercise compared to usual care in total knee arthroplasty. Osteoarthritis and Cartilage. 2016 2016;24:S473. PMID: rayyan-230149778.

202. Klement MR, Rondon AJ, McEntee RM, Greenky MR, Austin MS. Web-Based, Self-Directed Physical Therapy After Total Knee Arthroplasty Is Safe and Effective for Most, but Not All, Patients. The Journal of arthroplasty. 2019 2019--;34(7):S178-S82. PMID: rayyan-230150820.

203. Kline PW, Melanson EL, Sullivan WJ, Miller MJ, Stevens-Lapsley JE, Christiansen CL, et al. Improving Physical Activity Through Adjunct Telerehabilitation Following Total Knee Arthroplasty: Randomized Controlled Trial Protocol. Physical therapy. 2019 2019;99(1):37-45. PMID: rayyan-230148914.

204. Knapp PW, Keller RA, Mabee KA, Frisch NB, Pillai R. Quantifying Patient Engagement in Total Joint Arthroplasty Using Digital Application-Based Technology. Journal of Arthroplasty. 2021;36(9):3108-17. doi: <http://dx.doi.org/10.1016/j.arth.2021.04.022>.

205. Kontadakis G, Chasiouras D, Proimaki D, Halkiadakis M, Fyntikaki M, Mania K. Gamified platform for rehabilitation after total knee replacement surgery employing low cost and portable inertial measurement sensor node. Multimedia Tools and Applications. 2020;79(5-6):3161-88. doi: 10.1007/s11042-018-6572-6.

206. Kramer JF, Speechley M, Bourne R, Rorabeck C, Vaz M. Comparison of clinic- and home-based rehabilitation programs after total knee arthroplasty. Clinical orthopaedics and related research. 2003 May(410):225-34. PMID: 12771834. doi: 10.1097/01.blo.0000063600.67412.11.

207. Krebs DE, Huddleston JI, Goldvasser D, Scarborough DM, Harris WH, Malchau H. Biomotion community-wearable human activity monitor: Total knee replacement and healthy control subjects2006 2006. 109-12 p.

208. Kuether J, Moore A, Kahan J, Martucci J, Messina T, Perreault R, et al. Telerehabilitation for Total Hip and Knee Arthroplasty Patients: A Pilot Series with High Patient Satisfaction. HSS Journal. 2019 2019;15(3):221-5. PMID: rayyan-230151657.

209. Lam AWK, Varona-Marin D, Li Y, Fergenbaum M, Kulic D, Ananthanarayan A, Ayoade, Burns, Burns, Costa, Friedrich, Gockley, Houmanfar, Huang, Kimel, Lam, Lange, Li, Lin, Lin, Lin, Lindeman, Norkin, Piqueras, Rosati, Schmitz, Shin, Singh, Stewart, Sucar, Uzor, Yeh. Automated rehabilitation system: Movement measurement and feedback for patients and physiotherapists in the rehabilitation clinic. Human-Computer Interaction. 2016 2016;31(3):294-334. PMID: rayyan-230151475.

210. Lebleu J, Pauwels A, Anract P, Parratte S, Van Overschelde P, Van Onsem S. Digital Rehabilitation after Knee Arthroplasty: A Multi-Center Prospective Longitudinal Cohort Study. Journal of Personalized Medicine. 2023;13(5):824. PMID: 2023412824. doi: <https://dx.doi.org/10.3390/jpm13050824>.

211. Levinger P, Zeina D, Teshome AK, Begg R, Skinner E, Abbott JH. A real time biofeedback using Kinect and Wii to improve gait for post-total knee replacement rehabilitation: a case study report. Disability and rehabilitation Assistive technology. 2016 2016;11(3):251-62. PMID: rayyan-230149816.

212. Li Y, Gu Z, Ning R, Yin H. Study on the effect of internet plus continuous nursing on functional recovery and medication compliance of patients with knee joint replacement. Journal of orthopaedic surgery and research. 2023;18(1):424. PMID: 641548999. doi: <https://dx.doi.org/10.1186/s13018-023-03907-1>.

213. Liptak MG, Krishnan J, Theodoulou A, Kaambwa B, Saunders S, Hinrichs SW, et al. The safety, efficacy and cost-effectiveness of the Maxm Skate, a lower limb rehabilitation device for use following total knee arthroplasty: Study protocol for a randomised controlled trial. Trials. 2019 2019;20(1):36. PMID: rayyan-230149091.

214. Losina E, Ghazinouri R, Wright J, Daigle ME, Donnell-Fink LA, Prokopetz JJZ, et al. The AViKA (Adding Value in Knee Arthroplasty) postoperative care navigation trial: Rationale and design features. BMC musculoskeletal disorders. 2013;14:290. doi: <http://dx.doi.org/10.1186/1471-2474-14-290>.

215. Lou N, Diao Y, Chen Q, Ning Y, Liang S, Li G, et al. A Portable Wearable Inertial System for Rehabilitation Monitoring and Evaluation of Patients With Total Knee Replacement. Frontiers in Neurorobotics. 2022;16:836184. PMID: 2015537719. doi: <https://dx.doi.org/10.3389/fnbot.2022.836184>.

216. Lu G, Tan Q, Wu Z, Shi L, Zhong Y, Wu TA-W, Tingting, et al. The effect of a micro-visual intervention on the accelerated recovery of patients with kinesiophobia after total knee replacement during neo-coronary pneumonia. Medicine. 2021 2021;100(6):e24141. PMID: rayyan-230148711.

217. Mark-Christensen T, Thorborg K, Kallemose T, Bandholm T. Physical rehabilitation versus no physical rehabilitation after total hip and knee arthroplasties: Protocol for a pragmatic, randomized, controlled, superiority trial (The DRAW1 trial). F1000Research. 2021;10:146. doi: <http://dx.doi.org/10.12688/f1000research.50814.1>.

218. Marques CJ, Bauer C, Grimaldo D, Tabeling S, Weber T, Ehlert A, et al. Sensor Positioning Influences the Accuracy of Knee Rom Data of an E-Rehabilitation System: A Preliminary Study with Healthy Subjects. Sensors (Basel). 2020 2020;20(8). PMID: rayyan-230148730.

219. McDonall J, Redley B, De Steiger R, Reynolds J, Livingston P, Botti M. Patient participation in postoperative care activities in patients undergoing total knee replacement surgery: Multimedia Intervention for Managing patient Experience (MIME). Study protocol for a cluster randomised crossover trial. BMC Musculoskeletal Disorders. 2016 2016;17(1):294. PMID: rayyan-230149667.

220. McDonall J, de Steiger R, Reynolds J, Redley B, Livingston PM, Hutchinson AF, et al. Patient activation intervention to facilitate participation in recovery after total knee replacement (MIME): a cluster randomised cross-over trial. BMJ Qual Saf. 2019 Oct;28(10):782-92. PMID: 30975730. doi: 10.1136/bmjqs-2018-008975.

221. McDonall J, Redley B, Livingston P, Hutchinson A, de Steiger R, Botti M. A Nurse-Led Multimedia Intervention to Increase Patient Participation in Recovery After Knee Arthroplasty: Hybrid Type II Implementation Study. JMIR human factors. 2022 2022;9(2):e36959. PMID: rayyan-536818967. doi: doi:<https://dx.doi.org/10.2196/36959> PT - Journal Article.

222. Mehta SJ, Norton L, Freeman J, Marcus N, Reitz C, McDonald C, et al. Effect of Remote Monitoring on Discharge to Home, Return to Activity, and Rehospitalization after Hip and Knee Arthroplasty: A Randomized Clinical Trial. JAMA Network Open. 2020 2020:e2028328. PMID: rayyan-230148667.

223. Mehta SP, Barker K, Bowman B, Galloway H, Oliashirazi N, Oliashirazi A. Reliability, Concurrent Validity, and Minimal Detectable Change for iPhone Goniometer App in Assessing Knee Range of Motion. Journal of Knee Surgery. 2017;30(6):577-84. doi: 10.1055/s-0036-1593877.

224. Mehta SP, Bremer H, Cyrus H, Milligan A, Oliashirazi A. Smartphone goniometer has excellent reliability between novice and experienced physical therapists in assessing knee range of motion. Journal of Bodywork and Movement Therapies. 2021;25:67-74. doi: 10.1016/j.jbmt.2020.11.021.

225. Milliren CE, Lindsay B, Biernat L, Smith TA, Weaver B. Can digital engagement improve outcomes for total joint replacements? Digital health. 2022;8:20552076221095322. doi: <https://dx.doi.org/10.1177/20552076221095322>.

226. Msayib Y, Gaydecki P, Ismail S, Callaghan M, Dale N. An Intelligent Remote Monitoring System for Total Knee Arthroplasty Patients. Journal of Medical Systems. 2017;41(6):90. doi: <http://dx.doi.org/10.1007/s10916-017-0735-2>.

227. Na A, Buchanan TS. Validating Wearable Sensors Using Self-Reported Instability among Patients with Knee Osteoarthritis. PM and R. 2021;13(2):119-27. PMID: 2005834812. doi: <https://dx.doi.org/10.1002/pmrj.12393>.

228. Negus JJ, Cawthorne DP, Chen JS, Scholes CJ, Parker DA, March LM. Patient outcomes using Wii-enhanced rehabilitation after total knee replacement - the TKR-POWER study. Contemporary clinical trials. 2015 2015--;40(101242342):47-53. PMID: rayyan-230151016.

229. Nuevo M, Mahdavi H, Rodriguez D, Faura T, Fabrellas N, Balocco S, et al. Evaluation of Safety and Efficacy of ReHub in Patients Who Underwent Primary Total Knee Arthroplasty: Study Protocol for a Randomized Controlled Trial. International journal of surgery protocols. 2021;25(1):34-41. doi: <https://dx.doi.org/10.29337/ijsp.138>.

230. Nuevo M, Rodriguez-Rodriguez D, Jauregui R, Fabrellas N, Zabalegui A, Conti M, et al. Telerehabilitation following fast-track total knee arthroplasty is effective and safe: a randomized controlled trial with the ReHub platform. Disability and rehabilitation. 2023:1-11. PMID: 641748483. doi: <https://dx.doi.org/10.1080/09638288.2023.2228689>.

231. Onyeukwu C, Smith CN, Oh A, McClincy MP, Bell KM, Devito Dabbs A, et al. A Portable System for Remote Rehabilitation Following a Total Knee Replacement: A Pilot Randomized Controlled Clinical Study. Sensors (Basel). 2020 2020;20(21). PMID: rayyan-230148668.

232. Osterloh J, Knaack F, Bader R, Behrens M, Peschers J, Nawrath L, et al. The effect of a digital-assisted group rehabilitation on clinical and functional outcomes after total hip and knee arthroplasty-a prospective randomized controlled pilot study. BMC musculoskeletal disorders. 2023;24(1):190. PMID: 2022083411. doi: <https://dx.doi.org/10.1186/s12891-023-06270-8>.

233. Park KH, Song MR. The Effects of Postdischarge Telephone Counseling and Short Message Service on the Knee Function, Activities of Daily Living, and Life Satisfaction of Patients Undergoing Total Knee Replacement. Orthopedic nursing. 2017 2017;36(3):229-36. PMID: rayyan-230149629.

234. Park SA, Jeong Y. The Effect of a Multidimensional Home Rehabilitation Program for Post-Total Knee Arthroplasty Elderly Patients. Orthopedic nursing. 2023;42(1):22-32. PMID: 640138666. doi: <https://dx.doi.org/10.1097/NOR.0000000000000913>.

235. Pellegrini CA, DeVivo KE, Harpine CE, Lee J, Del Gaizo DJ, Wilcox SA-P, Christine A., et al. Reducing sedentary time using an innovative mHealth intervention among patients with total knee replacement: Rationale and study protocol. Contemporary Clinical Trials Communications. 2021 2021;22:100810. PMID: rayyan-230148421.

236. Pereira LC, Rwakabayiza S, Lecureux E, Jolles BM. Reliability of the Knee Smartphone-Application Goniometer in the Acute Orthopedic Setting. Journal of Knee Surgery. 2017 2017;30(3):223-30. PMID: rayyan-230149617.

237. Pfeufer D, Monteiro P, Gililland J, Anderson MB, Stagg M, Pelt C, et al. Immediate Postoperative Improvement in Gait Parameters following Primary Total Knee Arthroplasty Can Be Measured with an Insole Sensor Device. Journal of Knee Surgery. 2020 2020. PMID: rayyan-230148963.

238. Piqueras M, Marco E, Coll M, Escalada F, Ballester A, Cinca C, et al. Effectiveness of an interactive virtual telerehabilitation system in patients after total knee arthoplasty: a randomized controlled trial. Journal of rehabilitation medicine : official journal of the UEMS European Board of Physical and Rehabilitation Medicine. 2013 2013;45(4):392-6. PMID: rayyan-230150202.

239. Pournajaf S, Goffredo M, Criscuolo S, Damiani C, Galli M, Franceschini M. Virtual reality rehabilitation in patients with total knee replacement: Preliminary results. Gait and Posture. 2017 2017;57:17-8. PMID: rayyan-230149509.

240. Pronk Y, Maria Peters MCW, Sheombar A, Brinkman JM. Effectiveness of a mobile eHealth app in guiding patients in pain control and opiate use after total knee replacement: Randomized controlled trial. JMIR mHealth and uHealth. 2020;8(3). doi: 10.2196/16415.

241. Ramkumar PN, Patterson BM, Haeberle HS, Ramanathan D, Cantrell WA, Bloomfield M, et al. Remote Patient Monitoring Using Mobile Health for Total Knee Arthroplasty: Validation of a Wearable and Machine Learning-Based Surveillance Platform. Journal of Arthroplasty. 2019 2019;34(10):2253-9. PMID: rayyan-230149058.

242. Russell TG, Jull GA, Buttrum P, Wootton R. Internet-based outpatient telerehabilitation for patients following total knee arthroplasty: A randomized controlled trial. Journal of Bone and Joint Surgery - Series A. 2011 2011;93(2):113-20. PMID: rayyan-230150400.

243. Russell TG, Buttrum P, Wootton R, Jull GA. Low-bandwidth telerehabilitation for patients who have undergone total knee replacement: preliminary results. Journal of telemedicine and telecare. 2003 2003;9:S44-7. PMID: rayyan-230150601.

244. Scheper H, Derogee R, Visser LG, de Boer MGJ, Mahdad R, van der Wal RJP, et al. A mobile app for postoperative wound care after arthroplasty: Ease of use and perceived usefulness. International Journal of Medical Informatics. 2019;129:75-80. doi: <http://dx.doi.org/10.1016/j.ijmedinf.2019.05.010>.

245. Smulders K, Boekesteijn RJ, Geurts ACH, Smolders JMH, Busch VJJF. Independent and sensitive gait parameters for objective evaluation in knee and hip osteoarthritis using wearable sensors. BMC Musculoskeletal Disorders. 2021 2021;22(1):242. PMID: rayyan-230148486.

246. Stauber A, Schusler N, Schurholz N, Bruns D, Osterbrink J, Nestler N, et al. RECOVER-E - A mobile app for patients undergoing total knee or hip replacement: Study protocol. BMC musculoskeletal disorders. 2020 2020;21(1):71. PMID: rayyan-230148890.

247. Straat AC, Maarleveld JM, Smit DJM, Visch L, Hulsegge G, Huirne JAF, et al. (Cost-)effectiveness of a personalized multidisciplinary eHealth intervention for knee arthroplasty patients to enhance return to activities of daily life, work and sports - rationale and protocol of the multicentre ACTIVE randomized controlled trial. BMC musculoskeletal disorders. 2023;24(1):162. PMID: 2021933069. doi: <https://dx.doi.org/10.1186/s12891-023-06236-w>.

248. Strahl A, Graichen H, Haas H, Hube R, Perka C, Rolvien T, et al. Evaluation of the patient-accompanying app "alley ortho companion" for patients with osteoarthritis of the knee and hip: study protocol for a randomized controlled multi-center trial. Trials. 2022;23(1):716. PMID: 638881991. doi: <https://dx.doi.org/10.1186/s13063-022-06662-6>.

249. Su C-H, Cheng C-H. Developing and evaluating creativity gamification rehabilitation system: The application of PCA-ANFIS based emotions model. Special Issue: Exploring Computing's Impact on Design and Creativity in MST Education. 2016;12(5):1443-68.

250. Summers SH, Nunley RM, Slotkin EM. A Home-Based, Remote-Clinician-Controlled, Physical Therapy Device Leads to Superior Outcomes When Compared to Standard Physical Therapy for Rehabilitation After Total Knee Arthroplasty. Journal of Arthroplasty. 2023;38(3):497-501. PMID: 2021049224. doi: <https://dx.doi.org/10.1016/j.arth.2022.10.009>.

251. Szots K, Konradsen H, Solgaard S, Ostergaard B. Telephone Follow-Up by Nurse After Total Knee Arthroplasty: Results of a Randomized Clinical Trial. Orthopedic nursing. 2016 2016;35(6):411-20. PMID: rayyan-230149801.

252. Timmers T, Kool RB, Janssen L, van der Weegen W, Das D, Marijnissen W-J, et al. The Effect of an App for Day-to-Day Postoperative Care Education on Patients With Total Knee Replacement: Randomized Controlled Trial. JMIR mHealth and uHealth. 2019 2019;7(10):e15323. PMID: rayyan-230149004.

253. Torpil B, Kaya Ö. The Effectiveness of Client-Centered Intervention With Telerehabilitation Method After Total Knee Arthroplasty. OTJR (Thorofare N J). 2022 Jan;42(1):40-9. PMID: 34423693. doi: 10.1177/15394492211038293.

254. Tousignant M, Boissy P, Cabana F, Moffet H, Corriveau H, Marquis F. A randomized controlled trial of home telerehabilitation for post-knee arthroplasty. Journal of Telemedicine and Telecare. 2011 2011;17(4):195-8. PMID: rayyan-230150323.

255. Cabana F, Boissy P, Tousignant M, Moffet H, Corriveau H, Dumais R. Interrater agreement between telerehabilitation and face-to-face clinical outcome measurements for total knee arthroplasty. Telemedicine journal and e-health : the official journal of the American Telemedicine Association. 2010 2010;16(3):293-8. PMID: rayyan-230150420.

256. Moffet H, Boissy P, Corriveau H, Cabana F, Tousignant M, Marquis E. Patients and physiotherapists satisfaction of in-home telerehabilittion for post-knee arthroplasty. Physiotherapy (United Kingdom). 2011 2011;97:eS1246-eS7. PMID: rayyan-230150332.

257. Moffet H, Tousignant M, Nadeau S, Merette C, Boissy P, Corriveau H, et al. In-Home Telerehabilitation Compared with Face-to-Face Rehabilitation After Total Knee Arthroplasty: A Noninferiority Randomized Controlled Trial. The Journal of bone and joint surgery American volume. 2015 2015;97(14):1129-41. PMID: rayyan-230149953.

258. Tousignant M, Boissy P, Corriveau H, Moffet H, Nadeau S, Merette C, et al. Is tele-rehabilitation an adequate economic alternative to conventional rehabilitation? Physiotherapy (United Kingdom). 2015 2015;101:eS1528. PMID: rayyan-230149892.

259. Tousignant M, Hamel M, Brière S. In-home telerehabilitation as an alternative to face-to-face treatment: Feasability in post-knee arthroplasty, speech therapy and chronic obstructive pulmonary disease2010 2010. 30-4 p.

260. Tripuraneni KR, Foran JRH, Munson NR, Racca NE, Carothers JT. A Smartwatch Paired With A Mobile Application Provides Postoperative Self-Directed Rehabilitation Without Compromising Total Knee Arthroplasty Outcomes: A Randomized Controlled Trial. Journal of Arthroplasty. 2021;36(12):3888-93. PMID: 2014321305. doi: <https://dx.doi.org/10.1016/j.arth.2021.08.007>.

261. van Dijk-Huisman HC, Weemaes ATR, Lenssen AF, Boymans TAEJ, de Bie RA. Smartphone App with an Accelerometer Enhances Patients' Physical Activity Following Elective Orthopedic Surgery: A Pilot Study. Sensors (Basel). 2020;20(15). doi: <http://dx.doi.org/10.3390/s20154317>.

262. Visperas AT, Greene KA, Krebs VE, Klika AK, Piuzzi NS, Higuera-Rueda CA. A Web-Based Interactive Patient-Provider Software Platform Does Not Increase Patient Satisfaction or Decrease Hospital Resource Utilization in Total Knee and Hip Arthroplasty Patients in a Single Large Hospital System. Journal of Arthroplasty. 2021 2021;36(7):2290. PMID: rayyan-230148546.

263. Wang Q, Hunter S, Lee RLT, Chan SWC. The effectiveness of a mobile application-based programme for rehabilitation after total hip or knee arthroplasty: A randomised controlled trial. International journal of nursing studies. 2023;140:104455. PMID: 640400607. doi: <https://dx.doi.org/10.1016/j.ijnurstu.2023.104455>.

264. Wang Q, Hunter S, Lee RLT, Wang X, Chan SWC. Mobile rehabilitation support versus usual care in patients after total hip or knee arthroplasty: study protocol for a randomised controlled trial. Trials. 2022;23(1):553. PMID: 2018194806. doi: <https://dx.doi.org/10.1186/s13063-022-06269-x>.

265. Yang C, Shang L, Yao S, Ma J, Xu C. Cost, time savings and effectiveness of wearable devices for remote monitoring of patient rehabilitation after total knee arthroplasty: study protocol for a randomized controlled trial. Journal of orthopaedic surgery and research. 2023;18(1):461. PMID: 641702482. doi: <https://dx.doi.org/10.1186/s13018-023-03898-z>.

266. Youn IH, Youn JH, Zeni JA, Knarr BA. Biomechanical gait variable estimation using wearable sensors after unilateral total knee arthroplasty. Sensors (Switzerland). 2018;18(5). doi: 10.3390/s18051577.

267. Zhang H, Zhou Y. Concept verification of a Remote Automatic Scoring System for Evaluating Knee Function after Total Knee Arthroplasty. Journal of Knee Surgery. 2021 2021;34(4):464-70. PMID: rayyan-230148681.

268. Zhang X, Li G, Zhu C, Chen X, Kourkoumelis N, Gao RA-Z, Xianzuo, et al. A Social Media-Promoted Educational Community of Joint Replacement Patients Using the WeChat App: Survey Study. JMIR mHealth and uHealth. 2021 2021;9(3):e18763. PMID: rayyan-230148630.

269. Zhao W, Yang S, Luo X. Towards rehabilitation at home after total knee replacement. Tsinghua Science and Technology. 2021 2021;26(6):791-9. PMID: rayyan-230151548.

270. Zheng Q, Chen H. A monitoring system for walking rehabilitation after THR or TKR surgeries. Conference proceedings : Annual International Conference of the IEEE Engineering in Medicine and Biology Society IEEE Engineering in Medicine and Biology Society Annual Conference. 2017 2017;2017:2373-6. PMID: rayyan-230149594.

271. Milliren CE, Lindsay B, Biernat L, Smith TA, Weaver B. Can digital engagement improve outcomes for total joint replacements? Digital health. 2022 2022;8(101690863):20552076221095322. PMID: rayyan-536818970. doi: doi:<https://dx.doi.org/10.1177/20552076221095322> PT - Journal Article.

272. Boissy P, Tousignant M, Moffet H, Nadeau S, Brière S, Mérette C, et al. Conditions of Use, Reliability, and Quality of Audio/Video-Mediated Communications during In-Home Rehabilitation Teletreatment for Postknee Arthroplasty. Telemedicine and e-Health. 2016;22(8):637-49. doi: 10.1089/tmj.2015.0157.

273. Jansson MM, Harjumaa M, Puhto A-P, Pikkarainen M. Healthcare professionals' proposed ehealth needs in elective primary fast-track hip and knee arthroplasty journey: A qualitative interview study. Journal of Clinical Nursing. 2019;28(23-24):4434-46. doi: <http://dx.doi.org/10.1111/jocn.15028>.

274. Massip M, de Batlle J, Barbe F, Torres G, Vargiu E, Miralles F, et al. Implementing Mobile Health-Enabled Integrated Care for Complex Chronic Patients: Patients and Professionals' Acceptability Study. JMIR mHealth and uHealth. 2020;8(11):e22136. doi: <http://dx.doi.org/10.2196/22136>.

275. Parkes RJ, Williams DH, Palmer J, Wingham J. Is virtual clinic follow-up of hip and knee joint replacement acceptable to patients and clinicians? A sequential mixed methods evaluation. BMJ open quality. 2019;8(1):e000502. doi: <http://dx.doi.org/10.1136/bmjoq-2018-000502>.

276. van Kasteren Y, Freyne J, Hussain MS. Total knee replacement and the effect of technology on cocreation for improved outcomes and delivery: Qualitative multi-stakeholder study. Journal of medical Internet research. 2018;20(3). doi: <http://dx.doi.org/10.2196/jmir.7541>.

277. Moffet H, Tousignant M, Corriveau H, Nadeau S, Merette C, Boissy P, et al. Patient Satisfaction with In-Home Telerehabilitation After Total Knee Arthroplasty: Results from a Randomized Controlled Trial. Telemedicine journal and e-health : the official journal of the American Telemedicine Association. 2017;23(2):80-7. doi: <http://dx.doi.org/10.1089/tmj.2016.0060>.

278. Tousignant M, Boissy P, Moffet H, Corriveau H, Cabana F, Marquis F, et al. Patients' satisfaction of healthcare services and perception with in-home telerehabilitation and physiotherapists' satisfaction toward technology for post-knee arthroplasty: An embedded study in a randomized trial. Telemedicine and e-Health. 2011;17(5):376-82. doi: <http://dx.doi.org/10.1089/tmj.2010.0198>.

279. Booth MW, Riegler V, King JS, Barrack RL, Hannon CP. Patients' Perceptions of Remote Monitoring and App-Based Rehabilitation Programs: A Comparison of Total Hip and Knee Arthroplasty. Journal of Arthroplasty. 2023;38(7 Supplement):S39-S43. PMID: 2024317908. doi: <https://dx.doi.org/10.1016/j.arth.2023.04.032>.

280. Cooper DM, Bhuskute N, Walsh G. Exploring the Impact and Acceptance of Wearable Sensor Technology for Pre- and Postoperative Rehabilitation in Knee Replacement Patients: A U.K.-Based Pilot Study. JBJS Open Access. 2022;7(2):e21.00154. PMID: 2018089285. doi: <https://dx.doi.org/10.2106/JBJS.OA.21.00154>.

281. Culliton SE, Bryant DM, Chesworth BM, MacDonald SJ, Hibbert KM. Effect of an e-Learning Tool on Expectations and Satisfaction Following Total Knee Arthroplasty: A Randomized Controlled Trial. Journal of Arthroplasty. 2018;33(7):2153-8. doi: <http://dx.doi.org/10.1016/j.arth.2018.02.040>.

282. Giunta NM, Paladugu PS, Bernstein DN, Makhni MC, Chen AF. Telemedicine Hip and Knee Arthroplasty Experience During COVID-19. Journal of Arthroplasty. 2022;37(8 Supplement):S814-S8.e2. PMID: 2017526644. doi: <https://dx.doi.org/10.1016/j.arth.2022.02.106>.

283. Glinkowski W, Cabaj D, Kostrubała A, Krawczak K, Górecki A. Pre-surgery and post-surgery telerehabilitation for hip and knee replacement - Treatment options review and patient's attitudes towards telerehabilitation2010 2010.

284. Grant S, Blom AW, Craddock I, Whitehouse M, Gooberman-Hill R. Home health monitoring around the time of surgery: Qualitative study of patients' experiences before and after joint replacement. BMJ open. 2019;9(12). doi: 10.1136/bmjopen-2019-032205.

285. Joshi R, Joseph A, Mihandoust S, Madathil KC, Cotten SR. A mobile application-based home assessment tool for patients undergoing joint replacement surgery: A qualitative feasibility study. Applied Ergonomics. 2022;103:103796. PMID: 2018255315. doi: <https://dx.doi.org/10.1016/j.apergo.2022.103796>.

286. Kairy D, Tousignant M, Leclerc N, Cote A-M, Levasseur M. The patient's perspective of in-home telerehabilitation physiotherapy services following total knee arthroplasty. International Journal of Environmental Research and Public Health. 2013;10(9):3998-4011. doi: <http://dx.doi.org/10.3390/ijerph10093998>.

287. LeBrun DG, Malfer C, Wilson M, Carroll KM, Wang MV, Mayman DJ, et al. Telemedicine in an Outpatient Arthroplasty Setting During the COVID-19 Pandemic: Early Lessons from New York City. HSS Journal. 2021;17(1):25-30. doi: 10.1177/1556331620972659.

288. Lee M, Kim J, Yoon BC, Suh D, Son J, Eun S-D. Patient perspectives on virtual reality-based rehabilitation after knee surgery: Importance of level of difficulty. Journal of Rehabilitation Research and Development. 2016;53(2):239-52. doi: <http://dx.doi.org/10.1682/JRRD.2014.07.0164>.

289. Marsh J, Bryant D, MacDonald SJ, Naudie D, Remtulla A, McCalden R, et al. Are patients satisfied with a web-based followup after total joint arthroplasty? Clinical orthopaedics and related research. 2014;472(6):1972-81. doi: <https://dx.doi.org/10.1007/s11999-014-3514-0>.

290. Reid H, Mohammadi S, Watson W, Robillard JM, Crocker M, Westby MD, et al. Patient and Caregiver Perspectives on an eHealth Tool: A Qualitative Investigation of Preferred Formats, Features and Characteristics of a Presurgical eHealth Education Module. Rehabilitation process and outcome. 2021;10:11795727211010501. doi: <https://dx.doi.org/10.1177/11795727211010501>.

291. Russell TG, Buttrum P, Wootton R, Jull GA. Rehabilitation after total knee replacement via low-bandwidth telemedicine: The patient and therapist experience. Journal of Telemedicine and Telecare. 2004;10(SUPPL. 1):85-7. doi: 10.1258/1357633042614384.

292. Wang Q, Lee RL-T, Hunter S, Chan SW-C. Patients' experiences of using a mobile application-based rehabilitation programme after total hip or knee arthroplasty: a qualitative descriptive study. BMC nursing. 2023;22(1):246. doi: <https://dx.doi.org/10.1186/s12912-023-01409-3>.

293. Williams E, Putnam J, Emkes L, Greenwood J. Patient perspectives and numerical evaluation of a COVID secure hybrid rehabilitation programme following knee replacement surgery. Physiotherapy (United Kingdom). 2022;114(Supplement 1):e224-e5. PMID: 2016910325. doi: <https://dx.doi.org/10.1016/j.physio.2021.12.214>.
